# Unlocking the genetic diversity of Creole wheats

**DOI:** 10.1038/srep23092

**Published:** 2016-03-15

**Authors:** Prashant Vikram, Jorge Franco, Juan Burgueño-Ferreira, Huihui Li, Deepmala Sehgal, Carolina Saint Pierre, Cynthia Ortiz, Clay Sneller, Maria Tattaris, Carlos Guzman, Carolina Paola Sansaloni, Guillermo Fuentes-Davila, Matthew Reynolds, Kai Sonders, Pawan Singh, Thomas Payne, Peter Wenzl, Achla Sharma, Navtej Singh Bains, Gyanendra Pratap Singh, José Crossa, Sukhwinder Singh

**Affiliations:** 1 International Maize and Wheat Improvement Center (CIMMYT), Apdo. Postal 6-641, 06600, Mexico DF, Mexico; 2 Departamento de Biometría, Estadística y Computación, Facultad de Agronomía, Udelar, Ruta 3, Km. 363, Paysandú, Uruguay; 3 Institute of Crop Science, CIMMYT-China Office, Chinese Academy of Agricultural Sciences, Beijing 100081, China; 4 The Ohio State University, USA; 5 National Institute of Forestry, Agriculture and Livestock, INIFAP, Mexico; 6 Punjab Agriculture University, Ludhiana, India; 7 Indian Agricultural Research Institute, Pusa, New Delhi-110012, India

## Abstract

Climate change and slow yield gains pose a major threat to global wheat production. Underutilized genetic resources including landraces and wild relatives are key elements for developing high-yielding and climate-resilient wheat varieties. Landraces introduced into Mexico from Europe, also known as Creole wheats, are adapted to a wide range of climatic regimes and represent a unique genetic resource. Eight thousand four hundred and sixteen wheat landraces representing all dimensions of Mexico were characterized through genotyping-by-sequencing technology. Results revealed sub-groups adapted to specific environments of Mexico. Broadly, accessions from north and south of Mexico showed considerable genetic differentiation. However, a large percentage of landrace accessions were genetically very close, although belonged to different regions most likely due to the recent (nearly five centuries before) introduction of wheat in Mexico. Some of the groups adapted to extreme environments and accumulated high number of rare alleles. Core reference sets were assembled simultaneously using multiple variables, capturing 89% of the rare alleles present in the complete set. Genetic information about Mexican wheat landraces and core reference set can be effectively utilized in next generation wheat varietal improvement.

Grain yield of wheat, the world’s staple food crop, is estimated to decrease by ~6% for each °C elevation in temperature^1^. The earth’s average global temperature is increasing at a rate of 0.1 °C per decade^2^, indicating the magnitude of climatic variation. More than one-third of wheat yield variability in the global breadbasket is explained by climate variation^3^. According to an estimate, average wheat yield loss due to climate change during 1981–2002 was 19 Mt/year^4^. In view of these challenges, Food and Agriculture Organization launched its Climate-Smart Agriculture (FAO-CSA) initiative to ensure food security^5^. Furthermore, meeting the expected wheat demand to feed a human population of 9.6 billion by 2050^6^, in the current era of climate change and slow yield gains^7^, would require development of high yielding and climate smart ‘next generation varieties’. A multifaceted approach is required for developing such varieties, with a key role being played by unexploited germplasm including landraces long preserved in gene banks^8^.

Landraces that have been selected over many generations by farmers are the repositories of crop’s genetic diversity, and their value to modern plant breeding is massive^9^. Non-elite natural populations such as landraces harbour rare alleles besides other high- and low-frequency alleles. Alleles present at high frequencies are most likely responsible for the wide adaptation of landraces. On the other hand, crop domestication^10^ and improvement^11^,^12^ is accompanied by a selective advantage of alleles present at a very low frequency in those populations. Classic examples of such rare alleles are Green Revolution genes *Rht1* and *sd1* in wheat and rice, respectively^13^. Landraces may also have rare alleles for disease resistance and tolerance to environmental stresses. The pressing need to cope with climate change and maximize yield gains can be addressed in part through obtaining a combination of both types of alleles from landraces.

Adaptation of wheat landraces in their native environments has resulted in the accumulation of favourable alleles for domestication traits^14^,^15^,^16^. Mexican landraces, also known as Creole wheats, were brought to the Americas from the 16^th^ through 18^th^ centuries and gradually became adapted to the local environments. Their genetic diversity is believed to be depleted in the germplasm collections of Spain and Europe^17^. Mexico has climatic diversity because of its large variety of landscapes, from tropical and temperate forests to desert areas^18^. Broadly dry and tropical climatic regimes prevail in the south-central and northern parts of Mexico, respectively. During the cropping season in some of the northern states (e.g., Durango), temperatures can reach up to 40 °C (Supplementary Figures 1 and 2). Landrace accessions adapted to the varying climates of Mexico should thus have useful genetic variation for stress tolerance. The large-scale introduction into breeding pipelines of the genetic diversity available in these landraces could greatly help in developing next generation varieties, leading to increased global wheat production.

Characterization of a large collection of landraces adapted to wide climatic regimes is an urgent need of the wheat breeders worldwide. An ambitious project of the International Maize and Wheat Improvement Center (CIMMYT), Seeds of Discovery^19^, aims to characterize all the accessions in the Wheat Germplasm Bank (~120,000 accessions) and move the unexploited variation into the breeding pipelines^20^. This is being followed by genotyping using the state-of-the-art technology, genotyping-by-sequencing (GBS) which provides a cost effective platform for genotyping thousands of accessions. Numerous platforms are available for assessing single nucleotide polymorphisms (SNPs) and cost per data point is least in GBS rendering it a suitable platform for large scale germplasm characterization. A total of 9811 accessions collected from different Mexican states during the 1990s are maintained in the CIMMYT wheat germplasm bank^21^. An in-depth and systematic characterization is required to capture the full potential of these valuable genetic resources.

An important step in utilizing gene banks is defining a manageable core reference set as a subset of the larger germplasm collection^22^. A manageable core reference set is the one which breeders can precisely evaluate for their trait (s) of interest. Core reference sets have been established in the past mainly based on one variable^23^,^24^,^25^, for example genotypic data or phenotype measures or geographical distribution. Simultaneous use of multiple types of variables (genotype, phenotype, geography etc.) while classifying a germplasm set should provide a robust diversity estimate for its application in plant breeding. However, merging different types of variables in one matrix is a challenge and researchers are often reluctant to do such analyses because of the limitations of the software tools or methods^26^. Till date there is no report available in wheat wherein core set is developed by using multiple variables. A strategy for utilizing both discrete and continuous variables, as well as combining hierarchical multiple-factor analysis (HMFA) and the two-stage Ward Modified Location Model (Ward-MLM), has been proposed by Franco *et al.*^27^. Gower^28^ proposed a method for simultaneously analyzing continuous and categorical variables by transforming each to a 0-to-1 scale, irrespective of the type of variables. This approach was followed in the present study to define a core reference set of Mexican wheat landraces.

The objectives of this study were to: (1) characterize Mexican wheat landraces conserved in the CIMMYT wheat germplasm bank and (2) develop a core reference set using multiple variables simultaneously.

## Results

Of 8,416 Mexican landraces subjected to analysis, 7,986 were found to be hexaploid based on their genotypic profiles (Supplementary Figure 3), phenotypic evaluation and grain characteristics. Total number of high quality and filtered SNPs used for the study was 20,526 out of which 8,297 were present in frequency less than 0.05. All these markers showed heterozygosity in range of 0–30.5%. The percentage of GBS-based SNPs with allele frequencies ranging from 0.05 to 0.95 was 59.6. The remaining 40.4% had allele frequency <0.05 or >0.95 which enabled us to identify useful genetic variation presented in different sections onwards in this report. Means and variances of phenotypic evaluation and grain characteristics have been presented in Table 1.

### Genetic classification of Mexican hexaploid wheat landraces

**Figure 1:**
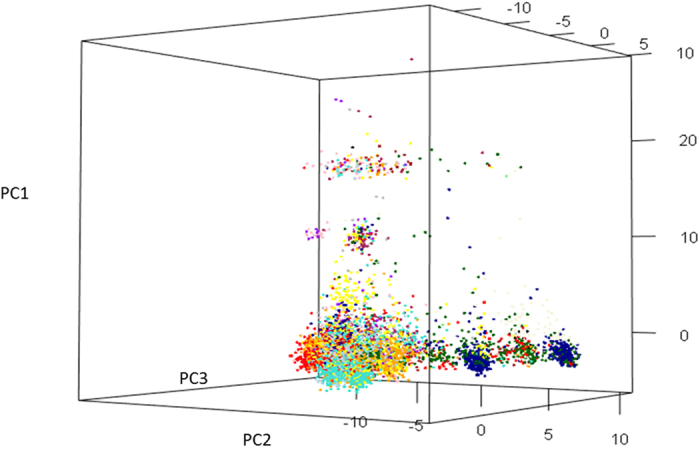
Three-dimensional PCA graph showing the distribution of Mexican hexaploid landrace groups based on genetic classification (using only GBS markers). There were a total of 15 groups that correspond to different Mexican states. 1 = Yellow (MEXICO, PUEBLA), 2 = Light blue (MEXICO, QUERETARO), 3 = Dark blue (CHIHUAHUA, OAXACA), 4 = Orange (MEXICO, PUEBLA, QUERETARO, HIDALGO), 5 = Light green (DURANGO), 6 = Dark green (CHIHUAHUA 95.5), 7 = Pink (OAXACA, TLAXCALA, TOLUCA, PUEBLA), 8 = Purple (OAXACA), 9 = Turquoise (MEXICO), 10 = Brown (MEXICO, MICHOACAN), 11 = Red (COAHUILA), 12 = Gray (TLAXCALA, MEXICO, MICHOACAN), 13 = Maroon (MICHOACAN), 14 = Beige (CHIHUAHUA 95.5), 15 = Black (GUANAJUATO). The PC1, PC2 and PC3 contribute 10.5%, 8.2% and 6.9% of the total variation respectively.

**Figure 2:**
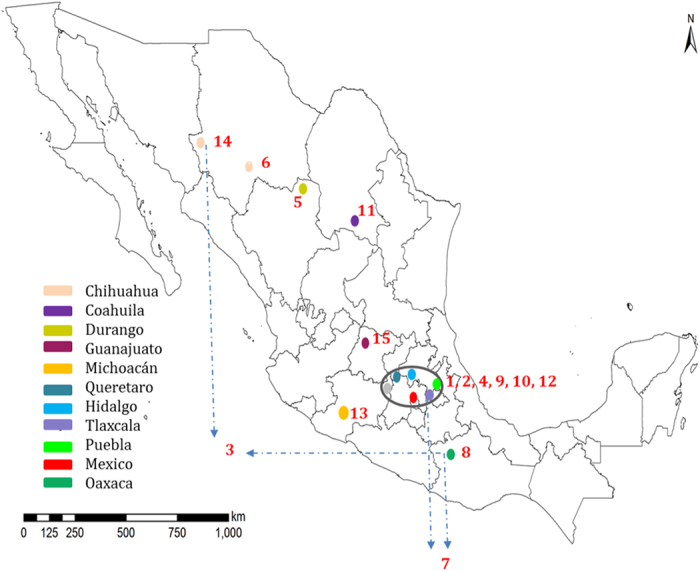
The distribution of 15 genetic groups of Mexican hexaploid landraces in different regions of Mexico. 1 = Yellow (MEXICO, PUEBLA), 2 = Light blue (MEXICO, QUERETARO), 3 = Dark blue (CHIHUAHUA, OAXACA), 4 = Orange (MEXICO, PUEBLA, QUERETARO, HIDALGO), 5 = Light green (DURANGO), 6 = Dark green (CHIHUAHUA), 7 = Pink (OAXACA, TLAXCALA, TOLUCA, PUEBLA), 8 = Purple (OAXACA), 9 = Turquoise (MEXICO), 10 = Brown (MEXICO, MICHOACAN), 11 = Red (COAHUILA), 12 = Gray (TLAXCALA, MEXICO, MICHOACAN), 13 = Maroon (MICHOACAN), 14 = Beige (CHIHUAHUA), 15 = Black (GUANAJUATO).Map was made using ESRI’s ArcGIS Desktop ArcMap 10.2.2 software. On the blank political map, groups were pointed manually using Microsoft power point application.

The principal component analysis (PCA) explicitly revealed broad separation of the northern (Durango, Chihuahua and Coahuila) hexaploid landraces from the accessions of southern Mexico (Oaxaca) and central valley (Mexico, Puebla, Tlaxcala, Queretaro, Toluca, Guanajuato, Hidalgo and Michoacán) with little overlaps (Fig. 1). Genetic classification revealed 15 groups of hexaploid landraces. Thirteen of the 15 groups included accessions from just one region: central, southern, or northern Mexico. Some of the groups had accessions from specific places in Mexico, such as Durango (Group 5), Oaxaca (Group 8), Mexico (Group 9), Coahuila (Group 11), Michoacán (Group 13), Chihuahua (Groups 6 and 14) and Guanajuato (Group 15). Group 3 had accessions from Chihuahua and Oaxaca, located in the extreme northern and southern parts of the country respectively, whereas, Group 7 had lines from the central and southern regions with maximum overlap (Fig. 2). Group 1 showed highest Shannon’s and Nei’s diversity indices followed closely by groups 4, 7, 8, 12 and 13 containing accessions from southern and/or central Mexico. In contrast, group 5 had very low diversity (Table 2). The genetic differentiation (F_st_) among these 15 groups ranged from 0.041 to 0.277. Groups 3, 5, 6 and 14 were genetically the most divergent (Supplementary Table 1).

The genetic diversity analysis of above mentioned 15 groups clustered them into six, referred to as clusters 1–6 (Supplementary Figure 4). Cluster 1 was very distinct from the others and had just one group that contained lines from the northern and southern regions. Cluster 2 contained the largest number of accessions and consisted of lines only from the central region. Clusters 3 and 4 had accessions from two or more regions, while Clusters 5 and 6 had just one homogeneous group each. Accessions from the north appeared in four of six clusters and in five groups. Accessions from the central region appeared in four clusters and nine groups. Accessions from the south appeared in two clusters and just three groups, including Group 8.

### Characterization of the core reference sets

**Figure 3:**
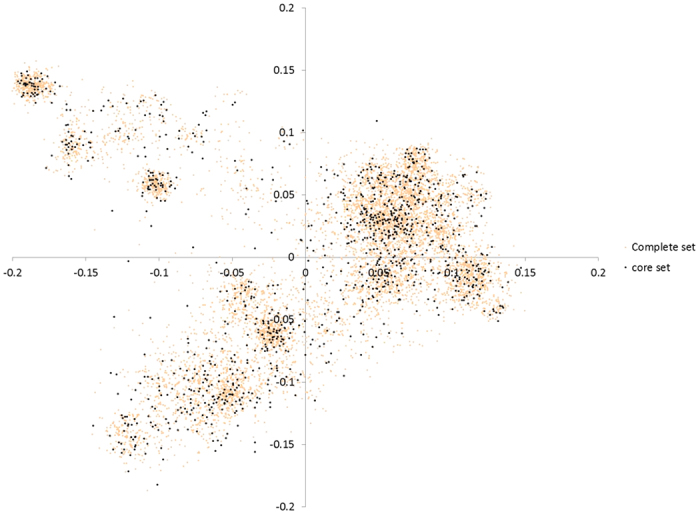
Multidimensional scaling graph showing the relative distribution of complete and core reference set accessions of Mexican hexaploid landraces. Complete and core set entries represented by pink and black dots respectively in the graph.

A core reference set of 1,133 landrace accessions was selected from the complete population (Supplementary Table 2). The mean Gower’s distances of the core reference set and the complete set were 0.093 and 0.088, respectively, showing a genetic diversity gain of 5.5%. Frequency of 158 rare alleles (rare alleles of complete population) increased above 0.05 in the core population (Supplementary Figure 5). Marker allele frequencies (Supplementary Figure 6) and diversity indexes of complete and core sets were comparable (Table 3). Phenotypic means and variances were compared using ratio of complete set/core set (Supplementary Table 3). Range for means and variances were 0.92–1.02 and 0.81–1.04, respectively. Finally, representativeness of core reference set has been shown in multidimensional scaling (MDS) graph in Fig. 3. Overall, comparative analysis of phenotypic variances, diversity measures and allele frequencies among the complete and core reference sets shows that the core reference is a representative of the complete set. The core reference set was also subjected to evaluation for yellow rust disease which led to identify seven resistant landrace accessions with disease severity of 20% or less through screening across two locations- Punjab Agriculture University, India and Toluca, Mexico (Supplementary Table 4).

### Characterization of rare alleles

**Figure 4:**
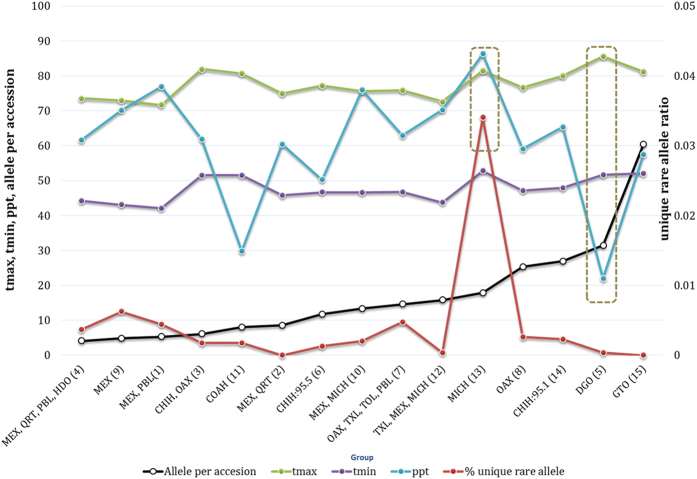
Averaged number of rare alleles per accessions in 15 genetic groups of hexaploid Mexican landraces, average annual temperature (maximum and minimum) and average annual precipitation of regions to which groups belong as well as unique rare allele ratio in each group. Unique rare allele ratio = Number of unique rare allele in a group/total number of rare alleles in the group. Calculation of the average temperature and rainfall was made using climate data of collection site of each accession. Precipitation and temperature data of 50 years (i.e. from 1951 to 2000) was used for estimating the average. The 15 genetic groups have been plotted on ‘X’ axis. Precipitation (ppt), maximum (tmax) and minimum (tmn) and unique rare allele have been presented on ‘Y’ axis. For precipitation data a correction factor of 0.1 was used (e.g 769 mm = 76.9 mm). Units for precipitation and temperature were Millimeter and Fahrenheit.

Marker alleles, with minor allele frequency (MAF) less than 0.05 were considered as rare alleles. Of 20,526 SNPs, 8,539 had frequencies ranging [0, 0.05) and 7,775 alleles had a frequency within (0, 0.05), which were considered as rare alleles (Supplementary Figure 7). The total numbers of rare alleles in the complete and core reference sets were 7,775 (18.94%) and 6,876 (16.74%), respectively. Comparative analysis of the two populations revealed that the core reference set captured 88.75% of the rare alleles of the complete set (Table 3). The average number of rare alleles per accession was estimated for each of the 15 genetic groups. The highest number of rare alleles per accession occurred in the genetic groups belonging to Guanajuato (Group 12) followed by Durango (Group 5), Chihuahua (Group 14), Oaxaca (Group 8) and Michoacán (Group 13), as shown in Table 4 and Fig. 4. Interestingly, a very high percentage of unique rare alleles were observed in the landrace accessions of Michoacán (Group 13).

The rare allele analysis carried out for identifying fixed marker alleles (not segregating) revealed genomic regions dispersed throughout the twenty one chromosomes (Supplementary Figure 8). Maximum fixed alleles were found in accessions from Chihuahua followed by the ones the central valley. List of the marker alleles is presented in Supplementary Table 5. Clustering based on longitude, latitude and altitude, grouped Chihuahua accessions in to one which also corresponding genetic groups 6 and 14 (Supplementary Table 6).

## Discussion

The Green Revolution has been associated with the replacement of traditional varieties and landraces by high-yielding, input-responsive, semi-dwarf varieties of cereal crops^29^,^30^, which significantly contributed to global food security. However, this also led to increased monoculture and the depletion of on-farm varietal diversity, which today poses a serious threat to food production under climate change scenarios. Reinforcement of the genetic variation from underutilized landraces into modern varieties should provide impetus to achieve the targets of proposed climate smart agriculture^5^. Efforts made in the present study are an example of large-scale characterization of germplasm that remained unexploited in gene banks. At the global level, similar approaches for mobilizing gene bank and on-farm diversity to breeding pipelines will make it possible to accomplish the goals of FAO-CSA initiative.

Mexican wheat landraces have been used by researchers in breeding for abiotic stress tolerance and grain quality improvement. Small sets were formed based on the available information (e.g. collection site) and therefore only 25% of entire Mexican landrace collection was utilized^31^,^32^,^33^. Three-fourths of the Mexican landrace collection remained uncharacterized. The present study provides a thorough genetic understanding of Mexican wheat landraces for researchers intending to utilize them. PCA, genetic classification and cluster analysis showed a broad differentiation of landraces belonging to northern and southern Mexico while some of the groups, being adapted to specific regions (Figs 1 and 2). They therefore have more potential for adaptation to a wide range of environments compared to the landraces that are adapted to specific ecosystems. Contrarily, some other landrace groups showed different patterns, for example, regional groups from Oaxaca, Guanajuato, Michoacán, Durango, Coahuila and Chihuahua (Fig. 2). For example, group 3 has accessions from extreme north and south Mexico but group 8 represents only Oaxaca, the southern province but diversity index of group 8 was significantly higher than that of group 3. Above mentioned distinct groups belonging to specific regions showed presence of relatively higher number of rare alleles per accession as compared to other groups (Table 4). Possible reasons for their adaptation could be either their early introduction or development of strong environmental imprints in them. One of the genetic groups representing a specific collection site in Mexico (Michoacán) showed an exceptionally high frequency of unique rare alleles (Fig. 4). Temperature and rainfall were at the higher end of the scale for this site, representing a unique climatic regime. The unique allelic diversity of the Michoacán landraces (Group 13) may be associated with their adaptation to this peculiar climate. Landraces from Durango (Group 5), a region characterized by very high annual average temperatures and low precipitation, had a very high number of rare alleles per accession. Combinations of different rare alleles may be the contributing factor to the adaptation of landraces to dry climates where heat stress and droughts are frequent. An intensive analysis of these two groups (Groups 5 and 13) could unveil the relevance of allelic diversity to climatic adaptation. Additionally, landrace accessions adapted to specific environments could be utilized efficiently in developing varieties for similar target ecosystems. Sources for heat stress tolerance as well as other stresses have been identified from these landraces and being used through large scale pre-breeding efforts (Sukhwinder Singh, CIMMYT, Unpublished). Nevertheless, Mexico is not the center of origin for wheat; landraces are adapted to a wide range of the climatic regimes which render them fit candidates for climate resilient wheat genetic improvement. Further, comparison of their diversity profile with landraces from the Fertile Crescent and Southern Europe will generate useful information regarding their evolutionary history and practical deployment in climate resilience breeding.

Another interesting pattern was observed in landraces from Chihuahua which is situated in extreme north part of the country. Maximum number of accessions with fixed marker alleles was found in Chihuahua followed by Central Valley (Supplementary Figure 8). Gene flow analysis further indicated towards their divergence; however, a definitive test would confirm this (Supplementary Figure 9). There might be two different possibilities, either wheat landraces from different sources would have been introduced to Chihuahua and Central Valley or genomic regions harboring such alleles could have undergone natural selection. Since wheat is introduced almost 400–500 years ago into Mexico by Spanish, the former possibility seems more likely. However, an in-depth analysis of phylogenetic history would confirm about ‘introduction’ and ‘selection imprint’. Adaptation of genetic groups to diverse climatic regions of Mexico, the diversity pattern of these genetic groups (reflected in their diversity indices) and the distribution of rare alleles have provided first-hand information that will allow wheat researchers worldwide to efficiently utilize Mexican landraces in breeding pipelines.

The systematic utilization of the Mexican landrace diversity requires a manageable representative germplasm set. Natural, non-elite landrace populations are known to harbor agronomically important alleles in very low frequencies^13^. Therefore, a core reference set of natural populations with maximized rare alleles will likely unveil genetic variation useful for crop improvement. In the past, wheat core reference sets have been developed using one variable at a time^23^,^24^, resulting in significant reduction of rare allele diversity^24^. In this study, a unique core reference set development strategy was followed, i.e., using an integrated data matrix of both continuous and discrete variables (phenotypic and genotypic), thereby maximizing overall diversity for analysis. In this strategy, we reduced the dimensions of the marker data to 2,000 principal components explaining 84% of the total variance and then merged them to 23 phenotypic variables to form a data matrix in such a way that genotypic and phenotypic contributions were 75% and 25%, respectively. This arbitrary ratio was chosen for enhancing the role of phenotypic variation to core development. Detailed methodology has been explained in ‘materials and methods’ section. Wingen *et al.*^24^ concluded that the core reference set strategy is not useful for discovering very rare alleles, perhaps due to the sample size or low-throughput marker platform. We report a significant enrichment of rare alleles in the core reference set. The percentage of rare alleles in the original landrace population was 18.94%, whereas it was 16.74% in the core reference set, suggesting an 88.75% rare allele recovery in the latter. Furthermore, frequencies of 158 rare alleles (in the complete set) rose above 0.05 in the core reference set (Supplementary Figure 5). This enrichment of rare alleles in the study was due to the base population (7,986 genotypes), high-throughput SNP data ( >20,000 markers), coupled with a unique core reference set development strategy, which simultaneously uses multiple variables. Minor allele frequency (MAF), geographical representation and phenotypic variance of the core reference set were comparable to those of the complete set (Supplementary Figure 6, Tables 3 and Supplementary Table 3).

Characterization of the core reference set enriched with rare alleles is a valuable germplasm resource for trait dissection and gene discovery. This representative population can be efficiently utilized by wheat researchers globally. Core reference set has already been distributed to researchers in Africa, South Asia, and the USA. Its evaluation in two geographically divergent environments, India and Mexico for yellow rust disease has identified resistant genotypes (Supplementary Table 4). Information presented in this study about Mexican wheat landraces will serve as a high value resource base for wheat breeders world-wide to develop high-yielding and climate-resilient next-generation wheat varieties.

### Online Methods

#### Description of plant material

Wheat landraces used in this study were collected from Mexico. A total of 9,811 Mexican wheat landraces were collected from 16 Mexican states as part of a project sponsored by Mexico’s National Commission for the Study and Use of Biodiversity^21^. After eliminating duplicate accessions based on phenotypic information, a total of 8,416 Mexican wheat landraces, including hexaploid and diploid accessions as well as other species, were used in the analysis. A total of 7986 hexaploid landraces were used for genetic analysis and their details have been provided in Supplementary Table 7.

#### Genotypic analysis of Mexican wheat landraces

Seed of a single plant from each accession was used for DNA extraction and seed of the same plant was used for the phenotypic evaluation. Extraction of genomic DNA was carried out by a modified CTAB (cetyltrimethylammonium bromide) method^34^, followed by quantification using NanoDrop 8000 spectrophotometer V 2.1.0. Genotyping was performed through DArT-seq GBS technology (called DArTseq™) at DArT Pyt Ltd, Canberra, Australia^35^. This method follows two-step complexity reductions. In this approach, two enzymes, PstI_ad/TaqI/HpaII and PstI_ad/TaqI/HhaI_ad, along-with TaqI restriction enzyme were used to eliminate subsets of PstI -HpaII and PstI-HhaI fragments, respectively. To encode the DNA samples in plates which were ligated into small restriction fragments, *Pst*I-specific adapters were tagged with 96 different barcodes. These *Pst*I adapters were characterized by a sequencing primer and tags generated after sequencing were read with the help of *Pst*I sites. Restriction products were amplified, quality was checked and then all 96 samples in a plate were pooled. Pooled DNA was run in a single lane on an Illumina Hiseq 2000 instrument for sequencing. To obtain the DArT score tables and SNP tables, a proprietary analytical pipeline developed by DArT P/L was used.

Marker data were filtered based on reproducibility, call rate and the average read depth using the pipeline. Reproducibility was determined by assaying approximately 60% of samples twice. The minimum threshold values for completeness, reproducibility and call rate were kept at 50%, 95% and 85%, respectively. The average read depth was 7. Variants were called within the data by clustering sequences by sequence similarity, thereby avoiding the use of an external reference genome. In this approach, either the most common sequence in the population or a wheat sequence previously recorded by DArT genotyping protocol was considered as the reference. This approach of recalling GBS samples has been used in recent wheat studies with the DArT-seq markers^20^.

A total of 20526 SNPs were recalled from the raw GBS data (Supplementary Data File). These markers had varying numbers of missing scores (Supplementary Figure 10). Of these, 20039 markers with less than 50% missing scores were used in the analysis. Stringency was kept to this level to (1) include a high number of minor/rare alleles in the analysis, and (2) minimize the risk of under-representing a genomic region because the chromosome locations of all GBS tags were not known. Missing scores could represent biological data points (presence and absence variations-PAV). A total of 20039 SNP markers were used in the diversity analysis, in core reference set development and in marker-trait association analysis. For determining the genomic position of markers, two different consensus genetic linkage maps were referred (20, DArT, Australia, unpublished). In order to estimate diversity indices and genetic differentiation (Fst values), we select 301 markers as equally spaced as possible in the chromosome (Supplementary Figure 11). Selection was performed following the next steps: (1) Calculate the percentage of markers per chromosome and determine the number of markers of 301 to preserve the representation of the chromosomes; (2) Calculate the distance between the first and the last marker in each chromosome and divide this amount by the number of markers obtained in 1; (3) Starting from the first marker, calculate the points in the chromosome to have equally spaced markers; and (4) Identify the markers as closest to each point. Those markers were selected in the sample. This analysis was performed using a custom code in SAS v9.4. The set of 301 markers was used. Map in Fig. 2 was made using ESRI’s ArcGIS Desktop ArcMap 10.2.2 software^36^. The dataset used to make the maps on rainfall and temperature was Worldclim 1.4^37^,^38^. 30 s resolution (ca 1 km) long term (1950–2000) monthly average grids for rainfall and minimum, maximum temperature were used to generate annual average grids for rainfall and average temp and mapped subsequently. Geo-referenced locations of origin of land races were converted to vector data and integrated in the maps.

#### Phenotypic analysis of Mexican wheat landraces

Phenotypic characterization of all 8,416 Mexican wheat landraces was carried out in three different environments: well irrigated, drought stress and heat stress. Trials were conducted at the CIMMYT Experiment Station near Ciudad Obregon, Mexico (27 20° N, 109 54° W, 38 m ASL) during the 2011–2012 crop season. The well-irrigated and drought trials were conducted during the spring wheat season (November 2011–April 2012). Trials under heat stress were planted in April 2012 so they were exposed to high temperature stress at anthesis. Each trial was conducted as an augmented design with 0.3 m^2^ plot size; check varieties ‘Vorobey’ and ‘Baj’ were repeated multiple times in the experiment. Appropriate measures were used to fertilize and control weeds, diseases, and pests. In the well-irrigated trial, the plots were watered so that approximately 600 mm of water were applied during the complete wheat cycle. Irrigation was provided to plots whenever approximately 50% of available soil moisture was depleted according to gravimetric scales. Approximately 200 mm of total soil moisture was available during the growing season for the drought treatment. The heat stress treatment was watered the same as the irrigated treatment to avoid the confounding effect of drought.

Seeds for the grain quality analysis were obtained from the irrigated treatment; grain morphological characteristics were evaluated with the digital image system SeedCount SC5000 (Next Instruments, Australia) and thousand-kernel weight (TKW, g), test weight (TW, kg/hl), average grain length and width (mm), as well as percentage of grains affected by yellow berry (%) were determined. Grain size distribution was measured using sieves of 2.8 mm (Screen 1), 2.5 mm (Screen 2) and 2.2 mm (Screen 3). Near-infrared spectroscopy (NIRS, Antaris II FT-Analyzer, Thermo Scientific, USA) was used to determine grain hardness (GH, %) and grain protein content (GP, %). The near-infrared spectroscopy instrument was calibrated based on AACC methods^39^ for particle size index (AACC Method 55–30) and protein (AACC Method 46–11A). Grain protein was adjusted to 12.5% moisture content. Whole-meal flour samples were obtained using a UDY Cyclone mill (0.5 mm sieve). Only one gram of whole-meal flour was used to perform the SDS-sedimentation (SDS, ml) test^40^.

#### Phenotypic screening of Mexican core reference set for yellow rust

Evaluation of the core reference sets for yellow rust was performed under field conditions at CIMMYT’s Experiment Station in Toluca, Mexico (May 2014 to October 2014) and at Punjab Agriculture University, India (October 2014 to March 2015). Accessions were planted in a randomized complete block design with two replicates, in a plot consisting of two one-meter rows. Approximately 60–70 seeds were sown in each plot. Yellow rust susceptible variety Avocet was planted around the whole experimental block. Inoculation of spreader/border rows was done according to the method explained by Hao *et al.*^41^. Disease assessment was performed when the susceptible check variety Avocet showed 100% yellow rust severity (during the mid-dough stage of plant growth). Assessment of percent disease severity was performed according to the modified Cobbs Scale^42^.

#### Cluster and diversity analysis of Mexican landrace accessions

A three-step approach was followed for classifying Mexican wheat landraces. First, we performed principal component analysis (PCA) using only genetic data from all the accessions, including hexaploid, tetraploid and other species. Next, we conducted a genetic diversity analysis of just the hexaploid landraces to define their characteristics with respect to their geographic collection sites. Finally, we used classification to develop a core reference set of hexaploid accessions combining different types of variables (genotypic and phenotypic) using the Hierarchical Multiple Factor Analysis^43^,^44^; and Gower’s distance was used as a measure of genotypic and phenotypic diversity^28^.

#### Principal component analysis of Mexican landrace accessions

Principal component analysis was done with the GBS data using the “princomp” function of R-project version 3.1.1^45^.

#### Cluster and diversity analysis of Mexican hexaploid wheat landrace accessions

Genetic distances were calculated using SAS PROC DISTANCE and cluster analysis was done with the SAS PROC CLUSTER procedures of SAS v9.4^46^. Classification of landrace accessions was done using hierarchical multiple-factor analysis (HMFA) in a step-wise manner, and in every step, one group was split into two groups. We identified final working groups that allowed representation of heterogeneity between individuals and as homogenous as possible considering geographical distribution. A simple matching coefficient transformed into squared Euclidean distance was used to measure the similarity between landraces. Formula used for Euclidean distances was:


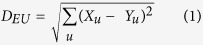


D_EU_ = Euclidean distance; u = individual landraces and X_u_ and Y_u_ are different individuals. The genetic distance between two landraces x and y, denoted as *d*(*x*, *y*), was calculated by one minus the ratio between the number of matches (M) and the total number of non-missing pairs (N), that is,


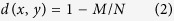


A complete algorithm (used for clustering) was used to estimate the distance between groups based on the greatest distance between any two individuals in different groups. The distance between groups a and b, denoted as *d*(*a*, *b*), was estimated as the maximum distance among all pairs of individuals in different groups:


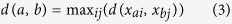


where *x*_*ai*_ represents the individual *i* in group a, and *x*_*bj*_ represents the individual *j* in group b. Allele frequencies were determined with Tassel version 5^47^. Nei’s and Shannon’s distances were calculated to estimate the extent of diversity within the entire population, within each group, and within the core reference set based on Euclidian distances. For Nei’s index, the following formula was used,


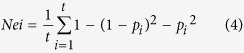


Shannon’s index was calculated as,


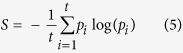


where *P*_*i*_ is the frequency of the major allele of the *i*^th^ marker and *t* is the number of markers.

For estimation of gene flow, the coefficient of gene differentiation (G_ST_) was calculated as:


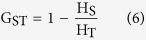


Where H_T_ is the total genetic diversity and H_S_ is the intra-population genetic diversity, both determined through Nei’s genetic diversity statistics^48^. Gene flow (N_m_) was estimated as:


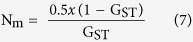


For population differentiation, Wright’s F_st_ values^49^ were calculated using Genepop software^50^. We also performed correlation analysis between sample size and diversity indices to see the effect of sample size on diversity statistics and concluded that in our study sample sizes do not significantly affect the diversity indices (Supplementary Figure 12).

### Rare allele characterization in different populations

Rare alleles were defined for different purposes with respect to populations and presented in qualitative ways i.e. (1) rare allele per unit accession, (2) unique rare alleles and (3) fixed rare alleles in accessions belonging to specific regions.

1. For comparing allele richness in core and complete set complete set of Mexican landrace (7986 individuals) was considered as a population and rare alleles were determined with respect to this population. Richness of these ‘rare alleles’ were then observed in the core set population comprising 1133 individuals (Table 3, Supplementary Figure 5).
2. Secondly, rare alleles were estimated in each of the genetic groups. Each group was considered as a population and rare alleles were defined based on the respective population. These genetic groups were defined based on genetic similarity matrix. Further, in order to explain rare alleles in a more un-biased way we have estimated the, “Unique rare alleles” which is a qualitative measure-not affected by size of the groups (Fig. 4).
3. Further, to identify alleles fixed in groups (groups based on genetic similarity matrix or geology) we first selected list of markers with at least 60% data points showing allele frequency of less than 0.05. Clusters were formed using latitude, longitude and altitude informations with the k-means method. Euclidean distance and least square method were followed for this analysis. A table with cross classification between cluster based on geographical data and 15 genetic groups was created (Supplementary Table 6). Allele frequency was analyzed in environmental clusters to identify the alleles fixed in particular locations. Markers with fixed allele in particular locations (Chihuahua and central valley) were then mapped to the wheat genome using Jim Kent’s blast-like alignment tool (BLAT)^51^. The version 2.26 of the International Wheat Genome Sequencing Consortium was downloaded from Gramene website^52^. All the markers where aligned to each of the chromosomes and filtered using the highest match percentage. Markers showing identity of 95% and above were used for presentation. Based on this information genomic regions have been determined and presented in Supplementary Figure 8.

### Simulation of optimum population size for core set selection

Optimum size of the core reference set was determined through Monte Carlo simulations by 1000 replications. Twenty levels (i.e., 5–100% with step size 5%) of population size were randomly selected from the whole population. For each level of sub-population size, the genetic variance was calculated by the following equations:


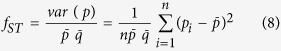



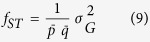


Thus


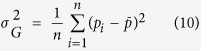


where *f*_ST_ is the statistic to test for allele frequency differences among populations; *p* and *q* are allele frequencies satisfying *p* + *q* = 1; var (*p*) is the variance of allele frequency *p*; *n* is the number of populations, which is equal to the number of simulations in this study; 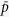 and 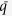 are the mean allele frequencies of *p* and *q*, respectively, across simulation runs, and 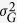 is the genetic variance^53^. A core reference set that contained approximately 15% of the complete set could reach the highest genetic variance among the twenty sub-populations (Supplementary Figure 13). Therefore, 1,133 landrace accessions (14.18%) out of 7,986 accessions in the complete population were selected as the core set.

### Classification of Mexican landraces for core reference set selection

To select the core reference sets, Mexican landraces were classified using the data matrix containing genotypic and phenotypic variables. We first used PCA by the marker data to reduce the dimensions of the marker data to 2,000 principal components (from 20,039) that explained 84% of the total variance. Thereafter the scores of the 2,000 PCA components and the 23 phenotypic variables were merged to form a data matrix that was then used in an HMFA^43^,^44^. Six principal axes (PA) were selected from HMFA in such a way that genotypic and phenotypic contributions to the total variance explained by the six axes were 75% and 25%, respectively. Using this data matrix we estimated the contributions on genotype and phenotype for principal axes (PA) of 1 to 25. The first dimension (or principal axis) explained phenotype: genotype variability ratio of 50:50% whereas, 25^th^ dimension correspond to 8:92% ratio. Six PA explained 25% and 75% phenotype and genotype variability respectively. Similarly, another landrace data set (2403 Iranian landraces) was analyzed which also suggested that 6 PA explain 30% and 70% phenotype and genotype variation respectively (CIMMYT, Unpublished data). Total number of PC dimensions representing 20,526 markers was 2000, whereas only 23 phenotype variables were present and to provide weight to phenotype 25% phenotype contribution was decided. Therefore, the 25:75 ratio (phenotype: Genotype) was not exactly an arbitrary criterion but based on two data sets of two independent genetic populations (Mexican and Iranian wheat landraces). For the HMFA analysis, the FactoMineR library was used in R software^54^. The ratio of genotypic and phenotypic variables was more than a thousand to one, so increasing the phenotype representation ratio of 75:25 was chosen to achieve some equilibrium. The coordinates for each accession on each PA were then used to group accessions following the mixture of normal distributions methodology and the optimum number of groups was selected by maximum likelihood^27^. A total of 21 groups were defined. A predefined number of accessions were selected from each group. The number of accessions per group was estimated by the D-method, proportional to group diversity^55^. Following the stratified random sampling method, 1000 candidate reference sets were created and their diversity was estimated using the average Gower’s distance^28^; the subset showing the maximum average Gower’s distance was selected to be the core reference set. Analyses were performed using different libraries from the open source software R^45^. To visualize whether the core reference set is representative of the complete set, we used a graphical multidimensional scaling (MDS) method. Secondly, we determined the variance of the phenotypic traits in both populations.

## Additional Information

**How to cite this article**: Vikram, P. *et al.* Unlocking the genetic diversity of Creole wheats. *Sci. Rep.* **6**, 23092; doi: 10.1038/srep23092 (2016).

## Supplementary Material

Supplementary Information

Supplementary Table 5

Supplementary Table 7

## Figures and Tables

**Table 1:** Details of phenotype and genotype variables used for analysis.

Trait/Variable	Values	Mean	Variance*	Range	CV (%)	Environment
Total number of SNPs	20526	–	–	–	–	–
Markers with less than 0.05 MAF	8297	–	–	–	–	–
Markers with MAF between 0.05 and 0.95	12229	–	–	–	–	–
Number of monomorphic markers	2	–	–	–	–	–
Number of markers 0% heterozygosity	646	–	–	–	–	–
Heterozygosity range	0–30.5%	–	–	–	–	–
Duration(Early/Medium/Late)	Ordinal variable	2	0.1	–	13.5	irrigated
Biomass at heading	Ordinal variable	2.2	1.2	–	49	Drought
Biomass at maturity	Ordinal variable	2.4	1.1	–	45.4	Drought
Plant height under drought	Ordinal variable	2.9	0.1	–	12.5	Drought
Days to heading under heat	Continuous variable	63.2	50.4	42–96	11.2	Heat
Days to maturity under heat	Continuous variable	82.6	30.1	67–111	6.6	Heat
Plant height under heat (cm)	Continuous variable	55.4	30.7	25–90	10	Heat
Days to heading under drought	Continuous variable	86.9	96.5	60–127	11.3	Drought
Days to maturity under drought	Continuous variable	122.8	51.4	96–140	5.8	Drought
Grain yield under drought (g/m^2^)	Continuous variable	202.3	6103.7	0.53–540	38.6	Drought
Days to heading under irrigated	Continuous variable	99.3	135.4	55–115	11.7	irrigated
Plant height under irrigated (cm)	Continuous variable	146.8	227.8	65–195	10.3	irrigated
Thousand kernel weight (g)	Continuous variable	40.9	30.5	25.4–64.8	13.5	Quality
Test weight (g)	Continuous variable	76.2	8	47.8–83.7	3.7	Quality
Grain length (mm)	Continuous variable	6.8	0.21	5.4–8.29	6.7	Quality
Grain width (mm)	Continuous variable	3.1	0.04	2.5–3.83	6.3	Quality
Screen3-q (mm)	Continuous variable	49.5	697.6	0–94.6	53.4	Quality
Screen2-q (mm)	Continuous variable	22.3	158.3	0–56.3	56.4	Quality
Screen1-q (mm)	Continuous variable	13.8	109.1	0.1–44.2	75.5	Quality
Yellow berry (SeedCount Total %)	Continuous variable	6.9	53.8	0–66.7	106.2	Quality
Grain hardness (% Antaris)	Continuous variable	59.3	19.9	42–74	7.5	Quality
Grain Protein (12.5% MB, Antaris)	Continuous variable	16.1	1.8	12.1–21.2	8.3	Quality
Whole Meal (ml)	Continuous variable	18.5	14.4	25-Jul	20.5	Quality

^*^Variance refers to the total phenotypic variance explained by population.

**Table 2:** Diversity indices of the Euclidean groups of hexaploid Mexican wheat landraces.

Group	Nei	Shannon	Number of accessions	% of accessions from the north	% of accessions from the central region	% of accessions from the south	% of accessions with no information
1	0.26 ± 0.06	0.21 ± 0.02	1227	0.00	100	0.00	0.00
2	0.19 ± 0.06	0.20 ± 0.02	495	0.00	100	0.00	0.00
3	0.12 ± 0.06	0.15 ± 0.02	829	65.86	0.00	34.13	0.00
4	0.25 ± 0.06	0.21 ± 0.02	1445	0.00	100	0.00	0.00
5	0.06 ± 0.06	0.14 ± 0.02	83	100	0.00	0.00	0.00
6	0.19 ± 0.06	0.17 ± 0.02	454	100	0.00	0.00	0.00
7	0.25 ± 0.06	0.21 ± 0.02	372	0.00	26.34	66.66	6.98
8	0.22 ± 0.06	0.20 ± 0.02	195	0.00	0.00	100	0.00
9	0.20 ± 0.06	0.18 ± 0.02	1147	0.00	100	0.00	0.00
10	0.16 ± 0.06	0.18 ± 0.02	290	0.00	100	0.00	0.00
11	0.15 ± 0.06	0.15 ± 0.02	640	100	0.00	0.00	0.00
12	0.22 ± 0.06	0.19 ± 0.02	324	0.00	100	0.00	0.00
13	0.20 ± 0.06	0.20 ± 0.02	287	0.00	100	0.00	0.00
14	0.15 ± 0.06	0.17 ± 0.02	160	100	0.00	0.00	0.00
15	0.11 ± 0.06	0.18 ± 0.02	39	0.00	100	0.00	0.00

**Table 3:** Summary statistics of complete set and the core reference sets of Mexican wheat landraces.

Parameters	Complete set	Core reference set
Number of lost alleles [MAF = 0]	764	1639
Number of rare alleles [MAF < 0.05]	7775	6876
% of rare alleles [MAF < 0.05]	18.94	16.74
% of rare^CS^ allele recovery	100	88.75
% loss of rare^CS^ alleles	0.0	11.25
Number of rare^CS^ allele [MAF < 0.05]	–	158
Shanon-Weaver diversity index	0.21	0.22
Nei’s diversity index	0.27	0.28
Number of accessions	7987	1133
% of accessions from the north	23.49	27.37
% of accessions from the central region	66.7	64.35
% of accessions from the south	9.48	7.84
% of accessions with no information	0.33	0.44

rare^CS^ allele: rare allele of complete set; MAF: minor allele frequency.

**Table 4:** Details of rare^CS^ alleles in 15 genetic groups of Mexican hexaploid landraces.

Group	nra	Nac	raf	napa	nura	pura	Tmax	tmn	ppt
1	6525	1226	0.141	5.32	29	0.444	71.7	42.1	769.8
2	4243	495	0.14	8.57	0	0	74.9	45.8	604.7
3	5075	829	0.141	6.12	9	0.177	81.9	51.5	618.8
4	5965	1445	0.143	4.13	22	0.369	73.5	44.2	617.8
5	2615	83	0.143	31.51	1	0.038	85.6	51.6	220.3
6	5347	454	0.145	11.78	7	0.131	77.2	46.6	502.9
7	5457	372	0.143	14.67	26	0.476	75.8	46.7	629.9
8	4940	195	0.146	25.33	13	0.263	76.6	47.2	591.1
9	5598	1147	0.138	4.88	35	0.625	72.9	43.1	701.6
10	3893	290	0.14	13.42	8	0.205	75.5	46.6	759.9
11	5146	640	0.142	8.04	9	0.175	80.6	51.6	298.6
12	5139	324	0.144	15.86	2	0.039	72.6	43.7	702.8
13	5138	287	0.143	17.9	175	3.406	81.4	52.8	863.5
14	4314	160	0.147	26.96	10	0.232	79.9	47.9	654.2
15	2357	39	0.144	60.44	0	0	81.2	52.1	574.8

rare^CS^ allele: rare allele of complete set; nra = Number of rare alleles; nac = Number of accession; raf = rare allele frequency; apa = Number of rare alleles/accession; nura = Number of unique rare alleles; pura = Percentage of unique rare alleles; tmax = maximum average temperature in degrees Fahrenheit; tmax = minimum average temperature in degrees Fahrenheit; ppt = average annual precipitation. Calculation of the average temperature and rainfall was done using climate data from the collection site of each accession. Precipitation and temperature data of 50 years (i.e., from 1951 to 2000) were used for estimating the averages.
