# Corrigendum: Unlocking the genetic diversity of Creole wheats

**DOI:** 10.1038/srep26216

**Published:** 2016-05-20

**Authors:** Prashant Vikram, Jorge Franco, Juan Burgueño-Ferreira, Huihui Li, Deepmala Sehgal, Carolina Saint Pierre, Cynthia Ortiz, Clay Sneller, Maria Tattaris, Carlos Guzman, Carolina Paola Sansaloni, Marc Ellis, Guillermo Fuentes-Davila, Matthew Reynolds, Kai Sonder, Pawan Singh, Thomas Payne, Peter Wenzl, Achla Sharma, Navtej Singh Bains, Gyanendra Pratap Singh, José Crossa, Sukhwinder Singh

Scientific Reports 6: Article number: 23092; 10.1038/srep23092 published online: 03152016; updated: 05202016.

Mark Ellis was omitted from the author list in the original version of this Article. In addition, there was a typographical error in the spelling of the author Kai Sonder which was incorrectly given as Kai Sonders. These errors have been corrected in the PDF and HTML versions of the Article.

The Acknowledgements section now reads:

The authors duly acknowledge the financial support received from Mexico’s Secretariat of Agriculture, Livestock, Rural Development, Fisheries and Food (SAGARPA) through the Seeds of Discovery-Sustainable Modernization of Traditional Agriculture project (Mas-Agro). We acknowledge Diversity Array Technology (DArT), Canberra, Australia, for the genotyping service provided. Authors extend sincere thanks to Drs Kanwarpal Singh Dhugga, Julio Huerta-Espino, Ky Mathews, Dave Marshall. The direct and indirect support of research technicians is duly acknowledged. We extend our special thanks to Seeds of Discovery project leader and Director of the Genetic Resources Program, Dr. Kevin Pixley, for his valuable support and encouragement to the team.

The Author Contributions section now reads:

S.S. and P.V. conceived and designed the experiments; J.F., J.B., H.L., P.V., D.S. and C.S. performed the diversity, statistical and association analyses; C.S.P., M.R. and M.T. carried out large scale evaluation for heat-drought; C.G. evaluated landraces for grain quality; S.S., C.O., C.P.S. M.E and P.W. contributed to Genotyping; K.S. performed climate data analysis and prepared maps; G.F.D., P.S., A.S., N.S.B., G.P.S., P.V. and S.S. screened core set for diseases; T.P. provided seed material from gene bank; J.C. and S.S. were in-charge to oversee the data collection and analyses; P.V. and S.S. wrote the manuscript and other authors contributed later on; and all authors reviewed the manuscript.

