# Supplementary material for: Unlocking the genetic diversity of Creole wheats
Source: Sci Rep. 2016 Mar 15;6:23092. doi: 10.1038/srep23092 (PMC4791556; doi:10.1038/srep23092)
Supplement: Supplementary Information [file srep23092-s1.pdf]

Unlocking the genetic diversity of Creole wheats

Prashant Vikram[1†], Jorge Franco[2†], Juan Burgueno-Ferrera[1†], Huihui Li[3†], Deepmala Sehgal[1], Carolina Saint Pierre[1], Cynthia Ortiz[1], Clay Sneller[4], Maria Tattaris[1], Carlos Guzman[1], Carolina Paola Sansaloni[1], Guillermo Fuentes-Davila[5], Mathew Reynolds[1], Kai Sonders[1], Pawan Singh[1], Thomas Payne[1], Peter Wenzl[1], Achla Sharma[6], Navtej Singh Bains[6], Gyanendra Pratap Singh[7], José Crossa[1*] & Sukhwinder Singh[1*]

[1]International Maize and Wheat Improvement Center (CIMMYT), Apdo. Postal 6-641, 06600, Mexico DF, Mexico
[2]Departamento de Biometría, Estadística y Computación, Facultad de Agronomía, Udelar
Ruta 3, Km. 363, Paysandú, Uruguay
[3]Institute of Crop Science, CIMMYT-China Office, Chinese Academy of Agricultural Sciences, Beijing 100081, China
[4]The Ohio State University, USA
[5]National Institute of Forestry, Agriculture and Livestock, INIFAP, Mexico
[6]Punjab Agriculture University, Ludhiana, India
[7]Indian Agricultural Research Institute, Pusa, New Delhi-110012, India

*Corresponding authors.

Sukhwinder Singh

Wheat Lead, Seeds of Discovery (SeeD) initiative

International Maize and Wheat Improvement Center (CIMMYT)
Km. 45, Carretera México-Veracruz, El Batán, Texcoco CP 56130
Edo. de México. MEXICO

E-mail: suk.singh@cgiar.org
Mexico City: +52 (55) 5804 2004
Texcoco, México: +52 (595) 952 1900; +52 (595) 952 2104,

José Crossa

Genetic Resources Program,

International Maize and Wheat Improvement Center (CIMMYT)
Km. 45, Carretera México-Veracruz, El Batán, Texcoco CP 56130
Edo. de México. MEXICO

E-mail: j.crossa@cgiar.org
Mexico City: +52 (55) 5804 2004
Texcoco, México: +52 (595) 952 1900; +52 (595) 952 2208,

Supplementary Table 1: Figure showing differentiation (Fst) between 15 Mexican hexaploid landrace groups based on genetic classification (using only GBS markers). There was a total of 15 groups which correspond to different Mexican states. 1= (MEXICO, PUEBLA), 2 = (MEXICO, QUERETARO), 3 = (CHIHUAHUA, OAXACA), 4 = (MEXICO, PUEBLA, QUERETARO, HIDALGO), 5 = (DURANGO), 6 = (CHIHUAHUA 95.5), 7 = (OAXACA, TLAXCALA, TOLUCA, PUEBLA), 8 = (OAXACA), 9 = (MEXICO), 10 = MEXICO, MICHOACAN), 11 = (COAHUILA), 12 = (TLAXCALA, MEXICO, MICHOACAN), 13 = (MICHOACAN), 14 = (CHIHUAHUA 95.5), 15 = (GUANAJUATO).

Group	**1**	**2**	**3**	**4**	**5**	**6**	**7**	**8**	**9**	**10**	**11**	**12**	**13**	**14**
**2**	0.0619													
**3**	0.1991	0.2445												
**4**	0.0429	0.0788	0.1757											
**5**	0.1526	0.1931	0.2557	0.1395										
**6**	0.1856	0.2239	0.0993	0.16	0.236									
**7**	0.0477	0.062	0.1963	0.0571	0.1376	0.2007								
**8**	0.0835	0.0948	0.2336	0.0585	0.1772	0.2192	0.048							
**9**	0.0832	0.0821	0.2768	0.1172	0.2471	0.2677	0.1238	0.1643						
**10**	0.1135	0.0997	0.2047	0.0848	0.1765	0.1922	0.0846	0.0993	0.1818					
**11**	0.1278	0.1697	0.2445	0.1206	0.1445	0.2421	0.0981	0.1502	0.2173	0.1393				
**12**	0.0407	0.1067	0.2131	0.0662	0.1482	0.2075	0.0531	0.1001	0.1452	0.128	0.1106			
**13**	0.079	0.1138	0.1936	0.0817	0.1399	0.1788	0.0556	0.1053	0.1657	0.1112	0.1184	0.0757		
**14**	0.1806	0.2072	0.1367	0.1464	0.2743	0.0571	0.2031	0.2107	0.2586	0.2062	0.2774	0.2215	0.205	
**15**	0.1052	0.1369	0.1604	0.0667	0.2053	0.1136	0.115	0.1227	0.1982	0.1139	0.1829	0.1312	0.1302	0.0888

Supplementary Table 2: List of Mexican landrace accessions with their region information; core set entries are indicated in the table

GID	Core set acc.	Taxon	Region
150889		T.aestivum	South
150892		T.aestivum	South
150893		T.aestivum	South
150899		T.aestivum	South
150926		T.aestivum	South
150927		T.aestivum	South
150929		T.aestivum	South
150930		T.aestivum	South
150931		T.aestivum	South
150932		T.aestivum	South
150934		T.aestivum	South
150935		T.aestivum	South
150938	√	T.aestivum	South
150956	√	T.aestivum	South
150961		T.aestivum	South
150969		T.aestivum	South
150971		T.aestivum	South
150972		T.aestivum	South
150973		T.aestivum	South
150974	√	T.aestivum	South
150975		T.aestivum	South
150978	√	T.aestivum	South
150979		T.aestivum	South
150980	√	T.aestivum	South
150981		T.aestivum	South
150983		T.aestivum	South
150984	√	T.aestivum	South
150985		T.aestivum	South
150986		T.aestivum	South
150987		T.aestivum	South
150988		T.aestivum	South
150989		T.aestivum	South
150990	√	T.aestivum	South
150992		T.aestivum	South
150993		T.aestivum	South
150994		T.aestivum	South
150995		T.aestivum	South

150996		T.aestivum	South
150997	√	T.aestivum	South
150999	√	T.aestivum	South
151000	√	T.aestivum	South
151001		T.aestivum	South
151002		T.aestivum	South
151003		T.aestivum	South
151004		T.aestivum	South
151029		T.aestivum	South
151030		T.aestivum	South
151031		T.aestivum	South
151032		T.aestivum	South
151033		T.aestivum	South
151034	√	T.aestivum	South
151035		T.aestivum	South
151037		T.aestivum	South
151038		T.aestivum	South
151039		T.aestivum	South
151040		T.aestivum	South
151041		T.aestivum	South
151042		T.aestivum	South
151044		T.aestivum	South
151045		T.aestivum	South
151046	√	T.aestivum	South
151047		T.aestivum	South
151048		T.aestivum	South
151049		T.aestivum	South
151050		T.aestivum	South
151051		T.aestivum	South
151052		T.aestivum	South
151053	√	T.aestivum	South
151054		T.aestivum	South
151055		T.aestivum	South
151056		T.aestivum	South
151057		T.aestivum	South
151058		T.aestivum	South
151059		T.aestivum	South
151060		T.aestivum	South
151061		T.aestivum	South
151062		T.aestivum	South
151063		T.aestivum	South

151064	√	T.aestivum	South
151065		T.aestivum	South
151067		T.aestivum	South
151068		T.aestivum	South
151069		T.aestivum	South
151070	√	T.aestivum	South
151071		T.aestivum	South
151072	√	T.aestivum	South
151073		T.aestivum	South
151074		T.aestivum	South
151075		T.aestivum	South
151076		T.aestivum	South
151077		T.aestivum	South
151078		T.aestivum	South
151079		T.aestivum	South
151080	√	T.aestivum	South
151081	√	T.aestivum	South
151082		T.aestivum	South
151083		T.aestivum	South
151084		T.aestivum	South
151085		T.aestivum	South
151086		T.aestivum	South
151087		T.aestivum	South
151088		T.aestivum	South
151089		T.aestivum	South
151090		T.aestivum	South
151091		T.aestivum	South
151092		T.aestivum	South
151093	√	T.aestivum	South
151094		T.aestivum	South
151095	√	T.aestivum	South
151096		T.aestivum	South
151097		T.aestivum	South
151098		T.aestivum	South
151099	√	T.aestivum	South
151100		T.aestivum	South
151101		T.aestivum	South
151102		T.aestivum	South
151104		T.aestivum	South
151105		T.aestivum	South
151106		T.aestivum	South

151107		T.aestivum	South
151109		T.aestivum	South
151110		T.aestivum	South
151111	√	T.aestivum	South
151112		T.aestivum	South
151113		T.aestivum	South
151133		T.aestivum	South
151134		T.aestivum	South
151137		T.aestivum	South
151138		T.aestivum	South
151139	√	T.aestivum	South
151140		T.aestivum	South
151141		T.aestivum	South
151142		T.aestivum	South
151143		T.aestivum	South
151144		T.aestivum	South
151145		T.aestivum	South
151146	√	T.aestivum	South
151147		T.aestivum	South
151148		T.aestivum	South
151149		T.aestivum	South
151150		T.aestivum	South
151151		T.aestivum	South
151152		T.aestivum	South
151153		T.aestivum	South
151154		T.aestivum	South
151155		T.aestivum	South
151156		T.aestivum	South
151157		T.aestivum	South
151158		T.aestivum	South
151159	√	T.aestivum	South
151160	√	T.aestivum	South
151162		T.aestivum	South
151163		T.aestivum	South
151166		T.aestivum	South
151167		T.aestivum	South
151168	√	T.aestivum	South
151169	√	T.aestivum	South
151170		T.aestivum	South
151171		T.aestivum	South
151172		T.aestivum	South

151173		T.aestivum	South
151174		T.aestivum	South
151175		T.aestivum	South
151176		T.aestivum	South
151177		T.aestivum	South
151178		T.aestivum	South
151179		T.aestivum	South
151198	√	T.aestivum	South
151199	√	T.aestivum	South
151202		T.aestivum	South
151203		T.aestivum	South
151204		T.aestivum	South
151205		T.aestivum	South
151206		T.aestivum	South
151207		T.aestivum	South
151208		T.aestivum	South
151209		T.aestivum	South
151210		T.aestivum	South
151213		T.aestivum	South
151214		T.aestivum	South
151215		T.aestivum	South
151216		T.aestivum	South
151217	√	T.aestivum	South
151218		T.aestivum	South
151219		T.aestivum	South
151220	√	T.aestivum	South
151221		T.aestivum	South
151222		T.aestivum	South
151223		T.aestivum	South
151224		T.aestivum	South
151226	√	T.aestivum	South
151227		T.aestivum	South
151230		T.aestivum	South
151231		T.aestivum	South
151256		T.aestivum	South
157526		T.aestivum	South
157528		T.aestivum	South
157529		T.aestivum	South
157530		T.aestivum	South
157531	√	T.aestivum	South
157532		T.aestivum	South

157533		T.aestivum	South
157534		T.aestivum	South
157535		T.aestivum	South
157536		T.aestivum	South
157537		T.aestivum	South
157538		T.aestivum	South
157539		T.aestivum	South
157540		T.aestivum	South
157541		T.aestivum	South
157542		T.aestivum	South
157543		T.aestivum	South
157544		T.aestivum	South
157545		T.aestivum	South
157546		T.aestivum	South
157547		T.aestivum	South
157548		T.aestivum	South
157549		T.aestivum	South
157550		T.aestivum	South
157551		T.aestivum	South
157552		T.aestivum	South
157553		T.aestivum	South
157554		T.aestivum	South
157555		T.aestivum	South
157556		T.aestivum	South
157557		T.aestivum	South
157558		T.aestivum	South
157559		T.aestivum	South
157560		T.aestivum	South
157561		T.aestivum	South
157562		T.aestivum	South
157563		T.aestivum	South
157564		T.aestivum	South
157565		T.aestivum	South
157566		T.aestivum	South
157567		T.aestivum	South
157568		T.aestivum	South
157569		T.aestivum	South
157570		T.aestivum	South
157571		T.aestivum	South
157572		T.aestivum	South
157573		T.aestivum	South

157574		T.aestivum	South
157575		T.aestivum	South
157576		T.aestivum	South
157577	√	T.aestivum	South
157578		T.aestivum	South
157579		T.aestivum	South
157580		T.aestivum	South
157581		T.aestivum	South
157582		T.aestivum	South
157583		T.aestivum	South
157585		T.aestivum	South
157586		T.aestivum	South
157587		T.aestivum	South
157588		T.aestivum	South
157590		T.aestivum	South
157592		T.aestivum	South
157593	√	T.aestivum	South
157594		T.aestivum	South
157596		T.aestivum	South
157597		T.aestivum	South
157598		T.aestivum	South
157599		T.aestivum	South
157600		T.aestivum	South
157601		T.aestivum	South
157602		T.aestivum	South
157603		T.aestivum	South
157604		T.aestivum	South
157605		T.aestivum	South
157607	√	T.aestivum	Central
157608		T.aestivum	Central
157609		T.aestivum	Central
157610		T.aestivum	Central
157611		T.aestivum	Central
157612		T.aestivum	Central
157613		T.aestivum	Central
157614		T.aestivum	Central
157615		T.aestivum	Central
157617		T.aestivum	Central
157618		T.aestivum	Central
157619		T.aestivum	Central
157620		T.aestivum	Central

157622		T.aestivum	Central
157623		T.aestivum	Central
157624		T.aestivum	
157625		T.aestivum	
157626		T.aestivum	
157627	√	T.aestivum	
157628		T.aestivum	
157629	√	T.aestivum	South
157630		T.aestivum	South
157631	√	T.aestivum	South
157632		T.aestivum	Central
157633		T.aestivum	Central
157634		T.aestivum	Central
157635		T.aestivum	Central
157636		T.aestivum	Central
157637		T.aestivum	Central
157638	√	T.aestivum	Central
157639		T.aestivum	South
157640		T.aestivum	South
157644		T.aestivum	South
157645		T.aestivum	South
157646		T.aestivum	South
157647		T.aestivum	South
157648		T.aestivum	South
157649		T.aestivum	South
157650		T.aestivum	South
157651	√	T.aestivum	South
157652		T.aestivum	South
157653		T.aestivum	South
157656		T.aestivum	South
157657		T.aestivum	South
157658		T.aestivum	South
157659		T.aestivum	South
157660		T.aestivum	South
157661		T.aestivum	South
157662		T.aestivum	South
157663	√	T.aestivum	South
157664	√	T.aestivum	South
157665		T.aestivum	South
157666		T.aestivum	South
157667		T.aestivum	South

157668		T.aestivum	South
157669		T.aestivum	South
157670		T.aestivum	South
157671		T.aestivum	South
157672	√	T.aestivum	South
157673		T.aestivum	South
157674		T.aestivum	South
157682		T.aestivum	South
157684		T.aestivum	South
157691		T.aestivum	South
157692		T.aestivum	South
157693		T.aestivum	South
157694		T.aestivum	South
157695		T.aestivum	South
157696		T.aestivum	South
157697		T.aestivum	South
157698		T.aestivum	South
157700		T.aestivum	South
157701		T.aestivum	South
157702		T.aestivum	South
157704	√	T.aestivum	South
157705		T.aestivum	South
157706		T.aestivum	South
157707	√	T.aestivum	South
157708		T.aestivum	South
157709		T.aestivum	South
157710		T.aestivum	South
157711		T.aestivum	South
157712		T.aestivum	South
157713		T.aestivum	South
157714		T.aestivum	South
157715		T.aestivum	South
157716		T.aestivum	South
157717		T.aestivum	South
157719		T.aestivum	South
157720	√	T.aestivum	South
157721		T.aestivum	South
157723		T.aestivum	South
157724		T.aestivum	South
157725		T.aestivum	South
157729		T.aestivum	South

157730		T.aestivum	South
157731	√	T.aestivum	South
157732		T.aestivum	South
157733		T.aestivum	South
157735		T.aestivum	South
157736		T.aestivum	South
157738	√	T.aestivum	South
157739		T.aestivum	South
157741	√	T.aestivum	South
157742		T.aestivum	South
157743		T.aestivum	South
157744		T.aestivum	South
157746		T.aestivum	South
157747		T.aestivum	South
157748		T.aestivum	South
157749		T.aestivum	South
157750		T.aestivum	South
157751		T.aestivum	South
157752	√	T.aestivum	South
157753		T.aestivum	South
157754		T.aestivum	South
157755		T.aestivum	South
157756		T.aestivum	South
157757		T.aestivum	South
157758		T.aestivum	South
157759		T.aestivum	South
157760		T.aestivum	South
157761		T.aestivum	South
157762		T.aestivum	South
157763		T.aestivum	South
157764		T.aestivum	South
157765		T.aestivum	South
157766		T.aestivum	South
157767		T.aestivum	South
157769		T.aestivum	South
157770		T.aestivum	South
157771		T.aestivum	South
157772		T.aestivum	South
157773		T.aestivum	South
157774		T.aestivum	South
157775	√	T.aestivum	South

157776		T.aestivum	South
157777		T.aestivum	South
157778		T.aestivum	South
157779		T.aestivum	South
157780		T.aestivum	South
157781		T.aestivum	South
157782		T.aestivum	South
157783		T.aestivum	South
157785		T.aestivum	South
157786		T.aestivum	South
157789		T.aestivum	South
157790		T.aestivum	South
157791		T.aestivum	South
157792	√	T.aestivum	South
157793	√	T.aestivum	South
157794	√	T.aestivum	South
157795		T.aestivum	South
157812		T.aestivum	South
157819	√	T.aestivum	South
157834		T.aestivum	South
157836		T.aestivum	South
157837		T.aestivum	South
157841		T.aestivum	South
157842		T.aestivum	South
157844	√	T.aestivum	South
157845		T.aestivum	South
157849		T.aestivum	South
157851		T.aestivum	South
157853		t.aestivum	South
157858	√	T.aestivum	South
157863	√	T.aestivum	South
157867		T.aestivum	South
157868		T.aestivum	South
157869	√	T.aestivum	South
157870		T.aestivum	South
157871	√	T.aestivum	South
157872		T.aestivum	South
157874		T.aestivum	South
157910		T.aestivum	South
157916		T.aestivum	South
157921		T.aestivum	South

157922		T.aestivum	South
157930		T.aestivum	South
157935		T.aestivum	South
157940		T.aestivum	South
157952		T.aestivum	South
157953		T.aestivum	South
157954		T.aestivum	South
157955		T.aestivum	South
157961		T.aestivum	South
157962	√	T.aestivum	South
157964		T.aestivum	South
157967		T.aestivum	South
157968		T.aestivum	South
157969		T.aestivum	South
157970		T.aestivum	South
157971		T.aestivum	South
157972		T.aestivum	South
157973		T.aestivum	South
157974		T.aestivum	South
157975		T.aestivum	South
157976		T.aestivum	South
157977		T.aestivum	South
157979		T.aestivum	South
157980		T.aestivum	South
157981		T.aestivum	South
157983		T.aestivum	Central
157984		T.aestivum	Central
157986		T.aestivum	Central
157987		T.aestivum	Central
157988		T.aestivum	Central
157989	√	T.aestivum	Central
157990		T.aestivum	Central
157992	√	T.aestivum	Central
157993		T.aestivum	Central
157994		T.aestivum	Central
157995		T.aestivum	Central
157996		T.aestivum	Central
157997		T.aestivum	Central
157998		T.aestivum	Central
157999		T.aestivum	Central
158001	√	T.aestivum	Central

158002		T.aestivum	Central
158003	√	T.aestivum	Central
158004		T.aestivum	Central
158005		T.aestivum	Central
158006		T.aestivum	Central
158007		T.aestivum	Central
158008		T.aestivum	Central
158009		T.aestivum	Central
158010		T.aestivum	Central
158012		T.aestivum	Central
158014		T.aestivum	Central
158015	√	T.aestivum	Central
158016		T.aestivum	Central
158018	√	T.aestivum	Central
158019		T.aestivum	Central
158020		T.aestivum	Central
158021		T.aestivum	Central
158022		T.aestivum	Central
158023		T.aestivum	Central
158025		T.aestivum	Central
158026		T.aestivum	Central
158027		T.aestivum	Central
158028		T.aestivum	Central
158029		T.aestivum	Central
158030	√	T.aestivum	Central
158031		T.aestivum	Central
158032		T.aestivum	Central
158033		T.aestivum	Central
158034		T.aestivum	Central
158035		T.aestivum	Central
158036		T.aestivum	Central
158037		T.aestivum	Central
158038		T.aestivum	Central
158039		T.aestivum	Central
158040		T.aestivum	Central
158041		T.aestivum	Central
158043		T.aestivum	Central
158044		T.aestivum	Central
158045		T.aestivum	Central
158046		T.aestivum	Central
158048	√	T.aestivum	Central

158049		T.aestivum	Central
158050		T.aestivum	Central
158051		T.aestivum	Central
158052		T.aestivum	Central
158053		T.aestivum	Central
158054		T.aestivum	Central
158055		T.aestivum	Central
158056		T.aestivum	Central
158057		T.aestivum	Central
158058		T.aestivum	Central
158060	√	T.aestivum	Central
158063		T.aestivum	South
158064		T.aestivum	South
158100		T.aestivum	Central
158101		T.aestivum	Central
158102		T.aestivum	Central
158103		T.aestivum	Central
158104		T.aestivum	Central
158105		T.aestivum	Central
158106		T.aestivum	Central
158107	√	T.aestivum	Central
158108		T.aestivum	Central
158109		T.aestivum	Central
158110	√	T.aestivum	Central
158111		T.aestivum	Central
158112		T.aestivum	Central
158113	√	T.aestivum	Central
158114		T.aestivum	Central
158115		T.aestivum	Central
158116		T.aestivum	Central
158117		T.aestivum	Central
158118		T.aestivum	Central
158119		T.aestivum	Central
158120		T.aestivum	Central
158121		T.aestivum	Central
158122		T.aestivum	Central
158123		T.aestivum	Central
158124		T.aestivum	Central
158517		T.aestivum	North
158518	√	T.aestivum	North
158519		T.aestivum	North

158520		T.aestivum	North
158522		T.aestivum	North
158524		T.aestivum	North
158525		T.aestivum	North
158526		T.aestivum	North
158528		T.aestivum	North
158529		T.aestivum	North
158531		T.aestivum	North
158532		T.aestivum	North
158534		T.aestivum	North
158535		T.aestivum	North
158536	√	T.aestivum	North
158537	√	T.aestivum	North
158538		T.aestivum	North
158539		T.aestivum	North
158540		T.aestivum	North
158541		T.aestivum	North
158542		T.aestivum	North
158543	√	T.aestivum	North
158545	√	T.aestivum	North
158546		T.aestivum	North
158547		T.aestivum	North
158548		T.aestivum	North
158550		T.aestivum	North
158551		T.aestivum	North
158554		T.aestivum	North
158555		T.aestivum	North
158556		T.aestivum	North
158557		T.aestivum	North
158558		T.aestivum	North
158561		T.aestivum	North
158562		T.aestivum	North
158563		T.aestivum	North
158564	√	T.aestivum	North
158565		T.aestivum	North
158566		T.aestivum	North
158567		T.aestivum	North
158568		T.aestivum	North
158570		T.aestivum	North
158571		T.aestivum	North
158572		T.aestivum	North

158573		T.aestivum	North
158574		T.aestivum	North
158575		T.aestivum	North
158576		T.aestivum	North
158577		T.aestivum	North
158578		T.aestivum	North
158579	√	T.aestivum	North
158581		T.aestivum	North
158582	√	T.aestivum	North
158583	√	T.aestivum	North
158584		T.aestivum	North
158585		T.aestivum	North
158586		T.aestivum	North
158587		T.aestivum	North
158588		T.aestivum	North
158589	√	T.aestivum	North
158590		T.aestivum	North
158591		T.aestivum	North
158592		T.aestivum	North
158593	√	T.aestivum	North
158594		T.aestivum	North
158595		T.aestivum	North
158596		T.aestivum	North
158597		T.aestivum	North
158598	√	T.aestivum	North
158599		T.aestivum	North
158600		T.aestivum	North
158601		T.aestivum	North
158603		T.aestivum	North
158604	√	T.aestivum	North
158605		T.aestivum	North
158606		T.aestivum	North
158607		T.aestivum	North
158608		T.aestivum	North
158609		T.aestivum	North
158610		T.aestivum	North
158611		T.aestivum	North
158612		T.aestivum	North
158613		T.aestivum	North
158614		T.aestivum	North
158615		T.aestivum	North

158616		T.aestivum	North
158617		T.aestivum	North
158618		T.aestivum	North
158620		T.aestivum	North
158621		T.aestivum	North
158622		T.aestivum	North
158623		T.aestivum	North
158624		T.aestivum	North
158625		T.aestivum	North
158626		T.aestivum	North
158627		T.aestivum	North
158628	√	T.aestivum	North
158629		T.aestivum	North
158630		T.aestivum	North
158631		T.aestivum	North
158632	√	T.aestivum	North
158633		T.aestivum	North
158634		T.aestivum	North
158635	√	T.aestivum	North
158636		T.aestivum	North
158638		T.aestivum	North
158639		T.aestivum	North
158640	√	T.aestivum	North
158641	√	T.aestivum	North
158642		T.aestivum	North
158643		T.aestivum	North
158644		T.aestivum	North
158645		T.aestivum	North
158646	√	T.aestivum	North
158647		T.aestivum	North
158648		T.aestivum	North
158649		T.aestivum	North
158650		T.aestivum	North
158651		T.aestivum	North
158653	√	T.aestivum	North
158654		T.aestivum	North
158655		T.aestivum	North
158656		T.aestivum	North
158657		T.aestivum	North
158658		T.aestivum	North
158660		T.aestivum	North

158661		T.aestivum	North
158662	√	T.aestivum	North
158663		T.aestivum	North
158664		T.aestivum	North
158665		T.aestivum	North
158666		T.aestivum	North
158667		T.aestivum	North
158670		T.aestivum	North
158671		T.aestivum	North
158672	√	T.aestivum	North
158673	√	T.aestivum	North
158674		T.aestivum	North
158675		T.aestivum	North
158676	√	T.aestivum	North
158677	√	T.aestivum	North
158678		T.aestivum	North
158679		T.aestivum	North
158680		T.aestivum	North
158681		T.aestivum	North
158682		T.aestivum	North
158685		T.aestivum	North
158686		T.aestivum	North
158687		T.aestivum	North
158689		T.aestivum	North
158690		T.aestivum	North
158691		T.aestivum	North
158692		T.aestivum	North
158693		T.aestivum	North
158694		T.aestivum	North
158695		T.aestivum	North
158696		T.aestivum	North
158697		T.aestivum	North
158698		T.aestivum	North
158699	√	T.aestivum	North
158701		T.aestivum	North
158702		T.aestivum	North
158703		T.aestivum	North
158704	√	T.aestivum	North
158705		T.aestivum	North
158706		T.aestivum	North
158707		T.aestivum	North

158708		T.aestivum	North
158709		T.aestivum	North
158710		T.aestivum	North
158711		T.aestivum	North
158713		T.aestivum	North
158715	√	T.aestivum	North
158716		T.aestivum	North
158717		T.aestivum	North
158720	√	T.aestivum	North
158721		T.aestivum	North
158722	√	T.aestivum	North
158723		T.aestivum	North
158724		T.aestivum	North
158725		T.aestivum	North
158726		T.aestivum	North
158727		T.aestivum	North
158728		T.aestivum	North
158729		T.aestivum	North
158730	√	T.aestivum	North
158731		T.aestivum	North
158732		T.aestivum	North
158733		T.aestivum	North
158734		T.aestivum	North
158735		T.aestivum	North
158736		T.aestivum	North
158737		T.aestivum	North
158738		T.aestivum	North
158739		T.aestivum	North
158740		T.aestivum	North
158741		T.aestivum	North
158742	√	T.aestivum	North
158743	√	T.aestivum	North
158744		T.aestivum	North
158745	√	T.aestivum	North
158746		T.aestivum	North
158747		T.aestivum	North
158748		T.aestivum	North
158749		T.aestivum	North
158750		T.aestivum	North
158751		T.aestivum	North
158753	√	T.aestivum	North

158754		T.aestivum	North
158755	√	T.aestivum	North
158757	√	T.aestivum	North
158758	√	T.aestivum	North
158759		T.aestivum	North
158760		T.aestivum	North
158762		T.aestivum	North
158763	√	T.aestivum	North
158765		T.aestivum	North
158767		T.aestivum	North
158768		T.aestivum	North
158769		T.aestivum	North
158770		T.aestivum	North
158771	√	T.aestivum	North
158773		T.aestivum	North
158774	√	T.aestivum	North
158775		T.aestivum	North
158776		T.aestivum	North
158777		T.aestivum	North
158778		T.aestivum	North
158779		T.aestivum	North
158780		T.aestivum	North
158781		T.aestivum	North
158782	√	T.aestivum	North
158783		T.aestivum	North
158784		T.aestivum	North
158785		T.aestivum	North
158786		T.aestivum	North
158787	√	T.aestivum	North
158788		T.aestivum	North
158789	√	T.aestivum	North
158790		T.aestivum	North
158791		T.aestivum	North
158792		T.aestivum	North
158793		T.aestivum	North
158794		T.aestivum	North
158795		T.aestivum	North
158796		T.aestivum	North
158797		T.aestivum	North
158798		T.aestivum	North
158799		T.aestivum	North

158800		T.aestivum	North
158801		T.aestivum	North
158802		T.aestivum	North
158803	√	T.aestivum	North
158804		T.aestivum	North
158805		T.aestivum	North
158806		T.aestivum	North
158807		T.aestivum	North
158808	√	T.aestivum	North
158810		T.aestivum	North
158811		T.aestivum	North
158812		T.aestivum	North
158813		T.aestivum	North
158814	√	T.aestivum	North
158815		T.aestivum	North
158816	√	T.aestivum	North
158817		T.aestivum	North
158818		T.aestivum	North
158819		T.aestivum	North
158820		T.aestivum	North
158821		T.aestivum	North
158822		T.aestivum	North
158823		T.aestivum	North
158824		T.aestivum	North
158825		T.aestivum	North
158826		T.aestivum	North
158827		T.aestivum	North
158828		T.aestivum	North
158829		T.aestivum	North
158830		T.aestivum	North
158831		T.aestivum	North
158832		T.aestivum	North
158833		T.aestivum	North
158834		T.aestivum	North
158835		T.aestivum	North
158836		T.aestivum	North
158837		T.aestivum	North
158838	√	T.aestivum	North
158839	√	T.aestivum	North
158840	√	T.aestivum	North
158842	√	T.aestivum	North

158843		T.aestivum	North
158845		T.aestivum	North
158846		T.aestivum	North
158847		T.aestivum	North
158848	√	T.aestivum	North
158849		T.aestivum	North
158850		T.aestivum	North
158851		T.aestivum	North
158852	√	T.aestivum	North
158853		T.aestivum	North
158854		T.aestivum	North
158855		T.aestivum	North
158856		T.aestivum	North
158857		T.aestivum	North
158858		T.aestivum	North
158859		T.aestivum	North
158865	√	T.aestivum	North
158867		T.aestivum	North
158868		T.aestivum	North
158869		T.aestivum	North
158870		T.aestivum	North
158871		T.aestivum	North
158872		T.aestivum	North
158873		T.aestivum	North
158874	√	T.aestivum	North
158875		T.aestivum	North
158876		T.aestivum	North
158877		T.aestivum	North
158878		T.aestivum	North
158879		T.aestivum	North
158880	√	T.aestivum	North
158881	√	T.aestivum	North
158882		T.aestivum	North
158883		T.aestivum	North
158884		T.aestivum	North
158885		T.aestivum	North
158886		T.aestivum	North
158887		T.aestivum	North
158888		T.aestivum	North
158889	√	T.aestivum	North
158890		T.aestivum	North

158891		T.aestivum	North
158892		T.aestivum	North
158893		T.aestivum	North
158894		T.aestivum	North
158895		T.aestivum	North
158896		T.aestivum	North
158897		T.aestivum	North
158899		T.aestivum	North
158900		T.aestivum	North
158901		T.aestivum	North
158902		T.aestivum	North
158903		T.aestivum	North
158904		T.aestivum	North
158905		T.aestivum	North
158906		T.aestivum	North
158907		T.aestivum	North
158908	√	T.aestivum	North
158909		T.aestivum	North
158910		T.aestivum	North
158911		T.aestivum	North
158912		T.aestivum	North
158913		T.aestivum	North
158914	√	T.aestivum	North
158915		T.aestivum	North
158916		T.aestivum	North
158917		T.aestivum	North
158919		T.aestivum	North
158920		T.aestivum	North
158922		T.aestivum	North
158924		T.aestivum	North
158926		T.aestivum	North
158927		T.aestivum	North
158928		T.aestivum	North
158929		T.aestivum	North
158930		T.aestivum	North
158931	√	T.aestivum	North
158932	√	T.aestivum	North
158933	√	T.aestivum	North
158934		T.aestivum	North
158935		T.aestivum	North
158936		T.aestivum	North

158937		T.aestivum	North
158938		T.aestivum	North
158939		T.aestivum	North
158940		T.aestivum	North
158941		T.aestivum	North
158942		T.aestivum	North
158944		T.aestivum	North
158945		T.aestivum	North
158946		T.aestivum	North
158947		T.aestivum	North
158948		T.aestivum	North
158949		T.aestivum	North
158950		T.aestivum	North
158951		T.aestivum	North
158952		T.aestivum	North
158954		T.aestivum	North
158955		T.aestivum	North
158957		T.aestivum	North
158958	√	T.aestivum	North
158959		T.aestivum	North
158960		T.aestivum	North
158961		T.aestivum	North
158962		T.aestivum	North
158963		T.aestivum	North
158964	√	T.aestivum	North
158965		T.aestivum	North
158966		T.aestivum	North
158967		T.aestivum	North
158968		T.aestivum	North
158969		T.aestivum	North
158970		T.aestivum	North
158971		T.aestivum	North
158972		T.aestivum	North
158975		T.aestivum	North
158976		T.aestivum	North
158977		T.aestivum	North
158978		T.aestivum	North
158979		T.aestivum	North
158980		T.aestivum	North
158981		T.aestivum	North
158982		T.aestivum	North

158983		T.aestivum	North
158984		T.aestivum	North
158985		T.aestivum	North
158986		T.aestivum	North
158987		T.aestivum	North
158988		T.aestivum	North
158989	√	T.aestivum	North
158990		T.aestivum	North
158991		T.aestivum	North
158992	√	T.aestivum	North
158993		T.aestivum	North
158994		T.aestivum	North
158995		T.aestivum	North
158996	√	T.aestivum	North
158997		T.aestivum	North
158998		T.aestivum	North
159000		T.aestivum	North
159001		T.aestivum	North
159002		T.aestivum	North
159003		T.aestivum	North
159004		T.aestivum	North
159005	√	T.aestivum	North
159006	√	T.aestivum	North
159007		T.aestivum	North
159008		T.aestivum	North
159009		T.aestivum	North
159010	√	T.aestivum	North
159011		T.aestivum	North
159012		T.aestivum	North
159013		T.aestivum	North
159014		T.aestivum	North
159015		T.aestivum	North
159016		T.aestivum	North
159017		T.aestivum	North
159018		T.aestivum	North
159019		T.aestivum	North
159020		T.aestivum	North
159022		T.aestivum	North
159023		T.aestivum	North
159024		T.aestivum	North
159025		T.aestivum	North

159026		T.aestivum	North
159027		T.aestivum	North
159028		T.aestivum	North
159029		T.aestivum	North
159030		T.aestivum	North
159031	√	T.aestivum	North
159032		T.aestivum	North
159033	√	T.aestivum	North
159034		T.aestivum	North
159035		T.aestivum	North
159036		T.aestivum	North
159037		T.aestivum	North
159038		T.aestivum	North
159039		T.aestivum	North
159040		T.aestivum	North
159041		T.aestivum	North
159042		T.aestivum	North
159043		T.aestivum	North
159044	√	T.aestivum	North
159045		T.aestivum	North
159046		T.aestivum	North
159047		T.aestivum	North
159048	√	T.aestivum	North
159050	√	T.aestivum	North
159051		T.aestivum	North
159052		T.aestivum	North
159053		T.aestivum	North
159054		T.aestivum	North
159055		T.aestivum	North
159056		T.aestivum	North
159057	√	T.aestivum	North
159058		T.aestivum	North
159059	√	T.aestivum	North
159060		T.aestivum	North
159061		T.aestivum	North
159062		T.aestivum	North
159063		T.aestivum	North
159064		T.aestivum	North
159065		T.aestivum	North
159066		T.aestivum	North
159067		T.aestivum	North

159068	√	T.aestivum	North
159069		T.aestivum	North
159070		T.aestivum	North
159071		T.aestivum	North
159072		T.aestivum	North
159073		T.aestivum	North
159074		T.aestivum	North
159075		T.aestivum	North
159076		T.aestivum	North
159077		T.aestivum	North
159078		T.aestivum	North
159079		T.aestivum	North
159080		T.aestivum	North
159081		T.aestivum	North
159082		T.aestivum	North
159083		T.aestivum	North
159084		T.aestivum	North
159085		T.aestivum	North
159086		T.aestivum	North
159088		T.aestivum	North
159089	√	T.aestivum	North
159090		T.aestivum	North
159091		T.aestivum	North
159092		T.aestivum	North
159093		T.aestivum	North
159094		T.aestivum	North
159095		T.aestivum	North
159096		T.aestivum	North
159097		T.aestivum	North
159098		T.aestivum	North
159099		T.aestivum	North
159101		T.aestivum	North
159103		T.aestivum	North
159104		T.aestivum	North
159106		T.aestivum	North
159107		T.aestivum	North
159108		T.aestivum	North
159109		T.aestivum	North
159110	√	T.aestivum	North
159111	√	T.aestivum	North
159112		T.aestivum	North

159113	√	T.aestivum	North
159114		T.aestivum	North
159115		T.aestivum	North
159116		T.aestivum	North
159117		T.aestivum	North
159118		T.aestivum	North
159119		T.aestivum	North
159121		T.aestivum	North
159122		T.aestivum	North
159123		T.aestivum	North
159124		T.aestivum	North
159125		T.aestivum	North
159126		T.aestivum	North
159127		T.aestivum	North
159128	√	T.aestivum	North
159129		T.aestivum	North
159130		T.aestivum	North
159131		T.aestivum	North
159132	√	T.aestivum	North
159133	√	T.aestivum	North
159134	√	T.aestivum	North
159136		T.aestivum	North
159137		T.aestivum	North
159138		T.aestivum	North
159139		T.aestivum	North
159140		T.aestivum	North
159142	√	T.aestivum	North
159143	√	T.aestivum	North
159144		T.aestivum	North
159145	√	T.aestivum	North
159146		T.aestivum	North
159147		T.aestivum	North
159148		T.aestivum	North
159149		T.aestivum	North
159150		T.aestivum	North
159151		T.aestivum	North
159152		T.aestivum	North
159153		T.aestivum	North
159154	√	T.aestivum	North
159155		T.aestivum	North
159156		T.aestivum	North

159158		T.aestivum	North
159159	√	T.aestivum	North
159161	√	T.aestivum	North
159163	√	T.aestivum	North
159164		T.aestivum	North
159165		T.aestivum	North
159166	√	T.aestivum	North
159167		T.aestivum	North
159168		T.aestivum	North
159169		T.aestivum	North
159170		T.aestivum	North
159171		T.aestivum	North
159172		T.aestivum	North
159173		T.aestivum	North
159174		T.aestivum	North
159176		T.aestivum	North
159177		T.aestivum	North
159178	√	T.aestivum	North
159179		T.aestivum	North
159180	√	T.aestivum	North
159181		T.aestivum	North
159182		T.aestivum	North
159183		T.aestivum	North
159184	√	T.aestivum	North
159185		T.aestivum	North
159186		T.aestivum	North
159187		T.aestivum	North
159188		T.aestivum	North
159190		T.aestivum	North
159191		T.aestivum	North
159192		T.aestivum	North
159193		T.aestivum	North
159194		T.aestivum	North
159195	√	T.aestivum	North
159196		T.aestivum	North
159197		T.aestivum	North
159198		T.aestivum	North
159199		T.aestivum	North
159200		T.aestivum	North
159203		T.aestivum	North
159204		T.aestivum	North

159206		T.aestivum	North
159207		T.aestivum	North
159208		T.aestivum	North
159209		T.aestivum	North
159210		T.aestivum	North
159212		T.aestivum	North
159213		T.aestivum	North
159214		T.aestivum	North
159215		T.aestivum	North
159217		T.aestivum	North
159219		T.aestivum	North
159220		T.aestivum	North
159221		T.aestivum	North
159222		T.aestivum	North
159227	√	T.aestivum	North
159228		T.aestivum	North
159231		T.aestivum	North
159232		T.aestivum	North
159233		T.aestivum	North
159234		T.aestivum	North
159235		T.aestivum	North
159236		T.aestivum	North
162001		T.aestivum	Central
162002		T.aestivum	Central
162003		T.aestivum	Central
162004		T.aestivum	Central
162006		T.aestivum	Central
162013		T.aestivum	Central
162014		T.aestivum	Central
162015		T.aestivum	Central
162016		T.aestivum	Central
162017		T.aestivum	Central
162018		T.aestivum	Central
162022		T.aestivum	Central
162023	√	T.aestivum	Central
162024		T.aestivum	Central
162025		T.aestivum	Central
162026	√	T.aestivum	Central
162027		T.aestivum	Central
162028	√	T.aestivum	Central
162030		T.aestivum	Central

162031	√	T.aestivum	Central
162032		T.aestivum	Central
162033		T.aestivum	Central
162034		T.aestivum	Central
162035	√	T.aestivum	Central
162036		T.aestivum	Central
162037		T.aestivum	Central
162040		T.aestivum	Central
162044		T.aestivum	Central
162045		T.aestivum	Central
162046		T.aestivum	Central
162048		T.aestivum	Central
162050		T.aestivum	Central
162051		T.aestivum	Central
162052		T.aestivum	Central
162053		T.aestivum	Central
162054		T.aestivum	Central
162055		T.aestivum	Central
162056		T.aestivum	Central
162057		T.aestivum	Central
162058		T.aestivum	Central
162059		T.aestivum	Central
162060		T.aestivum	Central
162061		T.aestivum	Central
162062		T.aestivum	Central
162063		T.aestivum	Central
162064		T.aestivum	Central
162065		T.aestivum	Central
162066		T.aestivum	Central
162068		T.aestivum	Central
162069		T.aestivum	Central
162070		T.aestivum	Central
162071	√	T.aestivum	Central
162073		T.aestivum	Central
162074		T.aestivum	Central
162076		T.aestivum	Central
162077		T.aestivum	Central
162078		T.aestivum	Central
162079		T.aestivum	Central
162080		T.aestivum	Central
162081		T.aestivum	Central

162083		T.aestivum	Central
162084		T.aestivum	Central
162085		T.aestivum	Central
162086		T.aestivum	Central
162088		T.aestivum	Central
162089		T.aestivum	Central
162091		T.aestivum	Central
162092	√	T.aestivum	Central
162093	√	T.aestivum	Central
162094		T.aestivum	Central
162095	√	T.aestivum	Central
162096		T.aestivum	Central
162097		T.aestivum	Central
162098		T.aestivum	Central
162099	√	T.aestivum	Central
162101		T.aestivum	Central
162102		T.aestivum	Central
162106		T.aestivum	Central
162107	√	T.aestivum	Central
162108		T.aestivum	Central
162109		T.aestivum	Central
162110		T.aestivum	Central
162111		T.aestivum	Central
162112		T.aestivum	Central
162113		T.aestivum	Central
162114	√	T.aestivum	Central
162115		T.aestivum	Central
162116		T.aestivum	Central
162117	√	T.aestivum	Central
162119		T.aestivum	Central
162120		T.aestivum	Central
162123		T.aestivum	Central
162124		T.aestivum	Central
162125		T.aestivum	Central
162129		T.aestivum	Central
162130		T.aestivum	Central
162131		T.aestivum	Central
162132		T.aestivum	Central
162133		T.aestivum	Central
162135		T.aestivum	Central
162136		T.aestivum	Central

162137		T.aestivum	Central
162138		T.aestivum	Central
162140	√	T.aestivum	Central
162141	√	T.aestivum	Central
162142		T.aestivum	Central
162145		T.aestivum	Central
162146		T.aestivum	Central
162147		T.aestivum	Central
162148	√	T.aestivum	Central
162149		T.aestivum	Central
162151		T.aestivum	Central
162152		T.aestivum	Central
162153	√	T.aestivum	Central
162154		T.aestivum	Central
162155		T.aestivum	Central
162156		T.aestivum	Central
162157		T.aestivum	Central
162159		T.aestivum	Central
162161	√	T.aestivum	Central
162162		T.aestivum	Central
162164		T.aestivum	Central
162165		T.aestivum	Central
162166		T.aestivum	Central
162167		T.aestivum	Central
162168		T.aestivum	Central
162169		T.aestivum	Central
162171		T.aestivum	Central
162172		T.aestivum	Central
162173		T.aestivum	Central
162174		T.aestivum	Central
162175		T.aestivum	Central
162176		T.aestivum	Central
162179		T.aestivum	Central
162181		T.aestivum	Central
162184		T.aestivum	Central
162187		T.aestivum	Central
162188		T.aestivum	Central
162189		T.aestivum	Central
162190	√	T.aestivum	Central
162191		T.aestivum	Central
162192		T.aestivum	Central

162193		T.aestivum	Central
162194		T.aestivum	Central
162196		T.aestivum	Central
162197		T.aestivum	Central
162198		T.aestivum	Central
162199	√	T.aestivum	Central
162200	√	T.aestivum	Central
162201		T.aestivum	Central
162202		T.aestivum	Central
162203		T.aestivum	Central
162205		T.aestivum	Central
162206		T.aestivum	Central
162207		T.aestivum	Central
162208		T.aestivum	Central
162210		T.aestivum	Central
162216	√	T.aestivum	Central
162221		T.aestivum	Central
162224		T.aestivum	Central
162225		T.aestivum	Central
162226		T.aestivum	Central
162227		T.aestivum	Central
162228	√	T.aestivum	Central
162229		T.aestivum	Central
162230		T.aestivum	Central
162231		T.aestivum	Central
162232		T.aestivum	Central
162233		T.aestivum	Central
162234	√	T.aestivum	Central
162235		T.aestivum	Central
162236		T.aestivum	Central
162237		T.aestivum	Central
162239		T.aestivum	Central
162240		T.aestivum	Central
162241		T.aestivum	Central
162242		T.aestivum	Central
162243		T.aestivum	Central
162244		T.aestivum	Central
162245	√	T.aestivum	Central
162247	√	T.aestivum	Central
162248	√	T.aestivum	Central
162249		T.aestivum	Central

162250		T.aestivum	Central
162251		T.aestivum	Central
162252		T.aestivum	Central
162253		T.aestivum	Central
162254		T.aestivum	Central
162255		T.aestivum	Central
162256		T.aestivum	Central
162257		T.aestivum	Central
162258	√	T.aestivum	Central
162260		T.aestivum	Central
162261	√	T.aestivum	Central
162262		T.aestivum	Central
162263		T.aestivum	Central
162264		T.aestivum	Central
162266		T.aestivum	Central
162267		T.aestivum	Central
162268		T.aestivum	Central
162269	√	T.aestivum	Central
162270		T.aestivum	Central
162272		T.aestivum	Central
162273		T.aestivum	Central
162274		T.aestivum	Central
162275		T.aestivum	Central
162276		T.aestivum	Central
162277		T.aestivum	Central
162278		T.aestivum	Central
162279		T.aestivum	Central
162280		T.aestivum	Central
162282		T.aestivum	Central
162283		T.aestivum	Central
162284		T.aestivum	Central
162285		T.aestivum	Central
162286	√	T.aestivum	Central
162287		T.aestivum	Central
162288		T.aestivum	Central
162289		T.aestivum	Central
162290		T.aestivum	Central
162291		T.aestivum	Central
162292		T.aestivum	Central
162293		T.aestivum	Central
162294		T.aestivum	Central

162295		T.aestivum	Central
162296		T.aestivum	Central
162297		T.aestivum	Central
162298		T.aestivum	Central
162299		T.aestivum	Central
162300	√	T.aestivum	Central
162301		T.aestivum	Central
162302		T.aestivum	Central
162303		T.aestivum	Central
162304		T.aestivum	Central
162305		T.aestivum	Central
162306		T.aestivum	Central
162307		T.aestivum	Central
162308		T.aestivum	Central
162309		T.aestivum	Central
162310		T.aestivum	Central
162311		T.aestivum	Central
162312		T.aestivum	Central
162313		T.aestivum	Central
162314	√	T.aestivum	Central
162315		T.aestivum	Central
162316		T.aestivum	Central
162317		T.aestivum	Central
162318		T.aestivum	Central
162319	√	T.aestivum	Central
162321		T.aestivum	Central
162322		T.aestivum	Central
162323		T.aestivum	Central
162324		T.aestivum	Central
162325		T.aestivum	Central
162326		T.aestivum	Central
162327	√	T.aestivum	Central
162328		T.aestivum	Central
162329		T.aestivum	Central
162330		T.aestivum	Central
162331		T.aestivum	Central
162332		T.aestivum	Central
162333		T.aestivum	Central
162335		T.aestivum	Central
162336	√	T.aestivum	Central
162337		T.aestivum	Central

162338		T.aestivum	Central
162339		T.aestivum	Central
162340		T.aestivum	Central
162341	√	T.aestivum	Central
162342		T.aestivum	Central
162343		T.aestivum	Central
162344		T.aestivum	Central
162345		T.aestivum	Central
162346		T.aestivum	Central
162347		T.aestivum	Central
162348		T.aestivum	Central
162349	√	T.aestivum	Central
162350		T.aestivum	Central
162351		T.aestivum	Central
162352		T.aestivum	Central
162353		T.aestivum	Central
162354		T.aestivum	Central
162355		T.aestivum	Central
162356		T.aestivum	Central
162358	√	T.aestivum	Central
162359		T.aestivum	Central
162361	√	T.aestivum	Central
162362		T.aestivum	Central
162363		T.aestivum	Central
162364		T.aestivum	Central
162365		T.aestivum	Central
162366		T.aestivum	Central
162368		T.aestivum	Central
162369		T.aestivum	Central
162370	√	T.aestivum	Central
162371		T.aestivum	Central
162372		T.aestivum	Central
162373		T.aestivum	Central
162374		T.aestivum	Central
162376		T.aestivum	Central
162377		T.aestivum	Central
162378		T.aestivum	Central
162379		T.aestivum	Central
162380		T.aestivum	Central
162381		T.aestivum	Central
162382		T.aestivum	Central

162384		T.aestivum	Central
162385	√	T.aestivum	Central
162386		T.aestivum	Central
162387		T.aestivum	Central
162388		T.aestivum	Central
162389		T.aestivum	Central
162390		T.aestivum	Central
162391	√	T.aestivum	Central
162392		T.aestivum	Central
162393		T.aestivum	Central
162395		T.aestivum	Central
162396		T.aestivum	Central
162398		T.aestivum	Central
162399		T.aestivum	Central
162400		T.aestivum	Central
162401		T.aestivum	Central
162405		T.aestivum	Central
162408		T.aestivum	Central
190845		T.aestivum	Central
190847		T.aestivum	Central
190849		T.aestivum	Central
190850		T.aestivum	Central
190851		T.aestivum	Central
190852		T.aestivum	Central
190853	√	T.aestivum	Central
190854	√	T.aestivum	Central
190855		T.aestivum	Central
190857		T.aestivum	Central
190858		T.aestivum	Central
190859		T.aestivum	Central
190860		T.aestivum	Central
190861		T.aestivum	Central
190862		T.aestivum	Central
190863		T.aestivum	Central
190864		T.aestivum	Central
190865		T.aestivum	Central
190866		T.aestivum	Central
190868		T.aestivum	Central
190869		T.aestivum	Central
190870		T.aestivum	Central
190871		T.aestivum	Central

190872		T.aestivum	Central
190873		T.aestivum	Central
190874		T.aestivum	Central
190875		T.aestivum	Central
190876		T.aestivum	Central
190877		T.aestivum	Central
190878		T.aestivum	Central
190880		T.aestivum	Central
190881		T.aestivum	Central
190882		T.aestivum	Central
190883	√	T.aestivum	Central
190884		T.aestivum	Central
190885		T.aestivum	Central
190886		T.aestivum	Central
190887		T.aestivum	Central
190888		T.aestivum	Central
190889		T.aestivum	Central
190890		T.aestivum	Central
190892	√	T.aestivum	Central
190893		T.aestivum	Central
190894		T.aestivum	Central
190895		T.aestivum	Central
190896		T.aestivum	Central
190897		T.aestivum	Central
190898	√	T.aestivum	Central
190899		T.aestivum	Central
190900	√	T.aestivum	Central
190901	√	T.aestivum	Central
190902	√	T.aestivum	Central
190903		T.aestivum	Central
190905		T.aestivum	Central
190906		T.aestivum	Central
190907		T.aestivum	Central
190908		T.aestivum	Central
190909		T.aestivum	Central
190910		T.aestivum	Central
190911		T.aestivum	Central
190912		T.aestivum	Central
190913		T.aestivum	Central
190914		T.aestivum	Central
190915		T.aestivum	Central

190916		T.aestivum	Central
190917		T.aestivum	Central
190918		T.aestivum	Central
190919		T.aestivum	Central
190920		T.aestivum	Central
190921		T.aestivum	Central
190922		T.aestivum	Central
190923		T.aestivum	Central
190924		T.aestivum	Central
190927		T.aestivum	Central
190928		T.aestivum	Central
190929		T.aestivum	Central
190930		T.aestivum	Central
190932		T.aestivum	Central
190933		T.aestivum	Central
190934		T.aestivum	Central
190935		T.aestivum	Central
190936		T.aestivum	Central
190937		T.aestivum	Central
190938		T.aestivum	Central
190939		T.aestivum	Central
190940		T.aestivum	Central
190942		T.aestivum	Central
190943		T.aestivum	Central
190944	√	T.aestivum	Central
190945		T.aestivum	Central
190946		T.aestivum	Central
190947		T.aestivum	Central
190948		T.aestivum	Central
190949		T.aestivum	Central
190950		T.aestivum	Central
190953	√	T.aestivum	Central
190969		T.aestivum	Central
190970		T.aestivum	Central
190971		T.aestivum	Central
190972		T.aestivum	Central
190973		T.aestivum	Central
190978		T.aestivum	Central
190979		T.aestivum	Central
190980		T.aestivum	Central
190981		T.aestivum	Central

190982		T.aestivum	Central
190983		T.aestivum	Central
190984		T.aestivum	Central
190985		T.aestivum	Central
190986		T.aestivum	Central
190987		T.aestivum	Central
190988		T.aestivum	Central
190989		T.aestivum	Central
190990		T.aestivum	Central
190991		T.aestivum	Central
190992		T.aestivum	Central
190993		T.aestivum	Central
190994		T.aestivum	Central
190995	√	T.aestivum	Central
190996		T.aestivum	Central
190997	√	T.aestivum	Central
190998		T.aestivum	Central
190999		T.aestivum	Central
191000		T.aestivum	Central
191001		T.aestivum	Central
191002		T.aestivum	Central
191003	√	T.aestivum	Central
191004		T.aestivum	Central
191005		T.aestivum	Central
191006		T.aestivum	Central
191007		T.aestivum	Central
191008		T.aestivum	Central
191009		T.aestivum	Central
191010	√	T.aestivum	Central
191011		T.aestivum	Central
191012		T.aestivum	Central
191013	√	T.aestivum	Central
191014		T.aestivum	Central
191015		T.aestivum	Central
191016		T.aestivum	Central
191017	√	T.aestivum	Central
191018		T.aestivum	Central
191019		T.aestivum	Central
191020		T.aestivum	Central
191021		T.aestivum	Central
191022		T.aestivum	Central

191023	√	T.aestivum	Central
191024		T.aestivum	Central
191025		T.aestivum	Central
191026	√	T.aestivum	Central
191027		T.aestivum	Central
191028		T.aestivum	Central
191029		T.aestivum	Central
191030		T.aestivum	Central
191031		T.aestivum	Central
191032	√	T.aestivum	Central
191033		T.aestivum	Central
191034		T.aestivum	Central
191035		T.aestivum	Central
191036	√	T.aestivum	Central
191037		T.aestivum	Central
191038		T.aestivum	Central
191039		T.aestivum	Central
191040		T.aestivum	Central
191041		T.aestivum	Central
191042		T.aestivum	Central
191043		T.aestivum	Central
191044	√	T.aestivum	Central
191045		T.aestivum	Central
191046		T.aestivum	Central
191047		T.aestivum	Central
191048	√	T.aestivum	Central
191049		T.aestivum	Central
191050		T.aestivum	Central
191051	√	T.aestivum	Central
191052		T.aestivum	Central
191053		T.aestivum	Central
191054		T.aestivum	Central
191055	√	T.aestivum	Central
191056		T.aestivum	Central
191057		T.aestivum	Central
191058		T.aestivum	Central
191059		T.aestivum	Central
191060		T.aestivum	Central
191061		T.aestivum	Central
191062		T.aestivum	Central
191063		T.aestivum	Central

191064		T.aestivum	Central
191065		T.aestivum	Central
191066		T.aestivum	Central
191067		T.aestivum	Central
191068	√	T.aestivum	Central
191069		T.aestivum	Central
191070		T.aestivum	Central
191071		T.aestivum	Central
191072		T.aestivum	Central
191073		T.aestivum	Central
191074	√	T.aestivum	Central
191075		T.aestivum	Central
191076		T.aestivum	Central
191077		T.aestivum	Central
191078		T.aestivum	Central
191079		T.aestivum	Central
191080		T.aestivum	Central
191081		T.aestivum	Central
191082		T.aestivum	Central
191083		T.aestivum	Central
191084		T.aestivum	Central
191085		T.aestivum	Central
191087		T.aestivum	Central
191088		T.aestivum	Central
191089		T.aestivum	Central
191090		T.aestivum	Central
191091		T.aestivum	Central
191092		T.aestivum	Central
191093		T.aestivum	Central
191094		T.aestivum	Central
191095		T.aestivum	Central
191096		T.aestivum	Central
191097		T.aestivum	Central
191098		T.aestivum	Central
191099		T.aestivum	Central
191100		T.aestivum	Central
191101		T.aestivum	Central
191102		T.aestivum	Central
191103		T.aestivum	Central
191104		T.aestivum	Central
191105		T.aestivum	Central

191106		T.aestivum	Central
191108		T.aestivum	Central
191110	√	T.aestivum	Central
191111		T.aestivum	Central
191112		T.aestivum	Central
191113		T.aestivum	Central
191114		T.aestivum	Central
191115		T.aestivum	Central
191116		T.aestivum	Central
191117		T.aestivum	Central
191118	√	T.aestivum	Central
191119		T.aestivum	Central
191120		T.aestivum	Central
191121		T.aestivum	Central
191122		T.aestivum	Central
191123	√	T.aestivum	Central
191124		T.aestivum	Central
191125		T.aestivum	Central
191126		T.aestivum	Central
191127		T.aestivum	Central
191128		T.aestivum	Central
191129		T.aestivum	Central
191130		T.aestivum	Central
191131		T.aestivum	Central
191132		T.aestivum	Central
191133		T.aestivum	Central
191134		T.aestivum	Central
191135	√	T.aestivum	Central
191136		T.aestivum	Central
191137		T.aestivum	Central
191138		T.aestivum	Central
191139		T.aestivum	Central
191140		T.aestivum	Central
191141		T.aestivum	Central
191142		T.aestivum	Central
191143		T.aestivum	Central
191144		T.aestivum	Central
191145		T.aestivum	Central
191146		T.aestivum	Central
191147		T.aestivum	Central
191150		T.aestivum	Central

191151	√	T.aestivum	Central
191152		T.aestivum	Central
191153		T.aestivum	Central
191154		T.aestivum	Central
191155		T.aestivum	Central
191156		T.aestivum	Central
191157	√	T.aestivum	Central
191158		T.aestivum	Central
191159		T.aestivum	Central
191160		T.aestivum	Central
191161	√	T.aestivum	Central
191162		T.aestivum	Central
191163	√	T.aestivum	Central
191164		T.aestivum	Central
191165		T.aestivum	Central
191166		T.aestivum	Central
191167		T.aestivum	Central
191168		T.aestivum	Central
191169		T.aestivum	Central
191170		T.aestivum	Central
191171	√	T.aestivum	Central
191172		T.aestivum	Central
191173		T.aestivum	Central
191174	√	T.aestivum	Central
191176	√	T.aestivum	Central
191177	√	T.aestivum	Central
191178		T.aestivum	Central
191180		T.aestivum	Central
191181		T.aestivum	Central
191182	√	T.aestivum	Central
191183		T.aestivum	Central
191184		T.aestivum	Central
191185		T.aestivum	Central
191186		T.aestivum	Central
191187	√	T.aestivum	Central
191188		T.aestivum	Central
191189		T.aestivum	Central
191190		T.aestivum	Central
191191		T.aestivum	Central
191192		T.aestivum	Central
191193		T.aestivum	Central

191194		T.aestivum	Central
191195		T.aestivum	Central
191196		T.aestivum	Central
191198		T.aestivum	Central
191199		T.aestivum	Central
191200		T.aestivum	Central
191201		T.aestivum	Central
191202	√	T.aestivum	Central
191203		T.aestivum	Central
191204	√	T.aestivum	Central
191205		T.aestivum	Central
191206		T.aestivum	Central
191207		T.aestivum	Central
191209		T.aestivum	Central
191210		T.aestivum	Central
191211		T.aestivum	Central
191212	√	T.aestivum	Central
191213		T.aestivum	Central
191214		T.aestivum	Central
191216		T.aestivum	Central
191217		T.aestivum	Central
191218		T.aestivum	Central
191219		T.aestivum	Central
191220		T.aestivum	Central
191221		T.aestivum	Central
191222		T.aestivum	Central
191223		T.aestivum	Central
191224		T.aestivum	Central
191225		T.aestivum	Central
191226		T.aestivum	Central
191227		T.aestivum	Central
191228		T.aestivum	Central
191229		T.aestivum	Central
191230		T.aestivum	Central
191231		T.aestivum	Central
191232		T.aestivum	Central
191233		T.aestivum	Central
191234		T.aestivum	Central
191235		T.aestivum	Central
191236		T.aestivum	Central
191237		T.aestivum	Central

191238		T.aestivum	Central
191239		T.aestivum	Central
191240		T.aestivum	Central
191241		T.aestivum	Central
191242		T.aestivum	Central
191243		T.aestivum	Central
191244	√	T.aestivum	Central
191245		T.aestivum	Central
191246		T.aestivum	Central
191247		T.aestivum	Central
191248	√	T.aestivum	Central
191249		T.aestivum	Central
191250		T.aestivum	Central
191251		T.aestivum	Central
191252	√	T.aestivum	Central
191253		T.aestivum	Central
191254		T.aestivum	Central
191255		T.aestivum	Central
191256	√	T.aestivum	Central
191257		T.aestivum	Central
191259		T.aestivum	Central
191262		T.aestivum	Central
191263		T.aestivum	Central
191264		T.aestivum	Central
191265		T.aestivum	Central
191266		T.aestivum	Central
191267		T.aestivum	Central
191268	√	T.aestivum	Central
191269		T.aestivum	Central
191271		T.aestivum	Central
191272		T.aestivum	Central
191273		T.aestivum	Central
191274	√	T.aestivum	Central
191275		T.aestivum	Central
191276		T.aestivum	Central
191277		T.aestivum	Central
191278		T.aestivum	Central
191279		T.aestivum	Central
191280		T.aestivum	Central
191281		T.aestivum	Central
191282		T.aestivum	Central

191283		T.aestivum	Central
191284		T.aestivum	Central
191285		T.aestivum	Central
191286		T.aestivum	Central
191287		T.aestivum	Central
191288		T.aestivum	Central
191289		T.aestivum	Central
191290		T.aestivum	Central
191291		T.aestivum	Central
191292		T.aestivum	Central
191293		T.aestivum	Central
191294		T.aestivum	Central
191295		T.aestivum	Central
191296		T.aestivum	Central
191297		t.aestivum	Central
191298		T.aestivum	Central
191299		T.aestivum	Central
191300	√	T.aestivum	Central
191301		T.aestivum	Central
191302		T.aestivum	Central
191303		T.aestivum	Central
191304		T.aestivum	Central
191305		T.aestivum	Central
191306	√	T.aestivum	Central
191307		T.aestivum	Central
191308		T.aestivum	Central
191309		T.aestivum	Central
191310		T.aestivum	Central
191311		T.aestivum	Central
191312		T.aestivum	Central
191313		T.aestivum	Central
191315		T.aestivum	Central
191316		T.aestivum	Central
191317		T.aestivum	Central
191318		T.aestivum	Central
191319		T.aestivum	Central
191320		T.aestivum	Central
191321		T.aestivum	Central
191322		T.aestivum	Central
191323		T.aestivum	Central
191324		T.aestivum	Central

191325		T.aestivum	Central
191326		T.aestivum	Central
191327		T.aestivum	Central
191328		T.aestivum	Central
191329	√	T.aestivum	Central
191332		T.aestivum	Central
191333		T.aestivum	Central
191334		T.aestivum	Central
191335		T.aestivum	Central
191336		T.aestivum	Central
191337	√	T.aestivum	Central
191338		T.aestivum	Central
191339		T.aestivum	Central
191340	√	T.aestivum	Central
191341		T.aestivum	Central
191344		T.aestivum	Central
191345		T.aestivum	Central
191346		T.aestivum	Central
191347		T.aestivum	Central
191348		T.aestivum	Central
191349		T.aestivum	Central
191350		T.aestivum	Central
191351		T.aestivum	Central
191352		T.aestivum	Central
191353		T.aestivum	Central
191354	√	T.aestivum	Central
191355		T.aestivum	Central
191356		T.aestivum	Central
191357		T.aestivum	Central
191359		T.aestivum	Central
191360		T.aestivum	Central
191361		T.aestivum	Central
191362		T.aestivum	Central
191363		T.aestivum	Central
191365		T.aestivum	Central
191367		T.aestivum	Central
191368		T.aestivum	Central
191369		T.aestivum	Central
191370		T.aestivum	Central
191371		T.aestivum	Central
191372		T.aestivum	Central

191373		T.aestivum	Central
191374		T.aestivum	Central
191375		T.aestivum	Central
191376		T.aestivum	Central
191377		T.aestivum	Central
191379		T.aestivum	Central
191380	√	T.aestivum	Central
191381		T.aestivum	Central
191382		T.aestivum	Central
191383		T.aestivum	Central
191384		T.aestivum	Central
191385		T.aestivum	Central
191386		T.aestivum	Central
191387		T.aestivum	Central
191389	√	T.aestivum	Central
191390		T.aestivum	Central
191391		T.aestivum	Central
191392		T.aestivum	Central
191393		T.aestivum	Central
191394		T.aestivum	Central
191395		T.aestivum	Central
191396	√	T.aestivum	Central
191397		T.aestivum	Central
191398		T.aestivum	Central
191399		T.aestivum	Central
191400		T.aestivum	Central
191401	√	T.aestivum	Central
191402		T.aestivum	Central
191403		T.aestivum	Central
191404		T.aestivum	Central
191405		T.aestivum	Central
191406	√	T.aestivum	Central
191407		T.aestivum	Central
191408		T.aestivum	Central
191409		T.aestivum	Central
191410	√	T.aestivum	Central
191411	√	T.aestivum	Central
191412		T.aestivum	Central
191415		T.aestivum	Central
191416		T.aestivum	Central
191417		T.aestivum	Central

191418	√	T.aestivum	Central
191419		T.aestivum	Central
191420		T.aestivum	Central
191421		T.aestivum	Central
191422	√	T.aestivum	Central
191423		T.aestivum	Central
191424		T.aestivum	Central
191426		T.aestivum	Central
191427		T.aestivum	Central
191428		T.aestivum	Central
191429		T.aestivum	Central
191430		T.aestivum	Central
191431		T.aestivum	Central
191432		T.aestivum	Central
191434		T.aestivum	Central
191435		T.aestivum	Central
191436		T.aestivum	Central
191437		T.aestivum	Central
191438		T.aestivum	Central
191439		T.aestivum	Central
191440		T.aestivum	Central
191441		T.aestivum	Central
191442		T.aestivum	Central
191443		T.aestivum	Central
191444		T.aestivum	Central
191445		T.aestivum	Central
191446		T.aestivum	Central
191447		T.aestivum	Central
191448		T.aestivum	Central
191449		T.aestivum	Central
191450		T.aestivum	Central
191451		T.aestivum	Central
191452		T.aestivum	Central
191453		T.aestivum	Central
191454		T.aestivum	Central
191455		T.aestivum	Central
191456		T.aestivum	Central
191457		T.aestivum	Central
191458		T.aestivum	Central
191459		T.aestivum	Central
191460		T.aestivum	Central

191461	√	T.aestivum	Central
191462		T.aestivum	Central
191463		T.aestivum	Central
191464		T.aestivum	Central
191465		T.aestivum	Central
191466		T.aestivum	Central
191467		T.aestivum	Central
191468		T.aestivum	Central
191469		T.aestivum	Central
191470		T.aestivum	Central
191471		T.aestivum	Central
191472		T.aestivum	Central
191473		T.aestivum	Central
191474		T.aestivum	Central
191475		T.aestivum	Central
191476		T.aestivum	Central
191477	√	T.aestivum	Central
191478		T.aestivum	Central
191479		T.aestivum	Central
191480		T.aestivum	Central
191481		T.aestivum	Central
191482		T.aestivum	Central
191483		T.aestivum	Central
191484		T.aestivum	Central
191485		T.aestivum	Central
191489		T.aestivum	Central
191490		T.aestivum	Central
191491	√	T.aestivum	Central
191492		T.aestivum	Central
191493		T.aestivum	Central
191494		T.aestivum	Central
191495		T.aestivum	Central
191496		T.aestivum	Central
191497		T.aestivum	Central
191498		T.aestivum	Central
191499		T.aestivum	Central
191500		T.aestivum	Central
191501		T.aestivum	Central
191502		T.aestivum	Central
191503		T.aestivum	Central
191504		T.aestivum	Central

191505		T.aestivum	Central
191506		T.aestivum	Central
191507	√	T.aestivum	Central
191508		T.aestivum	Central
191509		T.aestivum	Central
191510		T.aestivum	Central
191511	√	T.aestivum	Central
191512	√	T.aestivum	Central
191513		T.aestivum	Central
191514		T.aestivum	Central
191515	√	T.aestivum	Central
191517		T.aestivum	Central
191518		T.aestivum	Central
191519	√	T.aestivum	Central
191520	√	T.aestivum	Central
191521		T.aestivum	Central
191523		T.aestivum	Central
191524		T.aestivum	Central
191525		T.aestivum	Central
191526		T.aestivum	Central
191527	√	T.aestivum	Central
191528		T.aestivum	Central
191530		T.aestivum	Central
191531		T.aestivum	Central
191532	√	T.aestivum	Central
191533	√	T.aestivum	Central
191534		T.aestivum	Central
191536		T.aestivum	Central
191538		T.aestivum	Central
191539		T.aestivum	Central
191540		T.aestivum	Central
191541		T.aestivum	Central
191542		T.aestivum	Central
191543		T.aestivum	Central
191544		T.aestivum	Central
191545		T.aestivum	Central
191546		T.aestivum	Central
191547		T.aestivum	Central
191548	√	T.aestivum	Central
191549		T.aestivum	Central
191550		T.aestivum	Central

191551		T.aestivum	Central
191552		T.aestivum	Central
191553		T.aestivum	Central
191554		T.aestivum	Central
191556		T.aestivum	Central
191557		T.aestivum	Central
191558		T.aestivum	Central
191559		T.aestivum	Central
191560		T.aestivum	Central
191561		T.aestivum	Central
191562		T.aestivum	Central
191563		T.aestivum	Central
191564		T.aestivum	Central
191565		T.aestivum	Central
191566		T.aestivum	Central
191567		T.aestivum	Central
191569		T.aestivum	Central
191570		T.aestivum	Central
191572		T.aestivum	Central
191573		T.aestivum	Central
191574	√	T.aestivum	Central
191575		T.aestivum	Central
191576		T.aestivum	Central
191577	√	T.aestivum	Central
191578		T.aestivum	Central
191579		T.aestivum	Central
191580		T.aestivum	Central
191581		T.aestivum	Central
191582		T.aestivum	Central
191583		T.aestivum	Central
191584	√	T.aestivum	Central
191585	√	T.aestivum	Central
191586		T.aestivum	Central
191587		T.aestivum	Central
191589		T.aestivum	Central
191590		T.aestivum	Central
191591	√	T.aestivum	Central
191593		T.aestivum	Central
191594	√	T.aestivum	Central
191595		T.aestivum	Central
191596		T.aestivum	Central

191597		T.aestivum	Central
191598		T.aestivum	Central
191599		T.aestivum	Central
191600		T.aestivum	Central
191601		T.aestivum	Central
191602		T.aestivum	Central
191603		T.aestivum	Central
191604		T.aestivum	Central
191605		T.aestivum	Central
191606		T.aestivum	Central
191607	√	T.aestivum	Central
191609		T.aestivum	Central
191610		T.aestivum	Central
191611		T.aestivum	Central
191612		T.aestivum	Central
191613		T.aestivum	Central
191614		T.aestivum	Central
191615		T.aestivum	Central
191616		T.aestivum	Central
191617	√	T.aestivum	Central
191618		T.aestivum	Central
191619		T.aestivum	Central
191620		T.aestivum	Central
191621		T.aestivum	Central
191622		T.aestivum	Central
191623		T.aestivum	Central
191625	√	T.aestivum	Central
191626		T.aestivum	Central
191627		T.aestivum	Central
191628		T.aestivum	Central
191629		T.aestivum	Central
191630		T.aestivum	Central
191632		T.aestivum	Central
191633		T.aestivum	Central
191634		T.aestivum	Central
191635	√	T.aestivum	Central
191636		T.aestivum	Central
191638		T.aestivum	Central
191639		T.aestivum	Central
191640		T.aestivum	Central
191641	√	T.aestivum	Central

191642		T.aestivum	Central
191643		T.aestivum	Central
191645	√	T.aestivum	Central
191646		T.aestivum	Central
191648		T.aestivum	Central
191649		T.aestivum	Central
191650		T.aestivum	Central
191651		T.aestivum	Central
191652		T.aestivum	Central
191653		T.aestivum	Central
191654	√	T.aestivum	Central
191655		T.aestivum	Central
191657		T.aestivum	Central
191658	√	T.aestivum	Central
191659		T.aestivum	Central
191660		T.aestivum	Central
191661		T.aestivum	Central
191662		T.aestivum	Central
191663		T.aestivum	Central
191664	√	T.aestivum	Central
191665		T.aestivum	Central
191666		T.aestivum	Central
191667		T.aestivum	Central
191668		T.aestivum	Central
191669		T.aestivum	Central
191670	√	T.aestivum	Central
191671		T.aestivum	Central
191672		T.aestivum	Central
191673		T.aestivum	Central
191674		T.aestivum	Central
191675		T.aestivum	Central
191676		T.aestivum	Central
191677	√	T.aestivum	Central
191678		T.aestivum	Central
191679		T.aestivum	Central
191680		T.aestivum	Central
191681	√	T.aestivum	Central
191682		T.aestivum	Central
191683		T.aestivum	Central
191684	√	T.aestivum	Central
191685		t.aestivum	Central

191687		T.aestivum	Central
191688	√	T.aestivum	Central
191691		T.aestivum	Central
191692		T.aestivum	Central
191693	√	T.aestivum	Central
191694		T.aestivum	Central
191695	√	T.aestivum	Central
191696		T.aestivum	Central
191697		T.aestivum	Central
191698	√	T.aestivum	Central
191699		T.aestivum	Central
191700		T.aestivum	Central
191702		T.aestivum	Central
191703		T.aestivum	Central
191704		T.aestivum	Central
191706		T.aestivum	Central
191707	√	T.aestivum	Central
191708		T.aestivum	Central
191709		T.aestivum	Central
191710		T.aestivum	Central
191711		T.aestivum	Central
191712		T.aestivum	Central
191713	√	T.aestivum	Central
191714		T.aestivum	Central
191715		T.aestivum	Central
191716	√	T.aestivum	Central
191717		T.aestivum	Central
191718	√	T.aestivum	Central
191719	√	t.aestivum	Central
191720		T.aestivum	Central
191721	√	T.aestivum	Central
191722		T.aestivum	Central
191723	√	T.aestivum	Central
191724		T.aestivum	Central
191726		T.aestivum	Central
191728		T.aestivum	Central
191729		T.aestivum	Central
191730		T.aestivum	Central
191731		T.aestivum	Central
191734		T.aestivum	Central
191735		T.aestivum	Central

191736		T.aestivum	Central
191739		T.aestivum	Central
191740		T.aestivum	Central
191741		T.aestivum	Central
191742	√	T.aestivum	Central
191743		T.aestivum	Central
191744		T.aestivum	Central
191745		T.aestivum	Central
191746		T.aestivum	Central
191747	√	T.aestivum	Central
191748		T.aestivum	Central
191749		T.aestivum	Central
191750		T.aestivum	Central
191751		T.aestivum	Central
191752		T.aestivum	Central
191753		T.aestivum	Central
191754		T.aestivum	Central
191755		T.aestivum	Central
191756		T.aestivum	Central
191757		T.aestivum	Central
191758		T.aestivum	Central
191759		T.aestivum	Central
191760		T.aestivum	Central
191761		T.aestivum	Central
191762		T.aestivum	Central
191763		T.aestivum	Central
191764		T.aestivum	Central
191765	√	T.aestivum	Central
191766		T.aestivum	Central
191767		T.aestivum	Central
191768		T.aestivum	Central
191769	√	T.aestivum	Central
191770		T.aestivum	Central
191771		T.aestivum	Central
191773		T.aestivum	Central
191774		T.aestivum	Central
191775		T.aestivum	Central
191776		T.aestivum	Central
191777	√	T.aestivum	Central
191778		T.aestivum	Central
191779		T.aestivum	Central

191782		T.aestivum	Central
191783		T.aestivum	Central
191784		T.aestivum	Central
191785	√	T.aestivum	Central
191787		T.aestivum	Central
191788		T.aestivum	Central
191789	√	T.aestivum	Central
191790		T.aestivum	Central
191792		T.aestivum	Central
191793		T.aestivum	Central
191794		T.aestivum	Central
191797		T.aestivum	Central
191798		T.aestivum	Central
191799		T.aestivum	Central
191800		T.aestivum	Central
191801		T.aestivum	Central
191802		T.aestivum	Central
191805		T.aestivum	Central
191806		T.aestivum	Central
191807		T.aestivum	Central
191808	√	T.aestivum	Central
191809		T.aestivum	Central
191811	√	T.aestivum	Central
191815		T.aestivum	Central
191816		T.aestivum	Central
191817		T.aestivum	Central
191818		T.aestivum	Central
191819		T.aestivum	Central
191821		T.aestivum	Central
191822		T.aestivum	Central
191823		T.aestivum	Central
191827		T.aestivum	Central
191830		T.aestivum	Central
191831		T.aestivum	Central
191835		T.aestivum	Central
191838		T.aestivum	Central
191839	√	T.aestivum	Central
191841		T.aestivum	Central
191842		T.aestivum	Central
191843		T.aestivum	Central
191845		T.aestivum	Central

191847		T.aestivum	Central
191851		T.aestivum	Central
191855		T.aestivum	Central
191856		T.aestivum	Central
191857		T.aestivum	Central
191858	√	T.aestivum	Central
191859		T.aestivum	Central
191860		T.aestivum	Central
191864		T.aestivum	Central
191867	√	T.aestivum	Central
191870	√	T.aestivum	Central
191871		T.aestivum	Central
191872		T.aestivum	Central
191873		T.aestivum	Central
191874		T.aestivum	Central
191875		T.aestivum	Central
191876		T.aestivum	Central
191877		T.aestivum	Central
191878		T.aestivum	Central
191879		T.aestivum	Central
191880		T.aestivum	Central
191881		T.aestivum	Central
191884		T.aestivum	Central
191885		T.aestivum	Central
191886		T.aestivum	Central
191888		T.aestivum	Central
191889		T.aestivum	Central
191890		T.aestivum	Central
191894		T.aestivum	Central
191895		T.aestivum	Central
191896		T.aestivum	Central
191897		T.aestivum	Central
191898		T.aestivum	Central
191900		T.aestivum	Central
191901		T.aestivum	Central
191902		T.aestivum	Central
191904	√	T.aestivum	Central
191905		T.aestivum	Central
191906		T.aestivum	Central
191907		T.aestivum	Central
191908		T.aestivum	Central

191909		T.aestivum	Central
191911		T.aestivum	Central
191912		T.aestivum	Central
191913		T.aestivum	Central
191914	√	T.aestivum	Central
191916		T.aestivum	Central
191917		T.aestivum	Central
191919		T.aestivum	Central
191920		T.aestivum	Central
191921		T.aestivum	Central
191923		T.aestivum	Central
191924	√	T.aestivum	Central
191925		T.aestivum	Central
191926		T.aestivum	Central
191927		T.aestivum	Central
191928		T.aestivum	Central
191929		T.aestivum	Central
191930	√	T.aestivum	Central
191933		T.aestivum	Central
191934	√	T.aestivum	Central
191935		T.aestivum	Central
191936		T.aestivum	Central
191939		T.aestivum	Central
191941		T.aestivum	Central
191943		T.aestivum	Central
191945		T.aestivum	Central
191946		T.aestivum	Central
191947		T.aestivum	Central
191948		T.aestivum	Central
191949	√	T.aestivum	Central
191950		T.aestivum	Central
191952		T.aestivum	Central
191953		T.aestivum	Central
191954		T.aestivum	Central
191956		T.aestivum	Central
191957		T.aestivum	Central
191958		T.aestivum	Central
191959		T.aestivum	Central
191960		T.aestivum	Central
191961		T.aestivum	Central
191962		T.aestivum	Central

191963		T.aestivum	Central
191964		T.aestivum	Central
191966		T.aestivum	Central
191967	√	T.aestivum	Central
191968		T.aestivum	Central
191970		T.aestivum	Central
191971	√	T.aestivum	Central
191972		T.aestivum	Central
191973		T.aestivum	Central
191974		T.aestivum	Central
191975		T.aestivum	Central
191976		T.aestivum	Central
191978		T.aestivum	Central
191979		T.aestivum	Central
191982		T.aestivum	Central
191983		T.aestivum	Central
191985	√	T.aestivum	Central
191987		T.aestivum	Central
191988	√	T.aestivum	Central
191989		T.aestivum	Central
191990		T.aestivum	Central
191991		T.aestivum	Central
191992		T.aestivum	Central
191993		T.aestivum	Central
191994		T.aestivum	Central
191996		T.aestivum	Central
191997		T.aestivum	Central
191999		T.aestivum	Central
192000		T.aestivum	Central
192001		T.aestivum	Central
192002		T.aestivum	Central
192003		T.aestivum	Central
192004		T.aestivum	Central
192005	√	T.aestivum	Central
192006		T.aestivum	Central
192007	√	T.aestivum	Central
192008		T.aestivum	Central
192009		T.aestivum	Central
192010		T.aestivum	Central
192011		T.aestivum	Central
192012		T.aestivum	Central

192013		T.aestivum	Central
192014		T.aestivum	Central
192015		T.aestivum	Central
192016	√	T.aestivum	Central
192017		T.aestivum	Central
192018		T.aestivum	Central
192020		T.aestivum	Central
192024		T.aestivum	Central
192026		T.aestivum	Central
192027	√	T.aestivum	Central
192028		T.aestivum	Central
192030		T.aestivum	Central
192031		T.aestivum	Central
192032		T.aestivum	Central
192034		T.aestivum	Central
192035		T.aestivum	Central
192036		T.aestivum	Central
192037		T.aestivum	Central
192039		T.aestivum	Central
192040		T.aestivum	Central
192042		T.aestivum	Central
192043		T.aestivum	Central
192044		T.aestivum	Central
192045		T.aestivum	Central
192046		T.aestivum	Central
192047		T.aestivum	Central
192049		T.aestivum	Central
192050		T.aestivum	Central
192051		T.aestivum	Central
192052		T.aestivum	Central
192053		T.aestivum	Central
192054		T.aestivum	Central
192057		T.aestivum	Central
192059		T.aestivum	Central
192060	√	T.aestivum	Central
192061	√	T.aestivum	Central
192062		T.aestivum	Central
192063		T.aestivum	Central
192064		T.aestivum	Central
192065		T.aestivum	Central
192066		T.aestivum	Central

192067		T.aestivum	Central
192068		T.aestivum	Central
192069		T.aestivum	Central
192072	√	T.aestivum	Central
192073	√	T.aestivum	Central
192075		T.aestivum	Central
192076		T.aestivum	Central
192078		T.aestivum	Central
192079		T.aestivum	Central
192080		T.aestivum	Central
192082		T.aestivum	Central
192083		T.aestivum	Central
192084		T.aestivum	Central
192085		T.aestivum	Central
192086		T.aestivum	Central
192087		T.aestivum	Central
192088		T.aestivum	Central
192089		T.aestivum	Central
192090		T.aestivum	Central
192096		T.aestivum	Central
192097	√	T.aestivum	Central
192098		T.aestivum	Central
192102		T.aestivum	Central
192103		T.aestivum	Central
192104		T.aestivum	Central
192106		T.aestivum	Central
192107		T.aestivum	Central
192108		T.aestivum	Central
192109		T.aestivum	Central
192110		T.aestivum	Central
192111		T.aestivum	Central
192112		T.aestivum	Central
192113	√	T.aestivum	Central
192114	√	T.aestivum	Central
192115		T.aestivum	Central
192116		T.aestivum	Central
192118		T.aestivum	Central
192121		T.aestivum	Central
192122		T.aestivum	Central
192123		T.aestivum	Central
192124		T.aestivum	Central

192125	√	T.aestivum	Central
192126		T.aestivum	Central
192128		T.aestivum	Central
192129		T.aestivum	Central
192130		T.aestivum	Central
192132		T.aestivum	Central
192137		T.aestivum	Central
192139		T.aestivum	Central
192140		T.aestivum	Central
192142		T.aestivum	Central
192144		T.aestivum	Central
192145	√	T.aestivum	Central
192146		T.aestivum	Central
192149		T.aestivum	Central
192151		T.aestivum	Central
192152		T.aestivum	Central
192153		T.aestivum	Central
192154		T.aestivum	Central
192155		T.aestivum	Central
192156		T.aestivum	Central
192157		T.aestivum	Central
192158		T.aestivum	Central
192159		T.aestivum	Central
192160		T.aestivum	Central
192161		T.aestivum	Central
192162	√	T.aestivum	Central
192163		T.aestivum	Central
192164		T.aestivum	Central
192165		T.aestivum	Central
192166		T.aestivum	Central
192167		T.aestivum	Central
192168		T.aestivum	Central
192169		T.aestivum	Central
192170		T.aestivum	Central
192172	√	T.aestivum	Central
192173		T.aestivum	Central
192174		T.aestivum	Central
192175		T.aestivum	Central
192176		T.aestivum	Central
192177		T.aestivum	Central
192182		T.aestivum	Central

192183		T.aestivum	Central
192184	√	T.aestivum	Central
192186		T.aestivum	Central
192187		T.aestivum	Central
192193		T.aestivum	Central
192194		T.aestivum	Central
192196		T.aestivum	Central
192207		T.aestivum	Central
192215		T.aestivum	Central
192218		T.aestivum	Central
192219		T.aestivum	Central
192237	√	T.aestivum	Central
192243		T.aestivum	Central
192246		T.aestivum	Central
192252		T.aestivum	Central
192255		T.aestivum	Central
192262		T.aestivum	Central
192273		T.aestivum	Central
192276		T.aestivum	Central
192277		T.aestivum	Central
192279	√	T.aestivum	Central
192280		T.aestivum	Central
192281		T.aestivum	Central
192282		T.aestivum	Central
192283		T.aestivum	Central
192288		T.aestivum	Central
192289		T.aestivum	Central
192292		T.aestivum	Central
192293		T.aestivum	Central
192294		T.aestivum	Central
192296		T.aestivum	Central
192297		T.aestivum	Central
192298		T.aestivum	Central
192299	√	T.aestivum	Central
192300		T.aestivum	Central
192301		T.aestivum	Central
192302		T.aestivum	Central
192303		T.aestivum	Central
192304		T.aestivum	Central
192311		T.aestivum	Central
192312		T.aestivum	Central

192313	√	T.aestivum	Central
192314		T.aestivum	Central
192317		T.aestivum	Central
192318		T.aestivum	Central
192319		T.aestivum	Central
192322		T.aestivum	Central
192323		T.aestivum	Central
192324		T.aestivum	Central
192325		T.aestivum	Central
192326		T.aestivum	Central
192327		T.aestivum	Central
192328	√	T.aestivum	Central
192329		T.aestivum	Central
192330		T.aestivum	Central
192331		T.aestivum	Central
192332		T.aestivum	Central
192333		T.aestivum	Central
192335		T.aestivum	Central
192336		T.aestivum	Central
192337		T.aestivum	Central
192338		T.aestivum	Central
192339		T.aestivum	Central
192342		T.aestivum	Central
192343		T.aestivum	Central
192348		T.aestivum	Central
192349		T.aestivum	Central
192350		T.aestivum	Central
192351		T.aestivum	Central
192352		T.aestivum	Central
192353		T.aestivum	Central
192354		T.aestivum	Central
192355		T.aestivum	Central
192356		T.aestivum	Central
192357		T.aestivum	Central
192358		T.aestivum	Central
192359		T.aestivum	Central
192360		T.aestivum	Central
192361		T.aestivum	Central
192362		T.aestivum	Central
192363		T.aestivum	Central
192364		T.aestivum	Central

192365		T.aestivum	Central
192366		T.aestivum	Central
192370	√	T.aestivum	Central
192371		T.aestivum	Central
192372		T.aestivum	Central
192373		T.aestivum	Central
192374		T.aestivum	Central
192375		T.aestivum	Central
192376		T.aestivum	Central
192377		T.aestivum	Central
192383		T.aestivum	Central
192387		T.aestivum	Central
192399		T.aestivum	Central
192404		T.aestivum	Central
193108		t.aestivum	Central
193145		T.aestivum	Central
193186		T.aestivum	Central
193187		T.aestivum	Central
193188		T.aestivum	Central
193189		T.aestivum	Central
193191		T.aestivum	Central
193192		T.aestivum	Central
193193		T.aestivum	Central
193194		T.aestivum	Central
193195		T.aestivum	Central
193196		T.aestivum	Central
193197	√	T.aestivum	Central
193198		T.aestivum	Central
193199		T.aestivum	Central
193200		T.aestivum	Central
193201	√	T.aestivum	Central
193202		T.aestivum	Central
193203		T.aestivum	Central
193204		T.aestivum	Central
193205		T.aestivum	Central
193206		T.aestivum	Central
193207		T.aestivum	Central
193208		T.aestivum	Central
193209		T.aestivum	Central
193210		T.aestivum	Central
193211	√	T.aestivum	Central

193212		T.aestivum	Central
193213		T.aestivum	Central
193214		T.aestivum	Central
193215		T.aestivum	Central
193216		T.aestivum	Central
193217		T.aestivum	Central
193218		T.aestivum	Central
193219		T.aestivum	Central
193220		T.aestivum	Central
193221		T.aestivum	Central
193222		T.aestivum	Central
193223		T.aestivum	Central
193224		T.aestivum	Central
193225	√	T.aestivum	Central
193226		T.aestivum	Central
193227		T.aestivum	Central
193228		T.aestivum	Central
193230		T.aestivum	Central
193231		T.aestivum	Central
193232		T.aestivum	Central
193233		T.aestivum	Central
193234		T.aestivum	Central
193235	√	T.aestivum	Central
193236		T.aestivum	Central
193237		T.aestivum	Central
193238		T.aestivum	Central
193239		T.aestivum	Central
193240		T.aestivum	Central
193241		T.aestivum	Central
193242		T.aestivum	Central
193243		T.aestivum	Central
193244		T.aestivum	Central
193245	√	T.aestivum	Central
193246		T.aestivum	Central
193247		T.aestivum	Central
193248		T.aestivum	Central
193249	√	T.aestivum	Central
193250		T.aestivum	Central
193251		T.aestivum	Central
193252		T.aestivum	Central
193253		T.aestivum	Central

193254		T.aestivum	Central
193255		T.aestivum	Central
193256		T.aestivum	Central
193257		T.aestivum	Central
193258	√	T.aestivum	Central
193259		T.aestivum	Central
193260		T.aestivum	Central
193261		T.aestivum	Central
193262		T.aestivum	Central
193263		T.aestivum	Central
193264		T.aestivum	Central
193265		T.aestivum	Central
193266		T.aestivum	Central
193267		T.aestivum	Central
193268	√	T.aestivum	Central
193269		T.aestivum	Central
193270		T.aestivum	Central
193271	√	T.aestivum	Central
193272		T.aestivum	Central
193273		T.aestivum	Central
193274		T.aestivum	Central
193276		T.aestivum	Central
193277		T.aestivum	Central
193278		T.aestivum	Central
193279	√	T.aestivum	Central
193280		T.aestivum	Central
193281		T.aestivum	Central
193282		T.aestivum	Central
193283		T.aestivum	Central
193284	√	T.aestivum	Central
193285		T.aestivum	Central
193286		T.aestivum	Central
193287		T.aestivum	Central
193288		T.aestivum	Central
193289		T.aestivum	Central
193290		T.aestivum	Central
193292		T.aestivum	Central
193293		T.aestivum	Central
193294	√	T.aestivum	Central
193295		T.aestivum	Central
193296		T.aestivum	Central

193297		T.aestivum	Central
193298		T.aestivum	Central
193299		T.aestivum	Central
193300		T.aestivum	Central
193301	√	T.aestivum	Central
193302	√	T.aestivum	Central
193303		T.aestivum	Central
193304	√	T.aestivum	Central
193305		T.aestivum	Central
193306		T.aestivum	Central
193307		T.aestivum	Central
193309		T.aestivum	Central
193310		T.aestivum	Central
193311	√	T.aestivum	Central
193312		T.aestivum	Central
193313		T.aestivum	Central
193314		T.aestivum	Central
193315	√	t.aestivum	Central
193318		T.aestivum	Central
193320		T.aestivum	Central
193321	√	T.aestivum	Central
193363	√	T.aestivum	Central
193364		T.aestivum	Central
193365		T.aestivum	Central
193366		T.aestivum	Central
193368		T.aestivum	Central
193369		T.aestivum	Central
193370		T.aestivum	Central
193371		T.aestivum	Central
193372		T.aestivum	Central
193373		T.aestivum	Central
193374		T.aestivum	Central
193375		T.aestivum	Central
193376	√	T.aestivum	Central
193377	√	T.aestivum	Central
193378		T.aestivum	Central
193379		T.aestivum	Central
193380		T.aestivum	Central
193381		T.aestivum	Central
193382		T.aestivum	Central
193383	√	T.aestivum	Central

193384		T.aestivum	Central
193385		T.aestivum	Central
193386		T.aestivum	Central
193387		T.aestivum	Central
193388		T.aestivum	Central
193389		T.aestivum	Central
193390		T.aestivum	Central
193391		T.aestivum	Central
193392		T.aestivum	Central
193393	√	T.aestivum	Central
193394		T.aestivum	Central
193395		T.aestivum	Central
193396		T.aestivum	Central
193397		T.aestivum	Central
193398		T.aestivum	Central
193399	√	T.aestivum	Central
193400		T.aestivum	Central
193401		T.aestivum	Central
193402		T.aestivum	Central
193403		T.aestivum	Central
193404	√	T.aestivum	Central
193405		T.aestivum	Central
193406		T.aestivum	Central
193407		T.aestivum	Central
193408		T.aestivum	Central
193409		T.aestivum	Central
193411		T.aestivum	Central
193412		T.aestivum	Central
193414		T.aestivum	Central
193415		T.aestivum	Central
193416		T.aestivum	Central
193417		T.aestivum	Central
193418		T.aestivum	Central
193419		T.aestivum	Central
193420		T.aestivum	Central
193421		T.aestivum	Central
193422		T.aestivum	Central
193423		T.aestivum	Central
193424		T.aestivum	Central
193425		T.aestivum	Central
193426		T.aestivum	Central

193427	√	T.aestivum	Central
193428		T.aestivum	Central
193429		T.aestivum	Central
193430		T.aestivum	Central
193431	√	T.aestivum	Central
193432		T.aestivum	Central
193433		T.aestivum	Central
193435		T.aestivum	Central
193436		T.aestivum	Central
193437		T.aestivum	Central
193438		T.aestivum	Central
193439	√	T.aestivum	Central
193441		T.aestivum	Central
193442		T.aestivum	Central
193443		T.aestivum	Central
193444	√	T.aestivum	Central
193445		T.aestivum	Central
193446		T.aestivum	Central
193447		T.aestivum	Central
193448		T.aestivum	Central
193449		T.aestivum	Central
193450		T.aestivum	Central
193451		T.aestivum	Central
193452		T.aestivum	Central
193453		T.aestivum	Central
193454		T.aestivum	Central
193455		T.aestivum	Central
193456		T.aestivum	Central
193457		T.aestivum	Central
193458		T.aestivum	Central
193459		T.aestivum	Central
193460		T.aestivum	Central
193461		T.aestivum	Central
193462		T.aestivum	Central
193463		T.aestivum	Central
193464		T.aestivum	Central
193465		T.aestivum	Central
193466		T.aestivum	Central
193467		T.aestivum	Central
193468		T.aestivum	Central
193469		T.aestivum	Central

193470		T.aestivum	Central
193471		T.aestivum	Central
193472		T.aestivum	Central
193473		T.aestivum	Central
193474		T.aestivum	Central
193475		T.aestivum	Central
193476		T.aestivum	Central
193477		T.aestivum	Central
193478		T.aestivum	Central
193479		T.aestivum	Central
193480		T.aestivum	Central
193481	√	T.aestivum	Central
193482		T.aestivum	Central
193483		T.aestivum	Central
193484		T.aestivum	Central
193485		T.aestivum	Central
193486		T.aestivum	Central
193487	√	T.aestivum	Central
193488		T.aestivum	Central
193491	√	T.aestivum	Central
193492		T.aestivum	Central
193493	√	T.aestivum	Central
193494		T.aestivum	Central
193495	√	T.aestivum	Central
193496		T.aestivum	Central
193497		T.aestivum	Central
193498		T.aestivum	Central
193499		T.aestivum	Central
193500		T.aestivum	Central
193501		T.aestivum	Central
193502	√	T.aestivum	Central
193503		T.aestivum	Central
193516		T.aestivum	Central
193517		T.aestivum	Central
193518		T.aestivum	Central
193519		T.aestivum	Central
193520		T.aestivum	Central
193521		T.aestivum	Central
193522		T.aestivum	Central
193523		T.aestivum	Central
193524		T.aestivum	Central

193525		T.aestivum	Central
193526		T.aestivum	Central
193527		T.aestivum	Central
193528		T.aestivum	Central
193529		T.aestivum	Central
193530		T.aestivum	Central
193531	√	T.aestivum	Central
193532		T.aestivum	Central
193533		T.aestivum	Central
193534		T.aestivum	Central
193535		T.aestivum	Central
193536		T.aestivum	Central
193537		T.aestivum	Central
193538		T.aestivum	Central
193539		T.aestivum	Central
193540		T.aestivum	Central
193541		T.aestivum	Central
193542	√	T.aestivum	Central
193543		T.aestivum	Central
193544		T.aestivum	Central
193545		T.aestivum	Central
193546		T.aestivum	Central
193547	√	T.aestivum	Central
193548		T.aestivum	Central
193549		T.aestivum	Central
193550		T.aestivum	Central
193552		T.aestivum	Central
193553		T.aestivum	Central
193554		T.aestivum	Central
193555		T.aestivum	Central
193556		T.aestivum	Central
193557	√	T.aestivum	Central
193558	√	T.aestivum	Central
193559		T.aestivum	Central
193560		T.aestivum	Central
193561		T.aestivum	Central
193562		T.aestivum	Central
193563		T.aestivum	Central
193564		T.aestivum	Central
193565		T.aestivum	Central
193566		T.aestivum	Central

193567		T.aestivum	Central
193568		T.aestivum	Central
193569		T.aestivum	Central
193570		T.aestivum	Central
193571		T.aestivum	Central
193572		T.aestivum	Central
193573	√	T.aestivum	Central
193574		T.aestivum	Central
193575		T.aestivum	Central
193576		T.aestivum	Central
193577		T.aestivum	Central
193578		T.aestivum	Central
193579		T.aestivum	Central
193580		T.aestivum	Central
193581		T.aestivum	Central
193582		T.aestivum	Central
193583		T.aestivum	Central
193584	√	T.aestivum	Central
193585		T.aestivum	Central
193586		T.aestivum	Central
193587		T.aestivum	Central
193588		T.aestivum	Central
193589	√	T.aestivum	Central
193590	√	T.aestivum	Central
193591		T.aestivum	Central
193592		T.aestivum	Central
193593		T.aestivum	Central
193594		T.aestivum	Central
193595	√	T.aestivum	Central
193597		T.aestivum	Central
193598		T.aestivum	Central
193599		T.aestivum	Central
193600		T.aestivum	Central
193601	√	T.aestivum	Central
193603		T.aestivum	Central
193604		T.aestivum	Central
193606		T.aestivum	Central
193607		T.aestivum	Central
193609		T.aestivum	Central
193610		T.aestivum	Central
193611		T.aestivum	Central

193612		T.aestivum	Central
193613		T.aestivum	Central
193614		T.aestivum	Central
193615		T.aestivum	Central
193616		T.aestivum	Central
193617	√	T.aestivum	Central
193618		T.aestivum	Central
193619	√	T.aestivum	Central
193620		T.aestivum	Central
193621		T.aestivum	Central
193622		T.aestivum	Central
193623		T.aestivum	Central
193624		T.aestivum	Central
193625		T.aestivum	Central
193626		T.aestivum	Central
193627		T.aestivum	Central
193628		T.aestivum	Central
193629	√	T.aestivum	Central
193630		T.aestivum	Central
193631		T.aestivum	Central
193632		T.aestivum	Central
193633		T.aestivum	Central
193634		T.aestivum	Central
193635		T.aestivum	Central
193636		T.aestivum	Central
193637		T.aestivum	Central
193638		t.aestivum	Central
193639		T.aestivum	Central
193640		T.aestivum	Central
193641		T.aestivum	Central
193642		T.aestivum	Central
193643		T.aestivum	Central
193644		T.aestivum	Central
193645	√	T.aestivum	Central
193646		T.aestivum	Central
193647		T.aestivum	Central
193648	√	T.aestivum	Central
193649		T.aestivum	Central
193650		T.aestivum	Central
193651		T.aestivum	Central
193652		T.aestivum	Central

193653	√	T.aestivum	Central
193654		T.aestivum	Central
193655		T.aestivum	Central
193656		T.aestivum	Central
193657	√	T.aestivum	Central
193658		T.aestivum	Central
193659		T.aestivum	Central
193660		T.aestivum	Central
193661		T.aestivum	Central
193662		T.aestivum	Central
193663		T.aestivum	Central
193664		T.aestivum	Central
193665		T.aestivum	Central
193666		T.aestivum	Central
193667		T.aestivum	Central
193668	√	T.aestivum	Central
193669		T.aestivum	Central
193670		T.aestivum	Central
193671	√	T.aestivum	Central
193672		T.aestivum	Central
193673	√	T.aestivum	Central
193674		T.aestivum	Central
193675		T.aestivum	Central
193676		T.aestivum	Central
193677	√	T.aestivum	Central
193679		T.aestivum	Central
193682	√	T.aestivum	Central
193683		T.aestivum	Central
193684		T.aestivum	Central
193685		T.aestivum	Central
193686		T.aestivum	Central
193687		T.aestivum	Central
193688		T.aestivum	Central
193689		T.aestivum	Central
193690		T.aestivum	Central
193691		T.aestivum	Central
193692		T.aestivum	Central
193693		T.aestivum	Central
193694		T.aestivum	Central
193695		T.aestivum	Central
193696		T.aestivum	Central

193697		T.aestivum	Central
193698		T.aestivum	Central
193699		T.aestivum	Central
193700	√	T.aestivum	Central
193701		T.aestivum	Central
193702		T.aestivum	Central
193703		T.aestivum	Central
193704		T.aestivum	Central
193705		T.aestivum	Central
193706		T.aestivum	Central
193707		T.aestivum	Central
193709		T.aestivum	Central
193710		T.aestivum	Central
193711		T.aestivum	Central
193712		T.aestivum	Central
193713		T.aestivum	Central
193714		T.aestivum	Central
193715	√	T.aestivum	Central
193716		T.aestivum	Central
193717	√	T.aestivum	Central
193718		T.aestivum	Central
193719		T.aestivum	Central
193720		T.aestivum	Central
193722		T.aestivum	Central
193725		T.aestivum	Central
193728	√	T.aestivum	Central
193730		T.aestivum	Central
193732		T.aestivum	Central
193733		T.aestivum	Central
193734		T.aestivum	Central
193735		T.aestivum	Central
193736		T.aestivum	Central
193737	√	T.aestivum	Central
193738	√	T.aestivum	Central
193739		T.aestivum	Central
193740		T.aestivum	Central
193741		T.aestivum	Central
193742		T.aestivum	Central
193743		T.aestivum	Central
193744		T.aestivum	Central
193745	√	T.aestivum	Central

193746		T.aestivum	Central
193747		T.aestivum	Central
193748	√	T.aestivum	Central
193749		T.aestivum	Central
193750		T.aestivum	Central
193751		T.aestivum	Central
193752	√	T.aestivum	Central
193753	√	T.aestivum	Central
193754		T.aestivum	Central
193755		T.aestivum	Central
193756		T.aestivum	Central
193757		T.aestivum	Central
193758		T.aestivum	Central
193759	√	T.aestivum	Central
193760	√	T.aestivum	Central
193761		T.aestivum	Central
193762		T.aestivum	Central
193763	√	T.aestivum	Central
193764		T.aestivum	Central
193767		T.aestivum	Central
193768		T.aestivum	Central
193769		T.aestivum	Central
193770	√	T.aestivum	Central
193771		T.aestivum	Central
193772		T.aestivum	Central
193773		T.aestivum	Central
193774		T.aestivum	Central
193776		T.aestivum	Central
193778		T.aestivum	Central
193779		T.aestivum	Central
193780		T.aestivum	Central
193781		T.aestivum	Central
193782		T.aestivum	Central
193784		T.aestivum	Central
193785	√	T.aestivum	Central
193786		T.aestivum	Central
193787		T.aestivum	Central
193788		T.aestivum	Central
193789	√	T.aestivum	Central
193790		T.aestivum	Central
193791		T.aestivum	Central

193792	√	T.aestivum	Central
193793		T.aestivum	Central
193794		T.aestivum	Central
193795	√	T.aestivum	Central
193798		T.aestivum	Central
193799	√	T.aestivum	Central
193800		T.aestivum	Central
193802		T.aestivum	Central
193803		T.aestivum	Central
193804		T.aestivum	Central
193806		T.aestivum	Central
193807	√	T.aestivum	Central
193808		T.aestivum	Central
193809		T.aestivum	Central
193810		T.aestivum	Central
193811		T.aestivum	Central
193812		T.aestivum	Central
193813		T.aestivum	Central
193814		T.aestivum	Central
193815	√	T.aestivum	Central
193816		T.aestivum	Central
193817		T.aestivum	Central
193818	√	T.aestivum	Central
193819		T.aestivum	Central
193821		T.aestivum	Central
193822		T.aestivum	Central
193823		T.aestivum	Central
193824	√	T.aestivum	Central
193825		T.aestivum	Central
193826		T.aestivum	Central
193827	√	T.aestivum	Central
193828	√	T.aestivum	Central
193829		T.aestivum	Central
193830		T.aestivum	Central
193831		T.aestivum	Central
193833		T.aestivum	Central
193834		T.aestivum	Central
193835		T.aestivum	Central
193836		T.aestivum	Central
193837	√	T.aestivum	Central
193838		T.aestivum	Central

193839		T.aestivum	Central
193840		T.aestivum	Central
193841		T.aestivum	Central
193843	√	T.aestivum	Central
193844		T.aestivum	Central
193845		T.aestivum	Central
193846		T.aestivum	Central
193847		T.aestivum	Central
193848		T.aestivum	Central
193849		T.aestivum	Central
193850		T.aestivum	Central
193851		T.aestivum	Central
193852		T.aestivum	Central
193853		T.aestivum	Central
193854	√	T.aestivum	Central
193855		T.aestivum	Central
193856		T.aestivum	Central
193857		T.aestivum	Central
193858		T.aestivum	Central
193859		T.aestivum	Central
193861		T.aestivum	Central
193862		T.aestivum	Central
193863		T.aestivum	Central
193864	√	T.aestivum	Central
193865		T.aestivum	Central
193866		T.aestivum	Central
193867		T.aestivum	Central
193868	√	T.aestivum	Central
193869		T.aestivum	Central
193870		T.aestivum	Central
193871		T.aestivum	Central
193872		T.aestivum	Central
193873		T.aestivum	Central
193874		T.aestivum	Central
193875		T.aestivum	Central
193876		T.aestivum	Central
193877	√	T.aestivum	Central
193878		T.aestivum	Central
193879		T.aestivum	Central
193880		T.aestivum	Central
193881		T.aestivum	Central

193882		T.aestivum	Central
193884		T.aestivum	Central
193885	√	T.aestivum	Central
193886		T.aestivum	Central
193887		T.aestivum	Central
193888		T.aestivum	Central
193889		T.aestivum	Central
193890		T.aestivum	Central
193891		T.aestivum	Central
193892		T.aestivum	Central
193893		T.aestivum	Central
193894		T.aestivum	Central
193895		T.aestivum	Central
193896	√	T.aestivum	Central
193897		T.aestivum	Central
193898	√	T.aestivum	Central
193899		T.aestivum	Central
193900	√	T.aestivum	Central
193901	√	T.aestivum	Central
193902		T.aestivum	Central
193903		T.aestivum	Central
193904	√	T.aestivum	Central
193905		T.aestivum	Central
193906		T.aestivum	Central
193907	√	T.aestivum	Central
193908		T.aestivum	Central
193909		T.aestivum	Central
193910		T.aestivum	Central
193911	√	T.aestivum	Central
193912		T.aestivum	Central
193913		T.aestivum	Central
193914		T.aestivum	Central
193915		T.aestivum	Central
193916		T.aestivum	Central
193917		T.aestivum	Central
193918		T.aestivum	Central
193919		T.aestivum	Central
193920	√	T.aestivum	Central
193921		T.aestivum	Central
193922		T.aestivum	Central
193923		T.aestivum	Central

193924		T.aestivum	Central
193925		T.aestivum	Central
193926		T.aestivum	Central
193927		T.aestivum	Central
193928		T.aestivum	Central
193929		T.aestivum	Central
193930		T.aestivum	Central
193931		T.aestivum	Central
193932		T.aestivum	Central
193933		T.aestivum	Central
193934		T.aestivum	Central
193935		T.aestivum	Central
193936		T.aestivum	Central
193937		T.aestivum	Central
193938		T.aestivum	Central
193939		T.aestivum	Central
193940		T.aestivum	Central
193941		T.aestivum	Central
193942		T.aestivum	Central
193943		T.aestivum	Central
193944		T.aestivum	Central
193945		T.aestivum	Central
193946		T.aestivum	Central
193947		T.aestivum	Central
193948		T.aestivum	Central
193949		T.aestivum	Central
193950	√	T.aestivum	Central
193951		T.aestivum	Central
193952		T.aestivum	Central
193953		T.aestivum	Central
193954		T.aestivum	Central
193955		T.aestivum	Central
193956		T.aestivum	Central
193957		T.aestivum	Central
193958		T.aestivum	Central
193959	√	T.aestivum	Central
193960		T.aestivum	Central
193961	√	T.aestivum	Central
193962		T.aestivum	Central
193963		T.aestivum	Central
193964		T.aestivum	Central

193965	√	T.aestivum	Central
193966		T.aestivum	Central
193967		T.aestivum	Central
193968		T.aestivum	Central
193969		T.aestivum	Central
193970		T.aestivum	Central
193971		T.aestivum	Central
193972		T.aestivum	Central
193973		T.aestivum	Central
193974	√	T.aestivum	Central
193975		T.aestivum	Central
193976		T.aestivum	Central
193977		T.aestivum	Central
193978		T.aestivum	Central
193980		T.aestivum	Central
193981		T.aestivum	Central
193982		T.aestivum	Central
193983		T.aestivum	Central
193984		T.aestivum	Central
193985		T.aestivum	Central
193986		T.aestivum	Central
193987		T.aestivum	Central
193988		T.aestivum	Central
193989	√	T.aestivum	Central
193990		T.aestivum	Central
193991		T.aestivum	Central
193992		T.aestivum	Central
193993		T.aestivum	Central
193994		T.aestivum	Central
193995		T.aestivum	Central
193996		T.aestivum	Central
193997	√	T.aestivum	Central
193998		T.aestivum	Central
193999		T.aestivum	Central
194000	√	T.aestivum	Central
194001	√	T.aestivum	Central
194002		T.aestivum	Central
194003		T.aestivum	Central
194004		T.aestivum	Central
194005		T.aestivum	Central
194006		T.aestivum	Central

194007		T.aestivum	Central
194008		T.aestivum	Central
194009		T.aestivum	Central
194010		T.aestivum	Central
194011		T.aestivum	Central
194012		T.aestivum	Central
194013		T.aestivum	Central
194014		T.aestivum	Central
194015		T.aestivum	Central
194016		T.aestivum	Central
194017		T.aestivum	Central
194018		T.aestivum	Central
194019		T.aestivum	Central
194020		T.aestivum	Central
194021		T.aestivum	Central
194022		T.aestivum	Central
194023		T.aestivum	Central
194024		T.aestivum	Central
194025		T.aestivum	Central
194026	√	T.aestivum	Central
194027	√	T.aestivum	Central
194028		T.aestivum	Central
194029		T.aestivum	Central
194030		T.aestivum	Central
194031		T.aestivum	Central
194032		T.aestivum	Central
194033		T.aestivum	Central
194034		T.aestivum	Central
194035		T.aestivum	Central
194036		T.aestivum	Central
194037		T.aestivum	Central
194038		T.aestivum	Central
194039		T.aestivum	Central
194040		T.aestivum	Central
194041		T.aestivum	Central
194042		T.aestivum	Central
194043		T.aestivum	Central
194044		T.aestivum	Central
194045		T.aestivum	Central
194046	√	T.aestivum	Central
194047		T.aestivum	Central

194048		T.aestivum	Central
194049		T.aestivum	Central
194050		T.aestivum	Central
194051		T.aestivum	Central
194052	√	T.aestivum	Central
194053		T.aestivum	Central
194054		T.aestivum	Central
194055		T.aestivum	Central
194056		T.aestivum	Central
194057		T.aestivum	Central
194058		T.aestivum	Central
194059	√	T.aestivum	Central
194060	√	T.aestivum	Central
194061		T.aestivum	Central
194062		T.aestivum	Central
194063		T.aestivum	Central
194064		T.aestivum	Central
194065		T.aestivum	Central
194066	√	T.aestivum	Central
194067		T.aestivum	Central
194068		T.aestivum	Central
194069		T.aestivum	Central
194070		T.aestivum	Central
194071		T.aestivum	Central
194072		T.aestivum	Central
194073	√	T.aestivum	Central
194074		T.aestivum	Central
194075		T.aestivum	Central
194076		T.aestivum	Central
194077		T.aestivum	Central
194078		T.aestivum	Central
194079	√	T.aestivum	Central
194080		T.aestivum	Central
194081		T.aestivum	Central
194083		T.aestivum	Central
194084	√	T.aestivum	Central
194085		T.aestivum	Central
194086		T.aestivum	Central
194087		T.aestivum	Central
194088	√	T.aestivum	Central
194089	√	T.aestivum	Central

194090		T.aestivum	Central
194091		T.aestivum	Central
194092		T.aestivum	Central
194093		T.aestivum	Central
194094		T.aestivum	Central
194096		T.aestivum	Central
194097		T.aestivum	Central
194098		T.aestivum	Central
194099		T.aestivum	Central
194100		T.aestivum	Central
194101	√	T.aestivum	Central
194102		T.aestivum	Central
194103		T.aestivum	Central
194104		T.aestivum	Central
194105		T.aestivum	Central
194106		T.aestivum	Central
194107		T.aestivum	Central
194108		T.aestivum	Central
194109		T.aestivum	Central
194110		T.aestivum	Central
194111	√	T.aestivum	Central
194112		T.aestivum	Central
194113	√	T.aestivum	Central
194114		T.aestivum	Central
194115		T.aestivum	Central
194116		T.aestivum	Central
194117	√	T.aestivum	Central
194118		T.aestivum	Central
194119		T.aestivum	Central
194120		T.aestivum	Central
194121		T.aestivum	Central
194122		T.aestivum	Central
194123		T.aestivum	Central
194124		T.aestivum	Central
194125		T.aestivum	Central
194126		T.aestivum	Central
194127		T.aestivum	Central
194128		T.aestivum	Central
194129		T.aestivum	Central
194130		T.aestivum	Central
194131		T.aestivum	Central

194134		T.aestivum	Central
194135		T.aestivum	Central
194136		T.aestivum	Central
194137		T.aestivum	Central
194138	√	T.aestivum	Central
194139	√	T.aestivum	Central
194140		T.aestivum	Central
194141		T.aestivum	Central
194142		T.aestivum	Central
194143		T.aestivum	Central
194144		T.aestivum	Central
194145		T.aestivum	Central
194146	√	T.aestivum	Central
194147		T.aestivum	Central
194148		T.aestivum	Central
194149		T.aestivum	Central
194150		T.aestivum	Central
194151		T.aestivum	Central
194152		T.aestivum	Central
194153		T.aestivum	Central
194154		T.aestivum	Central
194155		T.aestivum	Central
194156		T.aestivum	Central
194157	√	T.aestivum	Central
194158	√	T.aestivum	Central
194159		T.aestivum	Central
194161		T.aestivum	Central
194162		T.aestivum	Central
194163		T.aestivum	Central
194164		T.aestivum	Central
194165		T.aestivum	Central
194166		T.aestivum	Central
194167		T.aestivum	Central
194168		T.aestivum	Central
194169		T.aestivum	Central
194171		T.aestivum	Central
194172		T.aestivum	Central
194173		T.aestivum	Central
194174		T.aestivum	Central
194175		T.aestivum	Central
194176		T.aestivum	Central

194177		T.aestivum	Central
194178	√	T.aestivum	Central
194179		T.aestivum	Central
194180		T.aestivum	Central
194181	√	T.aestivum	Central
194182		T.aestivum	Central
194183	√	T.aestivum	Central
194184		T.aestivum	Central
194185	√	T.aestivum	Central
194186		T.aestivum	Central
194187	√	T.aestivum	Central
194188		T.aestivum	Central
194189	√	T.aestivum	Central
194190		T.aestivum	Central
194191		T.aestivum	Central
194192		T.aestivum	Central
194193		T.aestivum	Central
194194		T.aestivum	Central
194195		T.aestivum	Central
194196		T.aestivum	Central
194197		T.aestivum	Central
194198		T.aestivum	Central
194199		T.aestivum	Central
194200		T.aestivum	Central
194201		T.aestivum	Central
194202		T.aestivum	Central
194203		T.aestivum	Central
194204		T.aestivum	Central
194205	√	T.aestivum	Central
194206		T.aestivum	Central
194207		T.aestivum	Central
194208		T.aestivum	Central
194209		T.aestivum	Central
194210		T.aestivum	Central
194211		T.aestivum	Central
194212		T.aestivum	Central
194213		T.aestivum	Central
194214		T.aestivum	Central
194215		T.aestivum	Central
194216		T.aestivum	Central
194217		T.aestivum	Central

194218		T.aestivum	Central
194219		T.aestivum	Central
194220		T.aestivum	Central
194221	√	T.aestivum	Central
194222		T.aestivum	Central
194223		T.aestivum	Central
194224		T.aestivum	Central
194225		T.aestivum	Central
194226		T.aestivum	Central
194227		T.aestivum	Central
194228		T.aestivum	Central
194229		T.aestivum	Central
194230	√	T.aestivum	Central
194231		T.aestivum	Central
194232		T.aestivum	Central
194233		T.aestivum	Central
194234		T.aestivum	Central
194235		T.aestivum	Central
194236		T.aestivum	Central
194237		T.aestivum	Central
194238		T.aestivum	Central
194239	√	T.aestivum	Central
194240		T.aestivum	Central
194241		T.aestivum	Central
194242		T.aestivum	Central
194243		T.aestivum	Central
194244		T.aestivum	Central
194245	√	T.aestivum	Central
194246		T.aestivum	Central
194247		T.aestivum	Central
194248		T.aestivum	Central
194249		T.aestivum	Central
194250	√	T.aestivum	Central
194251		T.aestivum	Central
194252		T.aestivum	Central
194253		T.aestivum	Central
194254		T.aestivum	Central
194255		T.aestivum	Central
194256		T.aestivum	Central
194257	√	T.aestivum	Central
194258		T.aestivum	Central

194259		T.aestivum	Central
194260		T.aestivum	Central
194261		T.aestivum	Central
194262		T.aestivum	Central
194263		T.aestivum	Central
194264		T.aestivum	Central
194265		T.aestivum	Central
194266		T.aestivum	Central
194267		T.aestivum	Central
194268		T.aestivum	Central
194269		T.aestivum	Central
194270		T.aestivum	Central
194271	√	T.aestivum	Central
194272		T.aestivum	Central
194273		T.aestivum	Central
194274		T.aestivum	Central
194275		T.aestivum	Central
194276	√	T.aestivum	Central
194277		T.aestivum	Central
194278		T.aestivum	Central
194279		T.aestivum	Central
194280		T.aestivum	Central
194281		T.aestivum	Central
194283		T.aestivum	Central
194285	√	T.aestivum	Central
194288	√	T.aestivum	Central
194291	√	T.aestivum	Central
194292		T.aestivum	Central
194293		T.aestivum	Central
194294		T.aestivum	Central
194295		T.aestivum	Central
194296		T.aestivum	Central
194297		T.aestivum	Central
194298	√	T.aestivum	Central
194299		T.aestivum	Central
194300	√	T.aestivum	Central
194301		T.aestivum	Central
194302	√	T.aestivum	Central
194303	√	T.aestivum	Central
194304		T.aestivum	Central
194305		T.aestivum	Central

194306		T.aestivum	Central
194308		T.aestivum	Central
194309		T.aestivum	Central
194310		T.aestivum	Central
194311		T.aestivum	Central
194313		T.aestivum	Central
194314		T.aestivum	Central
194315		T.aestivum	Central
194316		T.aestivum	Central
194317		T.aestivum	Central
194318		T.aestivum	Central
194319		T.aestivum	Central
194320		T.aestivum	Central
194321		T.aestivum	Central
194322		T.aestivum	Central
194323		T.aestivum	Central
194324		T.aestivum	Central
194325		T.aestivum	Central
194326		T.aestivum	Central
194327		T.aestivum	Central
194328		T.aestivum	Central
194329		T.aestivum	Central
194330		T.aestivum	Central
194331	√	T.aestivum	Central
194332		T.aestivum	Central
194333		T.aestivum	Central
194334		T.aestivum	Central
194335		T.aestivum	Central
194336		T.aestivum	Central
194337		T.aestivum	Central
194338		T.aestivum	Central
194339		T.aestivum	Central
194340		T.aestivum	Central
194341		T.aestivum	Central
194342		T.aestivum	Central
194343		T.aestivum	Central
194344		T.aestivum	Central
194345		T.aestivum	Central
194346		T.aestivum	Central
194347		T.aestivum	Central
194348		T.aestivum	Central

194349		T.aestivum	Central
194350		T.aestivum	Central
194351		T.aestivum	Central
194352		T.aestivum	Central
194353		T.aestivum	Central
194354		T.aestivum	Central
194355		T.aestivum	Central
194356		T.aestivum	Central
194357		T.aestivum	Central
194358	√	T.aestivum	Central
194359		T.aestivum	Central
194360		T.aestivum	Central
194361		T.aestivum	Central
194362		T.aestivum	Central
194363	√	T.aestivum	Central
194364		T.aestivum	Central
194365		T.aestivum	Central
194366		T.aestivum	Central
194367		T.aestivum	Central
194368		T.aestivum	Central
194369		T.aestivum	Central
194370	√	T.aestivum	Central
194371		T.aestivum	Central
194372		T.aestivum	Central
194373		T.aestivum	Central
194374		T.aestivum	Central
194375	√	T.aestivum	Central
194376		T.aestivum	Central
194377		T.aestivum	Central
194378		T.aestivum	Central
194379		T.aestivum	Central
194380		T.aestivum	Central
194381		T.aestivum	Central
194382		T.aestivum	Central
194383		T.aestivum	Central
194384		T.aestivum	Central
194385		T.aestivum	Central
194386		T.aestivum	Central
194387		T.aestivum	Central
194388		T.aestivum	Central
194389		T.aestivum	Central

194390		T.aestivum	Central
194391		T.aestivum	Central
194392		T.aestivum	Central
194393		T.aestivum	Central
194394		T.aestivum	Central
194395		T.aestivum	Central
194396		T.aestivum	Central
194397		T.aestivum	Central
194398		T.aestivum	Central
194399		T.aestivum	Central
194400		T.aestivum	Central
194402		T.aestivum	Central
194404	√	T.aestivum	Central
194405		T.aestivum	Central
194406		T.aestivum	Central
194407	√	T.aestivum	Central
194408		T.aestivum	Central
194409		T.aestivum	Central
194410		T.aestivum	Central
194411		T.aestivum	Central
194413		T.aestivum	Central
194414		T.aestivum	Central
194415		T.aestivum	Central
194416		T.aestivum	Central
194417		T.aestivum	Central
194418		T.aestivum	Central
194419		T.aestivum	Central
194420		T.aestivum	Central
194421		T.aestivum	Central
194422	√	T.aestivum	Central
194423		T.aestivum	Central
194424		T.aestivum	Central
194425		T.aestivum	Central
194426		T.aestivum	Central
194427		T.aestivum	Central
194428		T.aestivum	Central
194429		T.aestivum	Central
194430		T.aestivum	Central
194431		T.aestivum	Central
194432		T.aestivum	Central
194433		T.aestivum	Central

194434		T.aestivum	Central
194435		T.aestivum	Central
194436		T.aestivum	Central
194437	√	T.aestivum	Central
194438		T.aestivum	Central
194439	√	T.aestivum	Central
194441		T.aestivum	Central
194442		T.aestivum	Central
194443	√	T.aestivum	Central
194444		T.aestivum	Central
194445		T.aestivum	Central
194446		T.aestivum	Central
194447		T.aestivum	Central
194448		T.aestivum	Central
194449		T.aestivum	Central
194450		T.aestivum	Central
194451		T.aestivum	Central
194452		T.aestivum	Central
194453		T.aestivum	Central
194454		T.aestivum	Central
194455		T.aestivum	Central
194456		T.aestivum	Central
194457		T.aestivum	Central
194458		T.aestivum	Central
194459		T.aestivum	Central
194460		T.aestivum	Central
194461		T.aestivum	Central
194463		T.aestivum	Central
194464		T.aestivum	Central
194465	√	T.aestivum	Central
194466		T.aestivum	Central
194467		T.aestivum	Central
194468		T.aestivum	Central
194469		T.aestivum	Central
194470	√	T.aestivum	Central
194471		T.aestivum	Central
194472		T.aestivum	Central
194473		T.aestivum	Central
194474		T.aestivum	Central
194475		T.aestivum	Central
194476		T.aestivum	Central

194477		T.aestivum	Central
194478		T.aestivum	Central
194479		T.aestivum	Central
194480		T.aestivum	Central
194481		T.aestivum	Central
194482		T.aestivum	Central
194483	√	T.aestivum	Central
194485		T.aestivum	Central
194486		T.aestivum	Central
194487		T.aestivum	Central
194488		T.aestivum	Central
194489		T.aestivum	Central
194490		T.aestivum	Central
194491		T.aestivum	Central
194492		T.aestivum	Central
194493		T.aestivum	Central
194494	√	T.aestivum	Central
194495		T.aestivum	Central
194496		T.aestivum	Central
194497		T.aestivum	Central
194498		T.aestivum	Central
194499		T.aestivum	Central
194500		T.aestivum	Central
194501		T.aestivum	Central
194502		T.aestivum	Central
194503		T.aestivum	Central
194504		T.aestivum	Central
194505		T.aestivum	Central
194506		T.aestivum	Central
194507		T.aestivum	Central
194508		T.aestivum	Central
194509	√	T.aestivum	Central
194510		T.aestivum	Central
194511	√	T.aestivum	Central
194512		T.aestivum	Central
194513		T.aestivum	Central
194514		T.aestivum	Central
194515		T.aestivum	Central
194516		T.aestivum	Central
194517		T.aestivum	Central
194519		T.aestivum	Central

194520	√	T.aestivum	Central
194521		T.aestivum	Central
194522		T.aestivum	Central
194523		T.aestivum	Central
194524		T.aestivum	Central
194525	√	T.aestivum	Central
194526		T.aestivum	Central
194527		T.aestivum	Central
194528		T.aestivum	Central
194529	√	T.aestivum	Central
194530		T.aestivum	Central
194531	√	T.aestivum	Central
194532		T.aestivum	Central
194533		T.aestivum	Central
194534		T.aestivum	Central
194535	√	T.aestivum	Central
194536		T.aestivum	Central
194537		T.aestivum	Central
194538		T.aestivum	Central
194539		T.aestivum	Central
194540		T.aestivum	Central
194541		T.aestivum	Central
194542		T.aestivum	Central
194543		T.aestivum	Central
194544		T.aestivum	Central
194545		T.aestivum	Central
194546		T.aestivum	Central
194547	√	T.aestivum	Central
194548		T.aestivum	Central
194549		T.aestivum	Central
194550		T.aestivum	Central
194551		T.aestivum	Central
194552		T.aestivum	Central
194553		T.aestivum	Central
194554		T.aestivum	Central
194555		T.aestivum	Central
194556		T.aestivum	Central
194557		T.aestivum	Central
194558		T.aestivum	Central
194559		T.aestivum	Central
194560		T.aestivum	Central

194561		T.aestivum	Central
194562		T.aestivum	Central
194563		T.aestivum	Central
194564		T.aestivum	Central
194565		T.aestivum	Central
194566		T.aestivum	Central
194567		T.aestivum	Central
194568		T.aestivum	Central
194569		T.aestivum	Central
194570		T.aestivum	Central
194571		T.aestivum	Central
194572		T.aestivum	Central
194573		T.aestivum	Central
194574		T.aestivum	Central
194575		T.aestivum	Central
194576		T.aestivum	Central
194577		T.aestivum	Central
194578		T.aestivum	Central
194579		T.aestivum	Central
194580		T.aestivum	Central
194581		T.aestivum	Central
194582		T.aestivum	Central
194583		T.aestivum	Central
194584		T.aestivum	Central
194585		T.aestivum	Central
194586		T.aestivum	Central
194587		T.aestivum	Central
194588		T.aestivum	Central
194589		T.aestivum	Central
194590		T.aestivum	Central
194591		T.aestivum	Central
194592		T.aestivum	Central
194593	√	T.aestivum	Central
194594		T.aestivum	Central
194595		T.aestivum	Central
194596		T.aestivum	Central
194597		T.aestivum	Central
194598		T.aestivum	Central
194599		T.aestivum	Central
194600	√	T.aestivum	Central
194601		T.aestivum	Central

194602	√	T.aestivum	Central
194603		T.aestivum	Central
194604		T.aestivum	Central
194605		T.aestivum	Central
194606		T.aestivum	Central
194608	√	T.aestivum	Central
194609		T.aestivum	Central
194611		T.aestivum	Central
194612		T.aestivum	Central
194613		T.aestivum	Central
194614		T.aestivum	Central
194615	√	T.aestivum	Central
194616	√	T.aestivum	Central
194617		T.aestivum	Central
194618	√	T.aestivum	Central
194619	√	T.aestivum	Central
194620		T.aestivum	Central
194621		T.aestivum	Central
194622	√	T.aestivum	Central
194623		T.aestivum	Central
194624		T.aestivum	Central
194625		T.aestivum	Central
194626		T.aestivum	Central
194627	√	T.aestivum	Central
194628	√	T.aestivum	Central
194629		T.aestivum	Central
194630		T.aestivum	Central
194632		T.aestivum	Central
194633	√	T.aestivum	Central
194634		T.aestivum	Central
194635		T.aestivum	Central
194636		T.aestivum	Central
194637		T.aestivum	Central
194638		T.aestivum	Central
194639		T.aestivum	Central
194640		T.aestivum	Central
194641		T.aestivum	Central
194642	√	T.aestivum	Central
194643		T.aestivum	Central
194644	√	T.aestivum	Central
194645		T.aestivum	Central

194646		T.aestivum	Central
194647		T.aestivum	Central
194648	√	T.aestivum	Central
194649		T.aestivum	Central
194650		T.aestivum	Central
194651		T.aestivum	Central
194652		T.aestivum	Central
194653		T.aestivum	Central
194654		T.aestivum	Central
194655		T.aestivum	Central
194656		T.aestivum	Central
194657		T.aestivum	Central
194658		T.aestivum	Central
194659		T.aestivum	Central
194660		T.aestivum	Central
194661		T.aestivum	Central
194662		T.aestivum	Central
194663		T.aestivum	Central
194664		T.aestivum	Central
194665		T.aestivum	Central
194666		T.aestivum	Central
194667	√	T.aestivum	Central
194668		T.aestivum	Central
194669		T.aestivum	Central
194670	√	T.aestivum	Central
194671		T.aestivum	Central
194672		T.aestivum	Central
194673	√	T.aestivum	Central
194674		T.aestivum	Central
194675		T.aestivum	Central
194677		T.aestivum	Central
194678		T.aestivum	Central
194679		T.aestivum	Central
194680	√	T.aestivum	Central
194681		T.aestivum	Central
194682		T.aestivum	Central
194683		T.aestivum	Central
194685		T.aestivum	Central
194686	√	T.aestivum	Central
194687	√	T.aestivum	Central
194689		T.aestivum	Central

194690		T.aestivum	Central
194691	√	T.aestivum	Central
194692		T.aestivum	Central
194693		T.aestivum	Central
194695		T.aestivum	Central
194696		T.aestivum	Central
194697	√	T.aestivum	Central
194698		T.aestivum	Central
194699		T.aestivum	Central
194700		T.aestivum	Central
194701	√	T.aestivum	Central
194702		T.aestivum	Central
194703		T.aestivum	Central
194704		T.aestivum	Central
194705		T.aestivum	Central
194706		T.aestivum	Central
194707		T.aestivum	Central
194708		T.aestivum	Central
194709		T.aestivum	Central
194710	√	T.aestivum	Central
194711		T.aestivum	Central
194712		T.aestivum	Central
194713		T.aestivum	Central
194714	√	T.aestivum	Central
194715		T.aestivum	Central
194716		T.aestivum	Central
194717		T.aestivum	Central
194718		T.aestivum	Central
194719		T.aestivum	Central
194720		T.aestivum	Central
194721		T.aestivum	Central
194722	√	T.aestivum	Central
194723		T.aestivum	Central
194724		T.aestivum	Central
194725		T.aestivum	Central
194726		T.aestivum	Central
194727		T.aestivum	Central
194728		T.aestivum	Central
194729	√	T.aestivum	Central
194730		T.aestivum	Central
194731		T.aestivum	Central

194732	√	T.aestivum	Central
194733		T.aestivum	Central
194734		T.aestivum	Central
194735		T.aestivum	Central
194736		T.aestivum	Central
194737		T.aestivum	Central
194738		T.aestivum	Central
194739		T.aestivum	Central
194740		T.aestivum	Central
194741		T.aestivum	Central
194742	√	T.aestivum	Central
194743	√	T.aestivum	Central
194744		T.aestivum	Central
194745		T.aestivum	Central
194746	√	T.aestivum	Central
194747	√	T.aestivum	Central
194748		T.aestivum	Central
194749		T.aestivum	Central
194750		T.aestivum	Central
194751		T.aestivum	Central
194752		T.aestivum	Central
194753		T.aestivum	Central
194754		T.aestivum	Central
194755		T.aestivum	Central
194759		T.aestivum	Central
194760		T.aestivum	Central
194761		T.aestivum	Central
194762		T.aestivum	Central
194763		T.aestivum	Central
194765		T.aestivum	Central
194766		T.aestivum	Central
194767		T.aestivum	Central
194768		T.aestivum	Central
194769		T.aestivum	Central
194770		T.aestivum	Central
194772		T.aestivum	Central
194773		T.aestivum	Central
194774	√	T.aestivum	Central
194775		T.aestivum	Central
194776		T.aestivum	Central
194777		T.aestivum	Central

194778		T.aestivum	Central
194780		T.aestivum	Central
194781		T.aestivum	Central
194782		T.aestivum	Central
194783		T.aestivum	Central
194784	√	T.aestivum	Central
194785	√	T.aestivum	Central
194786	√	T.aestivum	Central
194787		T.aestivum	Central
194788		T.aestivum	Central
194789		T.aestivum	Central
194790		T.aestivum	Central
194791		T.aestivum	Central
194792		T.aestivum	Central
194793		T.aestivum	Central
194794		T.aestivum	Central
194795		T.aestivum	Central
194796	√	T.aestivum	Central
194797		T.aestivum	Central
194798		T.aestivum	Central
194799		T.aestivum	Central
194800		T.aestivum	Central
194801		T.aestivum	Central
194802		T.aestivum	Central
194803	√	T.aestivum	Central
194804		T.aestivum	Central
194805		T.aestivum	Central
194806		T.aestivum	Central
194807	√	T.aestivum	Central
194808		T.aestivum	Central
194809		T.aestivum	Central
194810		T.aestivum	Central
194811		T.aestivum	Central
194813		T.aestivum	Central
194814	√	T.aestivum	Central
194815	√	T.aestivum	Central
194816		T.aestivum	Central
194817		T.aestivum	Central
194818		T.aestivum	Central
194821		T.aestivum	Central
194822		T.aestivum	Central

194823		T.aestivum	Central
194824		T.aestivum	Central
194825		T.aestivum	Central
194826		T.aestivum	Central
194827		T.aestivum	Central
194828		T.aestivum	Central
194829		T.aestivum	Central
194830		T.aestivum	Central
194831	√	T.aestivum	Central
194832		T.aestivum	Central
194833		T.aestivum	Central
194834		T.aestivum	Central
194835		T.aestivum	Central
194836		T.aestivum	Central
194837		T.aestivum	Central
194838		T.aestivum	Central
194839		T.aestivum	Central
194840		T.aestivum	Central
194841		T.aestivum	Central
194842		T.aestivum	Central
194843		T.aestivum	Central
194844		T.aestivum	Central
194845		T.aestivum	Central
194846	√	T.aestivum	Central
194847	√	T.aestivum	Central
194848		T.aestivum	Central
194849		T.aestivum	Central
194850		T.aestivum	Central
194854		T.aestivum	Central
194855		T.aestivum	Central
194856		T.aestivum	Central
194857		T.aestivum	Central
194858		T.aestivum	Central
194859		T.aestivum	Central
194860	√	T.aestivum	Central
194861		T.aestivum	Central
194862		T.aestivum	Central
194863		T.aestivum	Central
194864		T.aestivum	Central
194867		T.aestivum	Central
194868		T.aestivum	Central

194869	√	T.aestivum	Central
194870	√	T.aestivum	Central
194871		T.aestivum	Central
194872		T.aestivum	Central
194873		T.aestivum	Central
194874		T.aestivum	Central
194875		T.aestivum	Central
194876		T.aestivum	Central
194877		T.aestivum	Central
194878		T.aestivum	Central
194879		T.aestivum	Central
194880		T.aestivum	Central
194881		T.aestivum	Central
194882		T.aestivum	Central
194883		T.aestivum	Central
194884		T.aestivum	Central
194885		T.aestivum	Central
194886		T.aestivum	Central
194887		T.aestivum	Central
194888		T.aestivum	Central
194889		T.aestivum	Central
194890		T.aestivum	Central
194892	√	T.aestivum	Central
194893		T.aestivum	Central
194894		T.aestivum	Central
194895	√	T.aestivum	Central
194896		T.aestivum	Central
194897		T.aestivum	Central
194898		T.aestivum	Central
194899		T.aestivum	Central
194900		T.aestivum	Central
194901		T.aestivum	Central
194902		T.aestivum	Central
194903		T.aestivum	Central
194904		T.aestivum	Central
194905		T.aestivum	Central
194906		T.aestivum	Central
194907	√	T.aestivum	Central
194908		T.aestivum	Central
194909	√	T.aestivum	Central
194910		T.aestivum	Central

194911		T.aestivum	Central
194912		T.aestivum	Central
194913		T.aestivum	Central
194914	√	T.aestivum	Central
194915		T.aestivum	Central
194916		T.aestivum	Central
194917		T.aestivum	Central
194918		T.aestivum	Central
194919		T.aestivum	Central
194920		T.aestivum	Central
194921		T.aestivum	Central
194922		T.aestivum	Central
194923	√	T.aestivum	Central
194924		T.aestivum	Central
194925		T.aestivum	Central
194926		T.aestivum	Central
194927		T.aestivum	Central
194928		T.aestivum	Central
194929		T.aestivum	Central
194930		T.aestivum	Central
194931		T.aestivum	Central
194932		T.aestivum	Central
194933		T.aestivum	Central
194934		T.aestivum	Central
194935		T.aestivum	Central
194936	√	T.aestivum	Central
194937		T.aestivum	Central
194938		T.aestivum	Central
194939		T.aestivum	Central
194940	√	T.aestivum	Central
194941		T.aestivum	Central
194942	√	T.aestivum	Central
194943		T.aestivum	Central
194944		T.aestivum	Central
194945		T.aestivum	Central
194946		T.aestivum	Central
194947		T.aestivum	Central
194948		T.aestivum	Central
194949		T.aestivum	Central
194950		T.aestivum	Central
194951		T.aestivum	Central

194952		T.aestivum	Central
194953		T.aestivum	Central
194954		T.aestivum	Central
194955		T.aestivum	Central
194956		T.aestivum	Central
194958		T.aestivum	Central
194959		T.aestivum	Central
194960	√	T.aestivum	Central
194961	√	T.aestivum	Central
194962		T.aestivum	Central
194963		T.aestivum	Central
194964		T.aestivum	Central
194965		T.aestivum	Central
194966		T.aestivum	Central
194967	√	T.aestivum	Central
194968		T.aestivum	Central
194969		T.aestivum	Central
194970		T.aestivum	Central
194971	√	T.aestivum	Central
194972		T.aestivum	Central
194973		T.aestivum	Central
194974		T.aestivum	Central
194975		T.aestivum	Central
194976		T.aestivum	Central
194977		T.aestivum	Central
194978	√	T.aestivum	Central
194981		T.aestivum	Central
194982		T.aestivum	Central
194983	√	T.aestivum	Central
194984		T.aestivum	Central
194985		T.aestivum	Central
194987		T.aestivum	Central
194988		T.aestivum	Central
194989	√	T.aestivum	Central
194990	√	T.aestivum	Central
194991		T.aestivum	Central
194992		T.aestivum	Central
194993		T.aestivum	Central
194994		T.aestivum	Central
194995	√	T.aestivum	Central
194996		T.aestivum	Central

195000	√	T.aestivum	Central
195001	√	T.aestivum	Central
195002		T.aestivum	Central
195003		T.aestivum	Central
195004		T.aestivum	Central
195005		T.aestivum	Central
195006		T.aestivum	Central
195007		T.aestivum	Central
195008		T.aestivum	Central
195009		T.aestivum	Central
195010		T.aestivum	Central
195011		T.aestivum	Central
195012	√	T.aestivum	Central
195013		T.aestivum	Central
195015		T.aestivum	Central
195016		T.aestivum	Central
195017		T.aestivum	Central
195018	√	T.aestivum	Central
195019		T.aestivum	Central
195020		T.aestivum	Central
195021		T.aestivum	Central
195023		T.aestivum	Central
195024		T.aestivum	Central
195025	√	T.aestivum	Central
195026	√	T.aestivum	Central
195027		T.aestivum	Central
195028		T.aestivum	Central
195029		T.aestivum	Central
195030		T.aestivum	Central
195031		T.aestivum	Central
195032	√	T.aestivum	Central
195033		T.aestivum	Central
195034		T.aestivum	Central
195035		T.aestivum	Central
195036		T.aestivum	Central
195037		T.aestivum	Central
195038	√	T.aestivum	Central
195039	√	T.aestivum	Central
195040		T.aestivum	Central
195041		T.aestivum	Central
195042		T.aestivum	Central

195043		T.aestivum	Central
195044		T.aestivum	Central
195045		T.aestivum	Central
195046		T.aestivum	Central
195047		T.aestivum	Central
195048		T.aestivum	Central
195049		T.aestivum	Central
195050	√	T.aestivum	Central
195052		T.aestivum	Central
195057		T.aestivum	Central
195059		T.aestivum	Central
195060		T.aestivum	Central
195061		T.aestivum	Central
195062		T.aestivum	Central
195063		T.aestivum	Central
195064		T.aestivum	Central
195065		T.aestivum	Central
195066		T.aestivum	Central
195067		T.aestivum	Central
195068		T.aestivum	Central
195069		T.aestivum	Central
195070		T.aestivum	Central
195071	√	T.aestivum	Central
195072		T.aestivum	Central
195073	√	T.aestivum	Central
195074		T.aestivum	Central
195075		T.aestivum	Central
195076		T.aestivum	Central
195077		T.aestivum	Central
195078		T.aestivum	Central
195079		T.aestivum	Central
195080		T.aestivum	Central
195081		T.aestivum	Central
195082		T.aestivum	Central
195083		T.aestivum	Central
195084	√	T.aestivum	Central
195085		T.aestivum	Central
195086		T.aestivum	Central
195087		T.aestivum	Central
195088		T.aestivum	Central
195089		T.aestivum	Central

195090	√	T.aestivum	Central
195091		T.aestivum	Central
195092		T.aestivum	Central
195093		T.aestivum	Central
195094		T.aestivum	Central
195095		T.aestivum	Central
195096		T.aestivum	Central
195097		T.aestivum	Central
195098		T.aestivum	Central
195099		T.aestivum	Central
195100		T.aestivum	Central
195101		T.aestivum	Central
195102		T.aestivum	Central
195103		T.aestivum	Central
195105		T.aestivum	Central
195107		T.aestivum	Central
195108		T.aestivum	Central
195109		T.aestivum	Central
195110	√	T.aestivum	Central
195111		T.aestivum	Central
195112	√	T.aestivum	Central
195113	√	T.aestivum	Central
195114		T.aestivum	Central
195115		T.aestivum	Central
195116	√	T.aestivum	Central
195117		T.aestivum	Central
195119		T.aestivum	Central
195120		T.aestivum	Central
195121	√	T.aestivum	Central
195122		T.aestivum	Central
195123		T.aestivum	Central
195124		T.aestivum	Central
195125		T.aestivum	Central
195127		T.aestivum	Central
195128	√	T.aestivum	Central
195129		T.aestivum	Central
195130		T.aestivum	Central
195133		T.aestivum	Central
195134		T.aestivum	Central
195135		T.aestivum	Central
195136		T.aestivum	Central

195138		T.aestivum	Central
195139		T.aestivum	Central
195140		T.aestivum	Central
195141		T.aestivum	Central
195142		T.aestivum	Central
195144		T.aestivum	Central
195145		T.aestivum	Central
195147		T.aestivum	Central
195148		T.aestivum	Central
195149		T.aestivum	Central
195151		T.aestivum	Central
195154		T.aestivum	Central
195155		T.aestivum	Central
195156		T.aestivum	Central
195158		T.aestivum	Central
195160		T.aestivum	Central
195161	√	T.aestivum	Central
195162		T.aestivum	Central
195164	√	T.aestivum	Central
195165		T.aestivum	Central
195166		T.aestivum	Central
195167	√	T.aestivum	Central
195168		T.aestivum	Central
195169	√	T.aestivum	Central
195170		T.aestivum	Central
195171		T.aestivum	Central
195172		T.aestivum	Central
195173		T.aestivum	Central
195174		T.aestivum	Central
195176		T.aestivum	Central
195177		T.aestivum	Central
195178		T.aestivum	Central
195179		T.aestivum	Central
195180		T.aestivum	Central
195182		T.aestivum	Central
195183		T.aestivum	Central
195184		T.aestivum	Central
195185		T.aestivum	Central
195186		T.aestivum	Central
195188		T.aestivum	Central
195190		T.aestivum	Central

195191		T.aestivum	Central
195192		T.aestivum	Central
195193	√	T.aestivum	Central
195196		T.aestivum	Central
195197		T.aestivum	Central
195198		T.aestivum	Central
195199	√	T.aestivum	Central
195200		T.aestivum	Central
195201		T.aestivum	Central
195202		T.aestivum	Central
195203	√	T.aestivum	Central
195204		T.aestivum	Central
195205		T.aestivum	Central
195206		T.aestivum	Central
195207		T.aestivum	Central
195209		T.aestivum	Central
195210		T.aestivum	Central
195211		T.aestivum	Central
195212		T.aestivum	Central
195213		T.aestivum	Central
195215		T.aestivum	Central
195217	√	T.aestivum	Central
195218		T.aestivum	Central
195219	√	T.aestivum	Central
195220		T.aestivum	Central
195221		T.aestivum	Central
195222		T.aestivum	Central
195223	√	T.aestivum	Central
195224	√	T.aestivum	Central
195225		T.aestivum	Central
195226		T.aestivum	Central
195227		T.aestivum	Central
195228		T.aestivum	Central
195229		T.aestivum	Central
195231		T.aestivum	Central
195232		T.aestivum	Central
195233		T.aestivum	Central
195234		T.aestivum	Central
195235		T.aestivum	Central
195236	√	T.aestivum	Central
195237		T.aestivum	Central

195238	√	T.aestivum	Central
195239		T.aestivum	Central
195240		T.aestivum	Central
195241		T.aestivum	Central
195242		T.aestivum	Central
195243		T.aestivum	Central
195248		T.aestivum	Central
195249		T.aestivum	Central
195250		T.aestivum	Central
195251		T.aestivum	Central
195252	√	T.aestivum	Central
195253	√	T.aestivum	Central
195254		T.aestivum	Central
195256		T.aestivum	Central
195257		T.aestivum	Central
195259		T.aestivum	Central
195260		T.aestivum	Central
195262		T.aestivum	Central
195264		T.aestivum	Central
195265		T.aestivum	Central
195266		T.aestivum	Central
195267		T.aestivum	Central
195268		T.aestivum	Central
195269		T.aestivum	Central
195270		T.aestivum	Central
195272		T.aestivum	Central
195273	√	T.aestivum	Central
195274		T.aestivum	Central
195275		T.aestivum	Central
195276		T.aestivum	Central
195277	√	T.aestivum	Central
195279		T.aestivum	Central
195280	√	T.aestivum	Central
195281		T.aestivum	Central
195282		T.aestivum	Central
195283		T.aestivum	Central
195284	√	T.aestivum	Central
195285		T.aestivum	Central
195286		T.aestivum	Central
195287		T.aestivum	Central
195288		T.aestivum	Central

195289		T.aestivum	Central
195290		T.aestivum	Central
195291		T.aestivum	Central
195292		T.aestivum	Central
195293		T.aestivum	Central
195295	√	T.aestivum	Central
195298		T.aestivum	Central
195299		T.aestivum	Central
195300		T.aestivum	Central
195301		T.aestivum	Central
195302	√	T.aestivum	Central
195303		T.aestivum	Central
195304	√	T.aestivum	Central
195306		T.aestivum	Central
195307		T.aestivum	Central
195308		T.aestivum	Central
195309		T.aestivum	Central
195310		T.aestivum	Central
195311		T.aestivum	Central
195312		T.aestivum	Central
195313		T.aestivum	Central
195314		T.aestivum	Central
195315		T.aestivum	Central
195316		T.aestivum	Central
195317		T.aestivum	Central
195318		T.aestivum	Central
195319		T.aestivum	Central
195320		T.aestivum	Central
195322		T.aestivum	Central
195323		T.aestivum	Central
195324		T.aestivum	Central
195325		T.aestivum	Central
195327		T.aestivum	Central
195328		T.aestivum	Central
195329		T.aestivum	Central
195330		T.aestivum	Central
195331		T.aestivum	Central
195333		T.aestivum	Central
195334		T.aestivum	Central
195335		T.aestivum	Central
195336		T.aestivum	Central

195337	√	T.aestivum	Central
195338		T.aestivum	Central
195339		T.aestivum	Central
195340		T.aestivum	Central
195341		T.aestivum	Central
195342		T.aestivum	Central
195343		T.aestivum	Central
195345		T.aestivum	Central
195346		T.aestivum	Central
195348		T.aestivum	Central
195350	√	T.aestivum	Central
195351		T.aestivum	Central
195352		T.aestivum	Central
195353		T.aestivum	Central
195354		T.aestivum	Central
195355	√	T.aestivum	Central
195356		T.aestivum	Central
195357	√	T.aestivum	Central
195358		T.aestivum	Central
195359		T.aestivum	Central
195360		T.aestivum	Central
195361	√	T.aestivum	Central
195362		T.aestivum	Central
195363		T.aestivum	Central
195364		T.aestivum	Central
195365		T.aestivum	Central
195366		T.aestivum	Central
195367		T.aestivum	Central
195368		T.aestivum	Central
195370		T.aestivum	Central
195371		T.aestivum	Central
195372		T.aestivum	Central
195373		T.aestivum	Central
195374		T.aestivum	Central
195375		T.aestivum	Central
195376		T.aestivum	Central
195377		T.aestivum	Central
195378		T.aestivum	Central
195379	√	T.aestivum	Central
195380		T.aestivum	Central
195383		T.aestivum	Central

195384		T.aestivum	Central
195385		T.aestivum	Central
195386		T.aestivum	Central
195387	√	T.aestivum	Central
195388	√	T.aestivum	Central
195395		T.aestivum	Central
195396		T.aestivum	Central
195397	√	T.aestivum	Central
195399		T.aestivum	Central
195401		T.aestivum	Central
195402		T.aestivum	Central
195403		T.aestivum	Central
195404	√	T.aestivum	Central
195405		T.aestivum	Central
195406		T.aestivum	Central
195407		T.aestivum	Central
195408		T.aestivum	Central
195409	√	T.aestivum	Central
195410		T.aestivum	Central
195411		T.aestivum	Central
195412		T.aestivum	Central
195413		T.aestivum	Central
195414		T.aestivum	Central
195415		T.aestivum	Central
195416		T.aestivum	Central
195417		T.aestivum	Central
195418	√	T.aestivum	Central
195419		T.aestivum	Central
195420		T.aestivum	Central
195421		T.aestivum	Central
195422		T.aestivum	Central
195423		T.aestivum	Central
195425	√	T.aestivum	Central
195426		T.aestivum	Central
195427		T.aestivum	Central
195428		T.aestivum	Central
195429	√	T.aestivum	Central
195430		T.aestivum	Central
195431		T.aestivum	Central
195432		T.aestivum	Central
195433		T.aestivum	Central

195434		T.aestivum	Central
195436		T.aestivum	Central
195437		T.aestivum	Central
195438		T.aestivum	Central
195439		T.aestivum	Central
195440		T.aestivum	Central
195441		T.aestivum	Central
195442		T.aestivum	Central
195443		T.aestivum	Central
195444		T.aestivum	Central
195445		T.aestivum	Central
195446		T.aestivum	Central
195447		T.aestivum	Central
195448		T.aestivum	Central
195449		T.aestivum	Central
195451		T.aestivum	Central
195452		T.aestivum	Central
195453		T.aestivum	Central
195454		T.aestivum	Central
195456		T.aestivum	Central
195457	√	T.aestivum	Central
195458		T.aestivum	Central
195460		T.aestivum	Central
195461		T.aestivum	Central
195462	√	T.aestivum	Central
195467		T.aestivum	Central
195468		T.aestivum	Central
195469	√	T.aestivum	Central
195472	√	T.aestivum	Central
195473		T.aestivum	Central
195474		T.aestivum	Central
195475		T.aestivum	Central
195476		T.aestivum	Central
195477		T.aestivum	Central
195478	√	T.aestivum	Central
195479		T.aestivum	Central
195481		T.aestivum	Central
195482		T.aestivum	Central
195483	√	T.aestivum	Central
195484		T.aestivum	Central
195485		T.aestivum	Central

195486		T.aestivum	Central
195487		T.aestivum	Central
195488		T.aestivum	Central
195489		T.aestivum	Central
195490		T.aestivum	Central
195491		T.aestivum	Central
195492		T.aestivum	Central
195494		T.aestivum	Central
195495		T.aestivum	Central
195496		T.aestivum	Central
195497		T.aestivum	Central
195498		T.aestivum	Central
195499		T.aestivum	Central
195500		T.aestivum	Central
195501		T.aestivum	Central
195502		T.aestivum	Central
195503		T.aestivum	Central
195504		T.aestivum	Central
195505		T.aestivum	Central
195506		T.aestivum	Central
195507		T.aestivum	Central
195508		T.aestivum	Central
195509		T.aestivum	Central
195510	√	T.aestivum	Central
195511		T.aestivum	Central
195512		T.aestivum	Central
195513		T.aestivum	Central
195514		T.aestivum	Central
195515		T.aestivum	Central
195516		T.aestivum	Central
195517		T.aestivum	Central
195518		T.aestivum	Central
195519		T.aestivum	Central
195520		T.aestivum	Central
195521		T.aestivum	Central
195522		T.aestivum	Central
195524	√	T.aestivum	Central
195525		T.aestivum	Central
195526		T.aestivum	Central
195527		T.aestivum	Central
195528		T.aestivum	Central

195529		T.aestivum	Central
195530		T.aestivum	Central
195531		T.aestivum	Central
195532		T.aestivum	Central
195533		T.aestivum	Central
195534		T.aestivum	Central
195535		T.aestivum	Central
195536		T.aestivum	Central
195537		T.aestivum	Central
195538		T.aestivum	Central
195539		T.aestivum	Central
195540		T.aestivum	Central
195541		T.aestivum	Central
195542		T.aestivum	Central
195543	√	T.aestivum	Central
195544		T.aestivum	Central
195545		T.aestivum	Central
195546		T.aestivum	Central
195547		T.aestivum	Central
195548	√	T.aestivum	Central
195549		T.aestivum	Central
195550		T.aestivum	Central
195551		T.aestivum	Central
195552		T.aestivum	Central
195553		T.aestivum	Central
195554		T.aestivum	Central
195555		T.aestivum	Central
195556		T.aestivum	Central
195557		T.aestivum	Central
195558		T.aestivum	Central
195559		T.aestivum	Central
195560		T.aestivum	Central
195561		T.aestivum	Central
195562		T.aestivum	Central
195564	√	T.aestivum	Central
195565		T.aestivum	Central
195566		T.aestivum	Central
195567	√	T.aestivum	Central
195568		T.aestivum	Central
195569		T.aestivum	Central
195570	√	T.aestivum	Central

195571		T.aestivum	Central
195572		T.aestivum	Central
195573		T.aestivum	Central
195574		T.aestivum	Central
195575	√	T.aestivum	Central
195576		T.aestivum	Central
195577		T.aestivum	Central
195578		T.aestivum	Central
195579		T.aestivum	Central
195580	√	T.aestivum	Central
195581	√	T.aestivum	Central
195582		T.aestivum	Central
195583		T.aestivum	Central
195584		T.aestivum	Central
195585		T.aestivum	Central
195586		T.aestivum	Central
195587		T.aestivum	Central
195588		T.aestivum	Central
195589		T.aestivum	Central
195590		T.aestivum	Central
195593		T.aestivum	Central
195594		T.aestivum	Central
195595	√	T.aestivum	Central
195596		T.aestivum	Central
195597		T.aestivum	Central
195598		T.aestivum	Central
195599		T.aestivum	Central
195601		T.aestivum	Central
195602		T.aestivum	Central
195604		T.aestivum	Central
195605		T.aestivum	Central
195606		T.aestivum	Central
195607	√	T.aestivum	Central
195608		T.aestivum	Central
195609		T.aestivum	Central
195611	√	T.aestivum	Central
195612		T.aestivum	Central
195613		T.aestivum	Central
195614		T.aestivum	Central
195615		T.aestivum	Central
195616		T.aestivum	Central

195617		T.aestivum	Central
195618		T.aestivum	Central
195619	√	T.aestivum	Central
195620		T.aestivum	Central
195621		T.aestivum	Central
195622	√	T.aestivum	Central
195623		T.aestivum	Central
195624		T.aestivum	Central
195626		T.aestivum	Central
195627		T.aestivum	Central
195628		T.aestivum	Central
195629		T.aestivum	Central
195630		T.aestivum	Central
195632	√	T.aestivum	Central
195633		T.aestivum	Central
195634		T.aestivum	Central
195635		T.aestivum	Central
195636		T.aestivum	Central
195637		T.aestivum	Central
195638		T.aestivum	Central
195639		T.aestivum	Central
195640	√	T.aestivum	Central
195642		T.aestivum	Central
195643		T.aestivum	Central
195644		T.aestivum	Central
195645		T.aestivum	Central
195646		T.aestivum	Central
195647		T.aestivum	Central
195648		T.aestivum	Central
195649		T.aestivum	Central
195650		T.aestivum	Central
195652	√	T.aestivum	Central
195653		T.aestivum	Central
195654		T.aestivum	Central
195655		T.aestivum	Central
195656		T.aestivum	Central
195657		T.aestivum	Central
195658		T.aestivum	Central
195659		T.aestivum	Central
195660		T.aestivum	Central
195661		T.aestivum	Central

195662	√	T.aestivum	Central
195663		T.aestivum	Central
195664		T.aestivum	Central
195666		T.aestivum	Central
195668		T.aestivum	Central
195669		T.aestivum	Central
195670		T.aestivum	Central
195671		T.aestivum	Central
195672		T.aestivum	Central
195675	√	T.aestivum	Central
195676		T.aestivum	Central
195677		T.aestivum	Central
195678		T.aestivum	Central
195679	√	T.aestivum	Central
195681		T.aestivum	Central
195682		T.aestivum	Central
195683		T.aestivum	Central
195684		T.aestivum	Central
195685		T.aestivum	Central
195686		T.aestivum	Central
195687		T.aestivum	Central
195688		T.aestivum	Central
195689		T.aestivum	Central
195690		T.aestivum	Central
195691		T.aestivum	Central
195692		T.aestivum	Central
195693		T.aestivum	Central
195694		T.aestivum	Central
195695		T.aestivum	Central
195696		T.aestivum	Central
195697	√	T.aestivum	Central
195698		T.aestivum	Central
195699		T.aestivum	Central
195701		T.aestivum	Central
195702		T.aestivum	Central
195704		T.aestivum	Central
195705		T.aestivum	Central
195707		T.aestivum	Central
195708	√	T.aestivum	Central
195709		T.aestivum	Central
195711		T.aestivum	Central

195712	√	T.aestivum	Central
195713		T.aestivum	Central
195714		T.aestivum	Central
195715		T.aestivum	Central
195716		T.aestivum	Central
195717		T.aestivum	Central
195719		T.aestivum	Central
195720		T.aestivum	Central
195722		T.aestivum	Central
195723		T.aestivum	Central
195724		T.aestivum	Central
195725		T.aestivum	Central
195726		T.aestivum	Central
195727		T.aestivum	Central
195729		T.aestivum	Central
195730		T.aestivum	Central
195733		T.aestivum	Central
195734		T.aestivum	Central
195735	√	T.aestivum	Central
195736		T.aestivum	Central
195737		T.aestivum	Central
195738		T.aestivum	Central
195739		T.aestivum	Central
195740		T.aestivum	Central
195741		T.aestivum	Central
195742		T.aestivum	Central
195744		T.aestivum	Central
195745		T.aestivum	Central
195748		T.aestivum	Central
195750		T.aestivum	Central
195752		T.aestivum	Central
195754		T.aestivum	Central
195755		T.aestivum	Central
195756		T.aestivum	Central
195757		T.aestivum	Central
195758		T.aestivum	Central
195759		T.aestivum	Central
195760		T.aestivum	Central
195761		T.aestivum	Central
195766	√	T.aestivum	Central
195767		T.aestivum	Central

195769		T.aestivum	Central
195772		T.aestivum	Central
195773		T.aestivum	Central
195774		T.aestivum	Central
195775		T.aestivum	Central
195776	√	T.aestivum	Central
195777	√	T.aestivum	Central
195778		T.aestivum	Central
195779		T.aestivum	Central
195780		T.aestivum	Central
195781		T.aestivum	Central
195782		T.aestivum	Central
195783		T.aestivum	Central
195784		T.aestivum	Central
195785		t.aestivum	Central
195786		T.aestivum	Central
195787		T.aestivum	Central
195788	√	T.aestivum	Central
195789		T.aestivum	Central
195790		T.aestivum	Central
195791		T.aestivum	Central
195792		T.aestivum	Central
195793		T.aestivum	Central
195795		T.aestivum	Central
195796		T.aestivum	Central
195797		T.aestivum	Central
195798		T.aestivum	Central
195799		T.aestivum	Central
195800		T.aestivum	Central
195801		T.aestivum	Central
195802		T.aestivum	Central
195803		T.aestivum	Central
195804		T.aestivum	Central
195805		T.aestivum	Central
195806	√	T.aestivum	Central
195807		T.aestivum	Central
195808		T.aestivum	Central
195809	√	T.aestivum	Central
195810	√	T.aestivum	Central
195811		T.aestivum	Central
195812		T.aestivum	Central

195813		T.aestivum	Central
195815		T.aestivum	Central
195816		T.aestivum	Central
195817		T.aestivum	Central
195819		T.aestivum	Central
195820		T.aestivum	Central
195822		T.aestivum	Central
195824	√	T.aestivum	Central
195829		T.aestivum	Central
195837		T.aestivum	Central
195838		T.aestivum	Central
195839		T.aestivum	Central
195840		T.aestivum	Central
195841		T.aestivum	Central
195842	√	T.aestivum	Central
195844		T.aestivum	Central
195846		T.aestivum	Central
195847		T.aestivum	Central
195848		T.aestivum	Central
195849		T.aestivum	Central
195850		T.aestivum	Central
195851		T.aestivum	Central
195852		T.aestivum	Central
195853		T.aestivum	Central
195854		T.aestivum	Central
195855		T.aestivum	Central
195856		T.aestivum	Central
195857		T.aestivum	Central
195858		T.aestivum	Central
195859	√	T.aestivum	Central
195860		T.aestivum	Central
195861		T.aestivum	Central
195862		T.aestivum	Central
195864		T.aestivum	Central
195866		T.aestivum	Central
195868		T.aestivum	Central
195870		T.aestivum	Central
195871		T.aestivum	Central
195872		T.aestivum	Central
195876		T.aestivum	Central
195877		T.aestivum	Central

195878		T.aestivum	Central
195879		T.aestivum	Central
195880		T.aestivum	Central
195884	√	T.aestivum	Central
195886		T.aestivum	Central
195888		T.aestivum	Central
195889	√	T.aestivum	Central
195890		T.aestivum	Central
195891		T.aestivum	Central
195892	√	T.aestivum	Central
195893		T.aestivum	Central
195894	√	T.aestivum	Central
195895		T.aestivum	Central
195896		T.aestivum	Central
195897	√	T.aestivum	Central
195898		T.aestivum	Central
195899		T.aestivum	Central
195900		T.aestivum	Central
195901		T.aestivum	Central
195902		T.aestivum	Central
195903		T.aestivum	Central
195904		T.aestivum	Central
195905		T.aestivum	Central
195906		T.aestivum	Central
195907		T.aestivum	Central
195908		T.aestivum	Central
195909		T.aestivum	Central
195910	√	T.aestivum	Central
195911		T.aestivum	Central
195913		T.aestivum	Central
195914	√	T.aestivum	Central
195915		T.aestivum	Central
195916		T.aestivum	Central
195918		T.aestivum	Central
195919		T.aestivum	Central
195920		T.aestivum	Central
195922		T.aestivum	Central
195923		T.aestivum	Central
195924		T.aestivum	Central
195925		T.aestivum	Central
195926	√	T.aestivum	Central

195927		T.aestivum	Central
195928		T.aestivum	Central
195929		T.aestivum	Central
195930		T.aestivum	Central
195931		T.aestivum	Central
195932		T.aestivum	Central
195933		T.aestivum	Central
195934		T.aestivum	Central
195935		T.aestivum	Central
195936		T.aestivum	Central
195937		T.aestivum	Central
195938		T.aestivum	Central
195939		T.aestivum	Central
195940	√	T.aestivum	Central
195941		T.aestivum	Central
195942		T.aestivum	Central
195943	√	T.aestivum	Central
195944		T.aestivum	Central
195945		T.aestivum	Central
195947		T.aestivum	Central
195948		T.aestivum	Central
195949	√	T.aestivum	Central
195950		T.aestivum	Central
195951		T.aestivum	Central
195952		T.aestivum	Central
195953	√	T.aestivum	Central
195954		T.aestivum	Central
195955		T.aestivum	Central
195956	√	T.aestivum	Central
195957		T.aestivum	Central
195958		T.aestivum	Central
195959		T.aestivum	Central
195960		T.aestivum	Central
195961		T.aestivum	Central
195962		T.aestivum	Central
195963		T.aestivum	Central
195964		T.aestivum	Central
195965		T.aestivum	Central
195966		T.aestivum	Central
195967		T.aestivum	Central
195968		T.aestivum	Central

195969		T.aestivum	Central
195970		T.aestivum	Central
195971		T.aestivum	Central
195972		T.aestivum	Central
195973		T.aestivum	Central
195974		T.aestivum	Central
195976		T.aestivum	Central
195977	√	T.aestivum	Central
195978		T.aestivum	Central
195979		T.aestivum	Central
195980	√	T.aestivum	Central
195981		T.aestivum	Central
195982	√	T.aestivum	Central
195983		T.aestivum	Central
195984		T.aestivum	Central
195985		T.aestivum	Central
195986		T.aestivum	Central
195987		T.aestivum	Central
195990	√	T.aestivum	Central
195999		T.aestivum	Central
196000		T.aestivum	Central
196002		T.aestivum	Central
196003		T.aestivum	Central
196004		T.aestivum	Central
196005		T.aestivum	Central
196007		T.aestivum	Central
196008		T.aestivum	Central
196009		T.aestivum	Central
196010	√	T.aestivum	Central
196011	√	T.aestivum	Central
196012	√	T.aestivum	Central
196013		T.aestivum	Central
196014		T.aestivum	Central
196016		T.aestivum	Central
196017		T.aestivum	Central
196018		T.aestivum	Central
196019		T.aestivum	Central
196020		T.aestivum	Central
196021	√	T.aestivum	Central
196022	√	T.aestivum	Central
196023		T.aestivum	Central

196024		T.aestivum	Central
196025		T.aestivum	Central
196026		T.aestivum	Central
196028		T.aestivum	Central
196029		T.aestivum	Central
196030		T.aestivum	Central
196036		T.aestivum	Central
196038		T.aestivum	Central
196039		T.aestivum	Central
196040		T.aestivum	Central
196041		T.aestivum	Central
196044		T.aestivum	Central
196046	√	T.aestivum	Central
196047		T.aestivum	Central
196049		T.aestivum	Central
196050		T.aestivum	Central
196051	√	T.aestivum	Central
196052		T.aestivum	Central
196053	√	T.aestivum	Central
196054		T.aestivum	Central
196055		T.aestivum	Central
196056		T.aestivum	Central
196057		T.aestivum	Central
196058	√	T.aestivum	Central
196059		T.aestivum	Central
196060		T.aestivum	Central
196061		T.aestivum	Central
196062		T.aestivum	Central
196063		T.aestivum	Central
196064	√	T.aestivum	Central
196065		T.aestivum	Central
196066		T.aestivum	Central
196067		T.aestivum	Central
196068		T.aestivum	Central
196069		T.aestivum	Central
196070		T.aestivum	Central
196072	√	T.aestivum	Central
196073	√	T.aestivum	Central
196074		T.aestivum	Central
196078		T.aestivum	Central
196079		T.aestivum	Central

196080		T.aestivum	Central
196081		T.aestivum	Central
196082		T.aestivum	Central
196083		T.aestivum	Central
196084		T.aestivum	Central
196085		T.aestivum	Central
196086		T.aestivum	Central
196087	√	T.aestivum	Central
196088		T.aestivum	Central
196089		T.aestivum	Central
196090	√	T.aestivum	Central
196091	√	T.aestivum	Central
196092		T.aestivum	Central
196094		T.aestivum	Central
196097		T.aestivum	Central
196099		T.aestivum	Central
196104		T.aestivum	Central
196105		T.aestivum	Central
196107		T.aestivum	Central
196108		T.aestivum	Central
196109		T.aestivum	Central
196110		T.aestivum	Central
196113	√	T.aestivum	Central
206776		T.aestivum	Central
206777		T.aestivum	Central
206778		T.aestivum	Central
206779	√	T.aestivum	Central
206780		T.aestivum	Central
206781		T.aestivum	Central
206782		T.aestivum	Central
206783		T.aestivum	Central
206784		T.aestivum	Central
206785	√	T.aestivum	Central
206786		T.aestivum	Central
206788		T.aestivum	Central
206790		T.aestivum	Central
206791		T.aestivum	Central
206792		T.aestivum	Central
206793		T.aestivum	Central
206794		T.aestivum	Central
206795		T.aestivum	Central

206796		T.aestivum	Central
206797		T.aestivum	Central
206798		T.aestivum	Central
206799		T.aestivum	Central
206800		T.aestivum	Central
206801		T.aestivum	Central
206802		T.aestivum	Central
206803		T.aestivum	Central
206804	√	T.aestivum	Central
206805	√	T.aestivum	Central
206807		T.aestivum	Central
206809		T.aestivum	Central
206810	√	T.aestivum	Central
206811		T.aestivum	Central
206812		T.aestivum	Central
206813		T.aestivum	Central
206815		T.aestivum	Central
206816		T.aestivum	Central
206817		T.aestivum	Central
206818		T.aestivum	Central
206819	√	T.aestivum	Central
206821	√	T.aestivum	Central
206822	√	T.aestivum	Central
206824		T.aestivum	Central
206826		T.aestivum	Central
206827	√	T.aestivum	Central
206828		T.aestivum	Central
206829		T.aestivum	Central
206830		T.aestivum	Central
206831	√	T.aestivum	Central
206832		T.aestivum	Central
206836		T.aestivum	Central
206837		T.aestivum	Central
206838		T.aestivum	Central
206846		T.aestivum	Central
206851		T.aestivum	Central
206865	√	T.aestivum	Central
206868		T.aestivum	Central
206869		T.aestivum	Central
206870		T.aestivum	Central
206871		T.aestivum	Central

206872	√	T.aestivum	Central
206873		T.aestivum	Central
206874		T.aestivum	Central
206875		T.aestivum	Central
206876		T.aestivum	Central
206877		T.aestivum	Central
206878		T.aestivum	Central
206879		T.aestivum	Central
206880		T.aestivum	Central
206881		T.aestivum	Central
206882		T.aestivum	Central
206883		T.aestivum	Central
206884		T.aestivum	Central
206885		T.aestivum	Central
206886		T.aestivum	Central
206887		T.aestivum	Central
206888	√	T.aestivum	Central
206889		T.aestivum	Central
206890		T.aestivum	Central
206891		T.aestivum	Central
206892		T.aestivum	Central
206893		T.aestivum	Central
206894		T.aestivum	Central
206895		T.aestivum	Central
206896	√	T.aestivum	Central
206897		T.aestivum	Central
206898		T.aestivum	Central
206899		T.aestivum	Central
206900		T.aestivum	Central
206901		T.aestivum	Central
206902		T.aestivum	Central
206903		T.aestivum	Central
206904		T.aestivum	Central
206905		T.aestivum	Central
206906		T.aestivum	Central
206907		T.aestivum	Central
206908		T.aestivum	Central
206909		T.aestivum	Central
206910		T.aestivum	Central
206912		T.aestivum	Central
206914	√	T.aestivum	Central

206915	√	T.aestivum	Central
206916		T.aestivum	Central
206917		T.aestivum	Central
206918		T.aestivum	Central
206919		T.aestivum	Central
206920		T.aestivum	Central
206921		T.aestivum	Central
206922		T.aestivum	Central
206923	√	T.aestivum	Central
206924		T.aestivum	Central
206925		T.aestivum	Central
206926		T.aestivum	Central
206927		T.aestivum	Central
206929		T.aestivum	Central
206930		T.aestivum	Central
206931		T.aestivum	Central
206932		T.aestivum	Central
206933		T.aestivum	Central
206934		T.aestivum	Central
206935		T.aestivum	Central
206936		T.aestivum	Central
206937		T.aestivum	Central
206938		T.aestivum	Central
206939		T.aestivum	Central
206940		T.aestivum	Central
206941	√	T.aestivum	Central
206942		T.aestivum	Central
206943		T.aestivum	Central
206944		T.aestivum	Central
206945		T.aestivum	Central
206947		T.aestivum	Central
206948		T.aestivum	Central
206950	√	T.aestivum	Central
206951	√	T.aestivum	Central
206953		T.aestivum	Central
206954		T.aestivum	Central
206955		T.aestivum	Central
206956		T.aestivum	Central
206957		T.aestivum	Central
206958		T.aestivum	Central
206959		T.aestivum	Central

206960		T.aestivum	Central
206961		T.aestivum	Central
206962		T.aestivum	Central
206963		T.aestivum	Central
206965	√	T.aestivum	Central
206966		T.aestivum	Central
206967		T.aestivum	Central
206968		T.aestivum	Central
206969		T.aestivum	Central
206970		T.aestivum	Central
206971		T.aestivum	Central
206972		T.aestivum	Central
206973		T.aestivum	Central
206974		T.aestivum	Central
206975		T.aestivum	Central
206976		T.aestivum	Central
206977		T.aestivum	Central
206978	√	T.aestivum	Central
206979		T.aestivum	Central
206981		T.aestivum	Central
206982		T.aestivum	Central
206983		T.aestivum	Central
206984		T.aestivum	Central
206985		T.aestivum	Central
206986		T.aestivum	Central
206987		T.aestivum	Central
206988		T.aestivum	Central
206989		T.aestivum	Central
206990		T.aestivum	Central
206991		T.aestivum	Central
206992		T.aestivum	Central
206993		T.aestivum	Central
206994	√	T.aestivum	Central
206995		T.aestivum	Central
206996		T.aestivum	Central
206997		T.aestivum	Central
206998		T.aestivum	Central
206999		T.aestivum	Central
207000		T.aestivum	Central
207001		T.aestivum	Central
207002		T.aestivum	Central

207003		T.aestivum	Central
207004		T.aestivum	Central
207005		T.aestivum	Central
207006		T.aestivum	Central
207007		T.aestivum	Central
207008		T.aestivum	Central
207009		T.aestivum	Central
207010		T.aestivum	Central
207011		T.aestivum	Central
207012		T.aestivum	Central
207013		T.aestivum	Central
207014		T.aestivum	Central
207015		T.aestivum	Central
207016		T.aestivum	Central
207017		T.aestivum	Central
207018		T.aestivum	Central
207019		T.aestivum	Central
207020		T.aestivum	Central
207022		T.aestivum	Central
207023		T.aestivum	Central
207024		T.aestivum	Central
207025	√	T.aestivum	Central
207026		T.aestivum	Central
207027		T.aestivum	Central
207028		T.aestivum	Central
207029		T.aestivum	Central
207030		T.aestivum	Central
207031		T.aestivum	Central
207032	√	T.aestivum	Central
207033		T.aestivum	Central
207034		T.aestivum	Central
207035		T.aestivum	Central
207036	√	T.aestivum	Central
207037		T.aestivum	Central
207038		T.aestivum	Central
207039		T.aestivum	Central
207040		T.aestivum	Central
207041		T.aestivum	Central
207042		T.aestivum	Central
207043		T.aestivum	Central
207044		T.aestivum	Central

207045		T.aestivum	Central
207046		T.aestivum	Central
207047		T.aestivum	Central
207048		T.aestivum	Central
207049		T.aestivum	Central
207050		T.aestivum	Central
207051		T.aestivum	Central
207052		T.aestivum	Central
207053		T.aestivum	Central
207054		T.aestivum	Central
207055		T.aestivum	Central
207056		T.aestivum	Central
207057		T.aestivum	Central
207058		T.aestivum	Central
207059		T.aestivum	Central
207060		T.aestivum	Central
207061		T.aestivum	Central
207062		T.aestivum	Central
207063	√	T.aestivum	Central
207066		T.aestivum	Central
207067	√	T.aestivum	Central
207068		T.aestivum	Central
207069		T.aestivum	Central
207072		T.aestivum	Central
207073		T.aestivum	Central
207074		T.aestivum	Central
207075	√	T.aestivum	Central
207076		T.aestivum	Central
207077	√	T.aestivum	Central
207078		T.aestivum	Central
207079		T.aestivum	Central
207080	√	T.aestivum	Central
207081		T.aestivum	Central
207082		T.aestivum	Central
207083		T.aestivum	Central
207084		T.aestivum	Central
207085		T.aestivum	Central
207087		T.aestivum	Central
207088		T.aestivum	Central
207089		T.aestivum	Central
207090		T.aestivum	Central

207091		T.aestivum	Central
207092		T.aestivum	Central
207093		T.aestivum	Central
207094		T.aestivum	Central
207095		T.aestivum	Central
207096		T.aestivum	Central
207097		T.aestivum	Central
207098		T.aestivum	Central
207099		T.aestivum	Central
207100		T.aestivum	Central
207101		T.aestivum	Central
207102		T.aestivum	Central
207106		T.aestivum	Central
207107		T.aestivum	Central
207108		T.aestivum	Central
207109	√	T.aestivum	Central
207110	√	T.aestivum	Central
207111		T.aestivum	Central
207112		T.aestivum	Central
207113		T.aestivum	Central
207114		T.aestivum	Central
207115		T.aestivum	Central
207116		T.aestivum	Central
217490		T.aestivum	Central
217492		T.aestivum	Central
217493		T.aestivum	Central
217495		T.aestivum	Central
217496		T.aestivum	Central
217499		T.aestivum	Central
217500	√	T.aestivum	Central
217501		T.aestivum	Central
217502	√	T.aestivum	Central
217503		T.aestivum	Central
217507		T.aestivum	Central
217508	√	T.aestivum	Central
217510		T.aestivum	Central
217511		T.aestivum	Central
217512		T.aestivum	Central
217514		T.aestivum	Central
217515		T.aestivum	Central
217516	√	T.aestivum	Central

217517		T.aestivum	Central
217524		T.aestivum	Central
217525		T.aestivum	Central
217526		T.aestivum	Central
217528		T.aestivum	Central
217529		T.aestivum	Central
217530		T.aestivum	Central
217532		T.aestivum	Central
217533		T.aestivum	Central
217534		T.aestivum	Central
217536		T.aestivum	Central
217537		T.aestivum	Central
217539		T.aestivum	Central
217540	√	T.aestivum	Central
217541		T.aestivum	Central
217542	√	T.aestivum	Central
217551		T.aestivum	Central
217552		T.aestivum	Central
217553		T.aestivum	Central
217554		T.aestivum	Central
217556		T.aestivum	Central
221528		T.aestivum	Central
221529		T.aestivum	Central
221530		T.aestivum	Central
221531		T.aestivum	Central
221532		T.aestivum	Central
221533		T.aestivum	Central
221534		T.aestivum	Central
221535		T.aestivum	Central
221537		T.aestivum	Central
221538	√	T.aestivum	Central
221539	√	T.aestivum	Central
221540		T.aestivum	Central
221541		T.aestivum	Central
221542		T.aestivum	Central
221543		T.aestivum	Central
221545		T.aestivum	Central
221546		T.aestivum	Central
221547		T.aestivum	Central
221548		T.aestivum	Central
221549		T.aestivum	Central

221550		T.aestivum	Central
221551	√	T.aestivum	Central
221552		T.aestivum	Central
221553		T.aestivum	Central
221554		T.aestivum	Central
221555	√	T.aestivum	Central
221556		T.aestivum	Central
221557	√	T.aestivum	Central
221558		T.aestivum	Central
221559	√	T.aestivum	Central
221560		T.aestivum	Central
221563		T.aestivum	Central
221564	√	T.aestivum	Central
221565		T.aestivum	Central
221566		T.aestivum	Central
221567		T.aestivum	Central
221568		T.aestivum	Central
221569		T.aestivum	Central
221570		T.aestivum	Central
221571	√	T.aestivum	Central
221572	√	T.aestivum	Central
221573	√	T.aestivum	Central
221574		T.aestivum	Central
221575		T.aestivum	Central
221576	√	T.aestivum	Central
221577		T.aestivum	Central
221578	√	T.aestivum	Central
221579		T.aestivum	Central
221580		T.aestivum	Central
221581		T.aestivum	Central
221582		T.aestivum	Central
221583	√	T.aestivum	Central
221584		T.aestivum	Central
221585		T.aestivum	Central
221588		T.aestivum	Central
221589		T.aestivum	Central
221590		T.aestivum	Central
221591		T.aestivum	Central
221592		T.aestivum	Central
221593		T.aestivum	Central
221594	√	T.aestivum	Central

221595		T.aestivum	Central
221599		T.aestivum	Central
221600		T.aestivum	Central
221601		T.aestivum	Central
221602		T.aestivum	Central
221603		T.aestivum	Central
221604		T.aestivum	Central
221605		T.aestivum	Central
221606		T.aestivum	Central
221607		T.aestivum	Central
221608	√	T.aestivum	Central
221609	√	T.aestivum	Central
221610		T.aestivum	Central
221611		T.aestivum	Central
221612		T.aestivum	Central
221613		T.aestivum	Central
221614	√	T.aestivum	Central
221615		T.aestivum	Central
221616		T.aestivum	Central
221617		T.aestivum	Central
221618		T.aestivum	Central
221620		T.aestivum	Central
221621	√	T.aestivum	Central
221622		T.aestivum	Central
221623		T.aestivum	Central
221624	√	T.aestivum	Central
221625		T.aestivum	Central
221626		T.aestivum	Central
221627		T.aestivum	Central
221628		T.aestivum	Central
221629		T.aestivum	Central
221630		T.aestivum	Central
221631		T.aestivum	Central
221632		T.aestivum	Central
221633		T.aestivum	Central
221634		T.aestivum	Central
221635	√	T.aestivum	Central
221636		T.aestivum	Central
221637		T.aestivum	Central
221638		T.aestivum	Central
221639		T.aestivum	Central

221640		T.aestivum	Central
221641		T.aestivum	Central
221642		T.aestivum	Central
221643		T.aestivum	Central
221644		T.aestivum	Central
221645		T.aestivum	Central
221646		T.aestivum	Central
221647		T.aestivum	Central
221648		T.aestivum	Central
221649		T.aestivum	Central
221650		T.aestivum	Central
221651	√	T.aestivum	Central
221654		T.aestivum	Central
221655		T.aestivum	Central
221656		T.aestivum	Central
221657		T.aestivum	Central
221658		T.aestivum	Central
221659	√	T.aestivum	Central
221660		T.aestivum	Central
221661		T.aestivum	Central
221662		T.aestivum	Central
221663		T.aestivum	Central
221664		T.aestivum	Central
221665		T.aestivum	Central
221666		T.aestivum	Central
221667		T.aestivum	Central
221668		T.aestivum	Central
221669		T.aestivum	Central
221670		T.aestivum	Central
221671		T.aestivum	Central
221672		T.aestivum	Central
221673		T.aestivum	Central
221674		T.aestivum	Central
221675		T.aestivum	Central
221676		T.aestivum	Central
221677		T.aestivum	Central
221678	√	T.aestivum	Central
221679	√	T.aestivum	Central
221680		T.aestivum	Central
221681	√	T.aestivum	Central
221682		T.aestivum	Central

221683		T.aestivum	Central
221684		T.aestivum	Central
221685		T.aestivum	Central
221686		T.aestivum	Central
221687		T.aestivum	Central
221688		T.aestivum	Central
221689		T.aestivum	Central
221690		T.aestivum	Central
221691		T.aestivum	Central
221692		T.aestivum	Central
221693		T.aestivum	Central
221694		T.aestivum	Central
221695		T.aestivum	Central
221696		T.aestivum	Central
221697		T.aestivum	Central
221762	√	T.aestivum	Central
221763		T.aestivum	Central
221764		T.aestivum	Central
221765	√	T.aestivum	Central
221766		T.aestivum	Central
221767		T.aestivum	Central
221768		T.aestivum	Central
221769		T.aestivum	Central
221770	√	T.aestivum	Central
221771	√	T.aestivum	Central
221772		T.aestivum	Central
221773		T.aestivum	Central
221774		T.aestivum	Central
221775		T.aestivum	Central
221776		T.aestivum	Central
221777	√	T.aestivum	Central
221778		T.aestivum	Central
221779	√	T.aestivum	Central
221780		T.aestivum	Central
221781		T.aestivum	Central
221782		T.aestivum	Central
221783		T.aestivum	Central
221784		T.aestivum	Central
221785		T.aestivum	Central
221786		T.aestivum	Central
221787	√	T.aestivum	Central

221788		T.aestivum	Central
221789		T.aestivum	Central
221790		T.aestivum	Central
221791		T.aestivum	Central
221793		T.aestivum	Central
221794		T.aestivum	Central
221795		T.aestivum	Central
221796		T.aestivum	Central
221797	√	T.aestivum	Central
221798		T.aestivum	Central
221799		T.aestivum	Central
221800		T.aestivum	Central
221801		T.aestivum	Central
221802	√	T.aestivum	Central
221803	√	T.aestivum	Central
221804		T.aestivum	Central
221805		T.aestivum	Central
221806		T.aestivum	Central
221809	√	T.aestivum	Central
221810		T.aestivum	Central
221811		T.aestivum	Central
221812		T.aestivum	Central
221813		T.aestivum	Central
221814	√	T.aestivum	Central
221815	√	T.aestivum	Central
221816		T.aestivum	Central
221817		T.aestivum	Central
221818		T.aestivum	Central
221819	√	T.aestivum	Central
221820		T.aestivum	Central
221821	√	T.aestivum	Central
221822		T.aestivum	Central
221823		T.aestivum	Central
221824	√	T.aestivum	Central
221825		T.aestivum	Central
221826		T.aestivum	Central
221827		T.aestivum	Central
221829		T.aestivum	Central
221830		T.aestivum	Central
221831		T.aestivum	Central
221832		T.aestivum	Central

221833	√	T.aestivum	Central
221834	√	T.aestivum	Central
221835		T.aestivum	Central
221836		T.aestivum	Central
221837		T.aestivum	Central
221839		T.aestivum	Central
221840		T.aestivum	Central
221841		T.aestivum	Central
221842		T.aestivum	Central
221843		T.aestivum	Central
221844	√	T.aestivum	Central
221845		T.aestivum	Central
221846		T.aestivum	Central
221848		T.aestivum	Central
221849		T.aestivum	Central
221850		T.aestivum	Central
221851		T.aestivum	Central
221852	√	T.aestivum	Central
221853	√	T.aestivum	Central
221916		T.aestivum	Central
221917		T.aestivum	Central
221919	√	T.aestivum	Central
221920		T.aestivum	Central
221921		T.aestivum	Central
221922		T.aestivum	Central
221923		T.aestivum	Central
221924		T.aestivum	Central
221925		T.aestivum	Central
221926		T.aestivum	Central
221927		T.aestivum	Central
221928		T.aestivum	Central
221929		T.aestivum	Central
221931		T.aestivum	Central
221932		T.aestivum	Central
221933		T.aestivum	Central
221934	√	T.aestivum	Central
221935		T.aestivum	Central
221937		T.aestivum	Central
221938		T.aestivum	Central
221940		T.aestivum	Central
221941	√	T.aestivum	Central

221942		T.aestivum	Central
221943		T.aestivum	Central
221944		T.aestivum	Central
221945		T.aestivum	Central
221946		T.aestivum	Central
221947		T.aestivum	Central
221948	√	T.aestivum	Central
221949	√	T.aestivum	Central
221950		T.aestivum	Central
221952		T.aestivum	Central
221953		T.aestivum	Central
221954		T.aestivum	Central
221955	√	T.aestivum	Central
221958		T.aestivum	Central
221959		T.aestivum	Central
221960	√	T.aestivum	Central
221961	√	T.aestivum	Central
221962		T.aestivum	Central
221963		T.aestivum	Central
221964		T.aestivum	Central
221965		T.aestivum	Central
221966		T.aestivum	Central
221967	√	T.aestivum	Central
221969		T.aestivum	Central
223713		T.aestivum	North
223714		T.aestivum	North
223715		T.aestivum	North
223716		T.aestivum	North
223718		T.aestivum	North
223719	√	T.aestivum	North
223720		T.aestivum	North
223721		T.aestivum	North
223722		T.aestivum	North
223723	√	T.aestivum	North
223730		T.aestivum	North
223731		T.aestivum	North
223732		T.aestivum	North
223733		T.aestivum	North
223734	√	T.aestivum	North
223736		T.aestivum	North
223737		T.aestivum	North

223738		T.aestivum	North
223739	√	T.aestivum	North
223741		T.aestivum	North
223744		T.aestivum	North
223745		T.aestivum	North
223746		T.aestivum	North
223747		T.aestivum	North
223748		T.aestivum	North
223749		T.aestivum	North
223750		T.aestivum	North
223751		T.aestivum	North
223752		T.aestivum	North
223753		T.aestivum	North
223755	√	T.aestivum	North
223756		T.aestivum	North
223757		T.aestivum	North
223758		T.aestivum	North
223759	√	T.aestivum	North
223760		T.aestivum	North
223761		T.aestivum	North
223762		T.aestivum	North
223763		T.aestivum	North
223764		T.aestivum	North
223765		T.aestivum	North
223766		T.aestivum	North
223767	√	T.aestivum	North
223768		T.aestivum	North
223769		T.aestivum	North
223770		T.aestivum	North
223771		T.aestivum	North
223772		T.aestivum	North
223773		T.aestivum	North
223774	√	T.aestivum	North
223775		T.aestivum	North
223776		T.aestivum	North
223777		T.aestivum	North
223778		T.aestivum	North
223779	√	T.aestivum	North
223780	√	T.aestivum	North
223781		T.aestivum	North
223782		T.aestivum	North

223783		T.aestivum	North
223784	√	T.aestivum	North
223785		T.aestivum	North
223786		T.aestivum	North
223787		T.aestivum	North
223788		T.aestivum	North
223789		T.aestivum	North
223790	√	T.aestivum	North
223792		T.aestivum	North
223793		T.aestivum	North
223794		T.aestivum	North
223795		T.aestivum	North
223796		T.aestivum	North
223797		T.aestivum	North
223798		T.aestivum	North
223799		T.aestivum	North
223800		T.aestivum	North
223801		T.aestivum	North
223802		T.aestivum	North
223803	√	T.aestivum	North
223804		T.aestivum	North
223805	√	T.aestivum	North
223807		T.aestivum	North
223808		T.aestivum	North
223809		T.aestivum	North
223810		T.aestivum	North
223811		T.aestivum	North
223812		T.aestivum	North
223813		T.aestivum	North
223814		T.aestivum	North
223815		T.aestivum	North
223816		T.aestivum	North
223818	√	T.aestivum	North
223819	√	T.aestivum	North
223820		T.aestivum	North
223822		T.aestivum	North
223823	√	T.aestivum	North
223824		T.aestivum	North
223825		T.aestivum	North
223826		T.aestivum	North
223827	√	T.aestivum	North

223828		T.aestivum	North
223829		T.aestivum	North
223830		T.aestivum	North
223831		T.aestivum	North
223832		T.aestivum	North
223833		T.aestivum	North
223835		T.aestivum	North
223837		T.aestivum	North
223838		T.aestivum	North
223839		T.aestivum	North
223840		T.aestivum	North
223841		T.aestivum	North
223842		T.aestivum	North
223843	√	T.aestivum	North
223844		T.aestivum	North
223845		T.aestivum	North
223846	√	T.aestivum	North
223847	√	T.aestivum	North
223849	√	T.aestivum	North
223850	√	T.aestivum	North
223851		T.aestivum	North
223852		T.aestivum	North
223853		T.aestivum	North
223854		T.aestivum	North
223855		T.aestivum	North
223856	√	T.aestivum	North
223857		T.aestivum	North
223858		T.aestivum	North
223859		T.aestivum	North
223860		T.aestivum	North
223861	√	T.aestivum	North
223862	√	T.aestivum	North
223863		T.aestivum	North
223864		T.aestivum	North
223865		T.aestivum	North
223866		T.aestivum	North
223867		T.aestivum	North
223868	√	T.aestivum	North
223869		T.aestivum	North
223870	√	T.aestivum	North
223871		T.aestivum	North

223872	√	T.aestivum	North
223873		T.aestivum	North
223874		T.aestivum	North
223875		T.aestivum	North
223876		T.aestivum	North
223877		T.aestivum	North
223878		T.aestivum	North
223879		T.aestivum	North
223880	√	T.aestivum	North
223881	√	T.aestivum	North
223882		T.aestivum	North
223883		T.aestivum	North
223884		T.aestivum	North
223885		T.aestivum	North
223886		T.aestivum	North
223887	√	T.aestivum	North
223888		T.aestivum	North
223889		T.aestivum	North
223890		T.aestivum	North
223891		T.aestivum	North
225110		T.aestivum	North
225111		T.aestivum	North
225112		T.aestivum	North
225113	√	T.aestivum	North
225114		T.aestivum	North
225115		T.aestivum	North
225116	√	T.aestivum	North
225117	√	T.aestivum	North
225118		T.aestivum	North
225119		T.aestivum	North
225120		T.aestivum	North
225121		T.aestivum	North
225122		T.aestivum	North
225123		T.aestivum	North
225124		T.aestivum	North
225125		T.aestivum	North
225126		T.aestivum	North
225127		T.aestivum	North
225129	√	T.aestivum	North
225130		T.aestivum	North
225131		T.aestivum	North

225132		T.aestivum	North
225133	√	T.aestivum	North
225134		T.aestivum	North
225137		T.aestivum	North
225138		T.aestivum	North
225139		T.aestivum	North
225140	√	T.aestivum	North
225141	√	T.aestivum	North
225142		T.aestivum	North
225143		T.aestivum	North
225144		T.aestivum	North
225145		T.aestivum	North
225146		T.aestivum	North
225147		T.aestivum	North
225148	√	T.aestivum	North
225149		T.aestivum	North
225150		T.aestivum	North
225151		T.aestivum	North
225152		T.aestivum	North
225153		T.aestivum	North
225154		T.aestivum	North
225155		T.aestivum	North
225156		T.aestivum	North
225157		T.aestivum	North
225158	√	T.aestivum	North
225159		T.aestivum	North
225160		T.aestivum	North
225162		T.aestivum	North
225163		T.aestivum	North
225164		T.aestivum	North
225165		T.aestivum	North
225166	√	T.aestivum	North
225167		T.aestivum	North
225168		T.aestivum	North
225169		T.aestivum	North
225170		T.aestivum	North
225171		T.aestivum	North
225172		T.aestivum	North
225173	√	T.aestivum	North
225174		T.aestivum	North
225175		T.aestivum	North

225176		T.aestivum	North
225177		T.aestivum	North
225178		T.aestivum	North
225179		T.aestivum	North
225181		T.aestivum	North
225182		T.aestivum	North
225183		T.aestivum	North
225184		T.aestivum	North
225185		T.aestivum	North
225186	√	T.aestivum	North
225187		T.aestivum	North
225188		T.aestivum	North
225189		T.aestivum	North
225190		T.aestivum	North
225191		T.aestivum	North
225192		T.aestivum	North
225193	√	T.aestivum	North
225194		T.aestivum	North
225195		T.aestivum	North
225196		T.aestivum	North
225197	√	T.aestivum	North
225198		T.aestivum	North
225200		T.aestivum	North
225201		T.aestivum	North
225202		T.aestivum	North
225203		T.aestivum	North
225204		T.aestivum	North
225205		T.aestivum	North
225206	√	T.aestivum	North
225207		T.aestivum	North
225208		T.aestivum	North
225209		T.aestivum	North
225210	√	T.aestivum	North
225211		T.aestivum	North
225212		T.aestivum	North
225213	√	T.aestivum	North
225214		T.aestivum	North
225215	√	T.aestivum	North
225217		T.aestivum	North
225218	√	T.aestivum	North
225219		T.aestivum	North

225220		T.aestivum	North
225221	√	T.aestivum	North
225222		T.aestivum	North
225224		T.aestivum	North
225225		T.aestivum	North
225226	√	T.aestivum	North
225227		T.aestivum	North
225228		T.aestivum	North
225229		T.aestivum	North
225230	√	T.aestivum	North
225231		T.aestivum	North
225232	√	T.aestivum	North
225233		T.aestivum	North
225234		T.aestivum	North
225235		T.aestivum	North
225236		T.aestivum	North
225237		T.aestivum	North
225240		T.aestivum	North
225241		T.aestivum	North
225242		T.aestivum	North
225244		T.aestivum	North
225245		T.aestivum	North
225246		T.aestivum	North
225248		T.aestivum	North
225249		T.aestivum	North
225250		T.aestivum	North
225251	√	T.aestivum	North
225252		T.aestivum	North
225253		T.aestivum	North
225254		T.aestivum	North
225255		T.aestivum	North
225256		T.aestivum	North
225257		T.aestivum	North
225258		T.aestivum	North
225259		T.aestivum	North
225260		T.aestivum	North
225261		T.aestivum	North
225262		T.aestivum	North
225263		T.aestivum	North
225264		T.aestivum	North
225265		T.aestivum	North

225266		T.aestivum	North
225267		T.aestivum	North
225268		T.aestivum	North
225269	√	T.aestivum	North
225270		T.aestivum	North
225271	√	T.aestivum	North
225272		T.aestivum	North
225273		T.aestivum	North
225274		T.aestivum	North
225275		T.aestivum	North
225276		T.aestivum	North
225277		T.aestivum	North
225279		T.aestivum	North
225280		T.aestivum	North
225281		T.aestivum	North
225282	√	T.aestivum	North
225283		T.aestivum	North
225285		T.aestivum	North
225286		T.aestivum	North
225287		T.aestivum	North
225288		T.aestivum	North
225289		T.aestivum	North
225290		T.aestivum	North
225291		T.aestivum	North
225292		T.aestivum	North
225293		T.aestivum	North
225294		T.aestivum	North
225295	√	T.aestivum	North
225296		T.aestivum	North
225297		T.aestivum	North
225298		T.aestivum	North
225299		T.aestivum	North
225300		T.aestivum	North
225301		T.aestivum	North
225302		T.aestivum	North
225303		T.aestivum	North
225304	√	T.aestivum	North
225305		T.aestivum	North
225306		T.aestivum	North
225307	√	T.aestivum	North
225308		T.aestivum	North

225309		T.aestivum	North
225310		T.aestivum	North
225311		T.aestivum	North
225312		T.aestivum	North
225313		T.aestivum	North
225314		T.aestivum	North
225315		T.aestivum	North
225316		T.aestivum	North
225317		T.aestivum	North
225318		T.aestivum	North
225319	√	T.aestivum	North
225320		T.aestivum	North
225321	√	T.aestivum	North
225322		T.aestivum	North
225323		T.aestivum	North
225324		T.aestivum	North
225325		T.aestivum	North
225326		T.aestivum	North
225327		T.aestivum	North
225328		T.aestivum	North
225329		T.aestivum	North
225330		T.aestivum	North
225331		T.aestivum	North
225333		T.aestivum	North
225335		T.aestivum	North
225336		T.aestivum	North
225337	√	T.aestivum	North
225338		T.aestivum	North
225339		T.aestivum	North
225340		t.aestivum	North
225342		T.aestivum	North
225343		T.aestivum	North
225344		T.aestivum	North
225345		T.aestivum	North
225346		T.aestivum	North
225348		T.aestivum	North
225349		T.aestivum	North
225350		T.aestivum	North
225352		T.aestivum	North
225354		T.aestivum	North
225355	√	T.aestivum	North

225356	√	T.aestivum	North
225357		T.aestivum	North
225358		T.aestivum	North
225359	√	T.aestivum	North
225361		T.aestivum	North
225362		T.aestivum	North
225363		T.aestivum	North
225369		T.aestivum	North
225370	√	T.aestivum	North
225371		T.aestivum	North
225372		T.aestivum	North
225373		T.aestivum	North
225374	√	T.aestivum	North
225375	√	T.aestivum	North
225377		T.aestivum	North
225378		T.aestivum	North
225379		T.aestivum	North
225380		T.aestivum	North
225381	√	T.aestivum	North
225382		T.aestivum	North
225384		T.aestivum	North
225385	√	T.aestivum	North
225386		T.aestivum	North
225388		T.aestivum	North
225389		T.aestivum	North
225390	√	T.aestivum	North
225392		T.aestivum	North
225394		T.aestivum	North
225395		T.aestivum	North
225396		T.aestivum	North
225397	√	T.aestivum	North
225398		T.aestivum	North
225399		T.aestivum	North
225400		T.aestivum	North
225402		T.aestivum	North
225403		T.aestivum	North
225404		T.aestivum	North
225405		T.aestivum	North
225409		T.aestivum	North
225411		T.aestivum	North
225420		T.aestivum	North

225423	√	T.aestivum	North
225424	√	T.aestivum	North
225428		T.aestivum	North
225432		T.aestivum	North
225439		T.aestivum	North
225440		T.aestivum	North
225442		T.aestivum	North
225444	√	T.aestivum	North
225449		T.aestivum	North
225454		T.aestivum	North
225457	√	T.aestivum	North
225459		T.aestivum	North
225461		T.aestivum	North
225462		T.aestivum	North
225473		T.aestivum	North
225474		T.aestivum	North
225476		T.aestivum	North
225481	√	T.aestivum	North
225486		T.aestivum	North
225487		T.aestivum	North
225488		T.aestivum	North
225503	√	T.aestivum	North
225506		T.aestivum	North
225514		T.aestivum	North
225519		T.aestivum	North
225520		T.aestivum	North
225521		T.aestivum	North
225522		T.aestivum	North
225525		T.aestivum	North
225527		T.aestivum	North
225528	√	T.aestivum	North
225529		T.aestivum	North
225530		T.aestivum	North
225531		T.aestivum	North
225535		T.aestivum	North
225537		T.aestivum	North
225541		T.aestivum	North
225542		T.aestivum	North
225543		T.aestivum	North
225544		T.aestivum	North
225545	√	T.aestivum	North

225550	√	T.aestivum	North
225551	√	T.aestivum	North
225552		T.aestivum	North
225553		T.aestivum	North
225554		T.aestivum	North
225556		T.aestivum	North
225558		T.aestivum	North
225559		T.aestivum	North
225560		T.aestivum	North
225561		T.aestivum	North
225563		T.aestivum	North
225565		T.aestivum	North
225566		T.aestivum	North
225567		T.aestivum	North
225570		T.aestivum	North
225572		T.aestivum	North
225573	√	T.aestivum	North
225575		T.aestivum	North
225579	√	T.aestivum	North
225580		T.aestivum	North
225582		T.aestivum	North
225584		T.aestivum	North
225585	√	T.aestivum	North
225586		T.aestivum	North
225587		T.aestivum	North
225588		T.aestivum	North
225590	√	T.aestivum	North
225591		T.aestivum	North
225592		T.aestivum	North
225594		T.aestivum	North
225595		T.aestivum	North
225596		T.aestivum	North
225599		T.aestivum	North
225602		T.aestivum	North
225603		T.aestivum	North
225604		T.aestivum	North
225605		T.aestivum	North
225606		T.aestivum	North
225607		T.aestivum	North
225608		T.aestivum	North
225609	√	T.aestivum	North

225611	√	T.aestivum	North
225615		T.aestivum	North
225616		T.aestivum	North
225618		T.aestivum	North
225621		T.aestivum	North
225624		T.aestivum	North
225625		T.aestivum	North
225627		T.aestivum	North
225629	√	T.aestivum	North
225631		T.aestivum	North
225632		T.aestivum	North
225633		T.aestivum	North
225634		T.aestivum	North
225635		T.aestivum	North
225636		T.aestivum	North
225640		T.aestivum	North
225641		T.aestivum	North
225643	√	T.aestivum	North
225644		T.aestivum	North
225645		T.aestivum	North
225651		T.aestivum	North
225654		T.aestivum	North
225658		T.aestivum	North
225662		T.aestivum	North
225663	√	T.aestivum	North
225664		T.aestivum	North
225665		T.aestivum	North
225666		T.aestivum	North
225667		T.aestivum	North
225668		T.aestivum	North
225669		T.aestivum	North
225670		T.aestivum	North
225672		T.aestivum	North
225673	√	T.aestivum	North
225674		T.aestivum	North
225675	√	T.aestivum	North
225676	√	T.aestivum	North
225677		T.aestivum	North
225679		T.aestivum	North
225680		T.aestivum	North
225682		T.aestivum	North

225683		T.aestivum	North
225684		T.aestivum	North
225685		T.aestivum	North
225686		T.aestivum	North
225687		T.aestivum	North
225688		T.aestivum	North
225689	√	T.aestivum	North
225690		T.aestivum	North
225691		T.aestivum	North
225692		T.aestivum	North
225693		T.aestivum	North
225695		T.aestivum	North
225696		T.aestivum	North
225697		T.aestivum	North
225698	√	T.aestivum	North
225700	√	T.aestivum	North
225701		T.aestivum	North
225702		T.aestivum	North
225703		T.aestivum	North
225704		T.aestivum	North
225705		T.aestivum	North
225706		T.aestivum	North
225707		T.aestivum	North
225708		T.aestivum	North
225709		T.aestivum	North
225710	√	T.aestivum	North
225711		T.aestivum	North
225712	√	T.aestivum	North
225713		T.aestivum	North
225714		T.aestivum	North
225715		T.aestivum	North
225716		T.aestivum	North
225717		T.aestivum	North
225718		T.aestivum	North
225719		T.aestivum	North
225720		T.aestivum	North
225721		T.aestivum	North
225722		T.aestivum	North
225724		T.aestivum	North
225725		T.aestivum	North
225727		T.aestivum	North

225728	√	T.aestivum	North
225729		T.aestivum	North
225730		T.aestivum	North
225731		T.aestivum	North
225732		T.aestivum	North
225733		T.aestivum	North
225734		T.aestivum	North
225736	√	T.aestivum	North
225737		T.aestivum	North
225738	√	T.aestivum	North
225739		T.aestivum	North
225740		T.aestivum	North
225741		T.aestivum	North
225742		T.aestivum	North
225743		T.aestivum	North
225744	√	T.aestivum	North
225745		T.aestivum	North
225746		T.aestivum	North
225747	√	T.aestivum	North
225748		T.aestivum	North
225749		T.aestivum	North
225751		T.aestivum	North
225752		T.aestivum	North
266682		T.aestivum	North
266683	√	T.aestivum	North
266684	√	T.aestivum	North
266685		T.aestivum	North
266686	√	T.aestivum	North
266687		T.aestivum	North
266688		T.aestivum	North
266689		T.aestivum	North
266690		T.aestivum	North
266691		T.aestivum	North
266692		T.aestivum	North
266693		T.aestivum	North
266694		T.aestivum	North
266695		T.aestivum	North
266696		T.aestivum	North
266697		T.aestivum	North
266698		T.aestivum	North
266699		T.aestivum	North

266700	√	T.aestivum	North
266701	√	T.aestivum	North
266702		T.aestivum	North
266703		T.aestivum	North
266704		T.aestivum	North
266705		T.aestivum	North
266706		T.aestivum	North
266708		T.aestivum	North
266709		T.aestivum	North
266710		T.aestivum	North
266711	√	T.aestivum	North
266712		T.aestivum	North
266713		T.aestivum	North
266714		T.aestivum	North
266715		T.aestivum	North
266717		T.aestivum	North
266718		T.aestivum	North
266719		T.aestivum	North
266720	√	T.aestivum	North
266721	√	T.aestivum	North
266722		T.aestivum	North
266723		T.aestivum	North
266724		T.aestivum	North
266725		T.aestivum	North
266726		T.aestivum	North
266727	√	T.aestivum	North
266728		T.aestivum	North
266729		T.aestivum	North
266730		T.aestivum	North
266732		T.aestivum	North
266733		T.aestivum	North
266734		T.aestivum	North
266735	√	T.aestivum	North
266736		T.aestivum	North
266737	√	T.aestivum	North
266738		T.aestivum	North
266739		T.aestivum	North
266740	√	T.aestivum	North
266741		T.aestivum	North
266742		T.aestivum	North
266743		T.aestivum	North

266744		T.aestivum	North
266745		T.aestivum	North
266746		T.aestivum	North
266747		T.aestivum	North
266748		T.aestivum	North
266749		T.aestivum	North
266750		T.aestivum	North
266751		T.aestivum	North
266752		T.aestivum	North
266753		T.aestivum	North
266754	√	T.aestivum	North
266755		T.aestivum	North
266756		T.aestivum	North
266757		T.aestivum	North
266758	√	T.aestivum	North
266759		T.aestivum	North
266760		T.aestivum	North
266761		T.aestivum	North
266762		T.aestivum	North
266763	√	T.aestivum	North
266764		T.aestivum	North
266765	√	T.aestivum	North
266767		T.aestivum	North
266769	√	T.aestivum	North
320094		T.aestivum	Central
320095		T.aestivum	Central
320096		T.aestivum	Central
320097		T.aestivum	Central
320098	√	T.aestivum	Central
320099		T.aestivum	Central
320100		T.aestivum	Central
320101		T.aestivum	Central
320102		T.aestivum	Central
320103		T.aestivum	Central
320104		T.aestivum	Central
320105		T.aestivum	Central
320106		T.aestivum	Central
320107	√	T.aestivum	Central
320108		T.aestivum	Central
320109		T.aestivum	Central
320110	√	T.aestivum	Central

320111		T.aestivum	Central
320112	√	T.aestivum	Central
320113		T.aestivum	Central
320114		T.aestivum	Central
320115		T.aestivum	Central
320116		T.aestivum	Central
320117		T.aestivum	Central
320118		T.aestivum	Central
320119		T.aestivum	Central
320120		T.aestivum	Central
320121		T.aestivum	Central
320122		T.aestivum	Central
320123		T.aestivum	Central
320124		T.aestivum	Central
320125		T.aestivum	Central
320126	√	T.aestivum	Central
320127		T.aestivum	Central
320128		T.aestivum	Central
320129		T.aestivum	Central
320130		T.aestivum	Central
320131		T.aestivum	Central
320132		T.aestivum	Central
320133		T.aestivum	Central
320134		T.aestivum	Central
320135	√	T.aestivum	Central
320136		T.aestivum	Central
320137	√	T.aestivum	Central
320138		T.aestivum	Central
320139		T.aestivum	Central
320140		T.aestivum	Central
320141		T.aestivum	Central
320142		T.aestivum	Central
320143		T.aestivum	Central
320144		T.aestivum	Central
320145		T.aestivum	Central
320146	√	T.aestivum	Central
320147		T.aestivum	Central
320148		T.aestivum	Central
320149		T.aestivum	Central
320150		T.aestivum	Central
320151		T.aestivum	Central

320152		T.aestivum	Central
320153		T.aestivum	Central
320154		T.aestivum	Central
320155		T.aestivum	Central
320156		T.aestivum	Central
320157		T.aestivum	Central
320158		T.aestivum	Central
320159		T.aestivum	Central
320160		T.aestivum	Central
320161		T.aestivum	Central
320162		T.aestivum	Central
320163		T.aestivum	Central
320164		T.aestivum	Central
320165		T.aestivum	Central
320166		T.aestivum	Central
320167		T.aestivum	Central
320168		T.aestivum	Central
320169		T.aestivum	Central
320170		T.aestivum	Central
320171		T.aestivum	Central
320172		T.aestivum	Central
320173	√	T.aestivum	Central
320174	√	T.aestivum	Central
320175		T.aestivum	Central
320176	√	T.aestivum	Central
320177		T.aestivum	Central
320178		T.aestivum	Central
320179		T.aestivum	Central
320180		T.aestivum	Central
320181		T.aestivum	Central
320182		T.aestivum	Central
320183		T.aestivum	Central
320184	√	T.aestivum	Central
320185		T.aestivum	Central
320186		T.aestivum	Central
320187		T.aestivum	Central
320188		T.aestivum	Central
320189		T.aestivum	Central
320190		T.aestivum	Central
320191		T.aestivum	Central
320192		T.aestivum	Central

320193		T.aestivum	Central
320194	√	T.aestivum	Central
320195		T.aestivum	Central
320196		T.aestivum	Central
320197		T.aestivum	Central
320198	√	T.aestivum	Central
320199		T.aestivum	Central
320200		T.aestivum	Central
320201		T.aestivum	Central
320202		T.aestivum	Central
320203		T.aestivum	Central
320204	√	T.aestivum	Central
320205		T.aestivum	Central
320206		T.aestivum	Central
320207		T.aestivum	Central
320208		T.aestivum	Central
320209		T.aestivum	Central
320210		T.aestivum	Central
320211		T.aestivum	Central
320212		T.aestivum	Central
320213		T.aestivum	Central
320214	√	T.aestivum	Central
320215		T.aestivum	Central
320216		T.aestivum	Central
320217		T.aestivum	Central
320218		T.aestivum	Central
320219		T.aestivum	Central
320220	√	T.aestivum	Central
320221		T.aestivum	Central
320222		T.aestivum	Central
320223		T.aestivum	Central
320224		T.aestivum	Central
320225		T.aestivum	Central
320226		T.aestivum	Central
320227		T.aestivum	Central
320228		T.aestivum	Central
320229		T.aestivum	Central
320230		T.aestivum	Central
320231		T.aestivum	Central
320232	√	T.aestivum	Central
320233		T.aestivum	Central

320234	√	T.aestivum	Central
320235		T.aestivum	Central
320236	√	T.aestivum	Central
320237		T.aestivum	Central
320238		T.aestivum	Central
320239		T.aestivum	Central
320240		T.aestivum	Central
320241		T.aestivum	Central
320242		T.aestivum	Central
320243		T.aestivum	Central
320244		T.aestivum	Central
320245		T.aestivum	Central
320246		T.aestivum	Central
320247		T.aestivum	Central
320248		T.aestivum	Central
320249		T.aestivum	Central
320250		T.aestivum	Central
320251		T.aestivum	Central
320252		T.aestivum	Central
320253		T.aestivum	Central
320254		T.aestivum	Central
320255		T.aestivum	Central
320256		T.aestivum	Central
320257		T.aestivum	Central
320258		T.aestivum	Central
320259		T.aestivum	Central
320260		T.aestivum	Central
320261		T.aestivum	Central
320262		T.aestivum	Central
320263		T.aestivum	Central
320264		T.aestivum	Central
320265		T.aestivum	Central
320266		T.aestivum	Central
320267		T.aestivum	Central
320268		T.aestivum	Central
320269		T.aestivum	Central
320270		T.aestivum	Central
320271		T.aestivum	Central
320272		T.aestivum	Central
320273		T.aestivum	Central
320274		T.aestivum	Central

320275		T.aestivum	Central
320276		T.aestivum	Central
320277		T.aestivum	Central
320278		T.aestivum	Central
320279		T.aestivum	Central
320280		T.aestivum	Central
320281		T.aestivum	Central
320282		T.aestivum	Central
320283		T.aestivum	Central
320284		T.aestivum	Central
320285		T.aestivum	Central
320286	√	T.aestivum	Central
320287		T.aestivum	Central
320288		T.aestivum	Central
320289		T.aestivum	Central
320290		T.aestivum	Central
320291		T.aestivum	Central
320292		T.aestivum	Central
320293		T.aestivum	Central
320294		T.aestivum	Central
320295		T.aestivum	Central
320296		T.aestivum	Central
320297		T.aestivum	Central
320298		T.aestivum	Central
320299		T.aestivum	Central
320300		T.aestivum	Central
320301		T.aestivum	Central
320302	√	T.aestivum	Central
320303		T.aestivum	Central
320304		T.aestivum	Central
320305		T.aestivum	Central
320306		T.aestivum	Central
320307		T.aestivum	Central
320308	√	T.aestivum	Central
320309	√	T.aestivum	Central
320310		T.aestivum	Central
320312		T.aestivum	Central
320313		T.aestivum	Central
320314		T.aestivum	Central
320315		T.aestivum	Central
320316		T.aestivum	Central

320317		T.aestivum	Central
320318		T.aestivum	Central
320319	√	T.aestivum	Central
320320		T.aestivum	Central
320321		T.aestivum	Central
320322		T.aestivum	Central
320323		T.aestivum	Central
320324		T.aestivum	Central
320325		T.aestivum	Central
320326		T.aestivum	Central
320327		T.aestivum	Central
320328		T.aestivum	Central
320329		T.aestivum	Central
320330		T.aestivum	Central
320331	√	T.aestivum	Central
320332		T.aestivum	Central
320333	√	T.aestivum	Central
320334		T.aestivum	Central
320335		T.aestivum	Central
320336		T.aestivum	Central
320337		T.aestivum	Central
320338		T.aestivum	Central
320339		T.aestivum	Central
320340		T.aestivum	Central
320341		T.aestivum	Central
320342		T.aestivum	Central
320343		T.aestivum	Central
320344		T.aestivum	Central
320345		T.aestivum	Central
320346		T.aestivum	Central
320347		T.aestivum	Central
320348		T.aestivum	Central
320349		T.aestivum	Central
320350	√	T.aestivum	Central
320351		T.aestivum	Central
320352		T.aestivum	Central
320353		T.aestivum	Central
320354	√	T.aestivum	Central
320355		T.aestivum	Central
320356		T.aestivum	Central
320357		T.aestivum	Central

320358	√	T.aestivum	Central
320359		T.aestivum	Central
320360	√	T.aestivum	Central
320361		T.aestivum	Central
320362		T.aestivum	Central
320363		T.aestivum	Central
320364		T.aestivum	Central
320365		T.aestivum	Central
320366		T.aestivum	Central
320367		T.aestivum	Central
320368	√	T.aestivum	Central
320369		T.aestivum	Central
320370		T.aestivum	Central
320371		T.aestivum	Central
320372		T.aestivum	Central
320373		T.aestivum	Central
320374		T.aestivum	Central
320375		T.aestivum	Central
320376	√	T.aestivum	Central
320377		T.aestivum	Central
320378		T.aestivum	Central
320379		T.aestivum	Central
320380		T.aestivum	Central
320381		T.aestivum	Central
320382		T.aestivum	Central
320383		T.aestivum	Central
320384		T.aestivum	Central
320385		T.aestivum	Central
320386		T.aestivum	Central
320387		T.aestivum	Central
320388		T.aestivum	Central
320389	√	T.aestivum	Central
320390	√	T.aestivum	Central
320391		T.aestivum	Central
320392		T.aestivum	Central
320393		T.aestivum	Central
320394		T.aestivum	Central
320395		T.aestivum	Central
320396	√	T.aestivum	Central
320397		T.aestivum	Central
320398		T.aestivum	Central

320399		T.aestivum	Central
320400		T.aestivum	Central
320401		T.aestivum	Central
320402		T.aestivum	Central
320403		T.aestivum	Central
320404		T.aestivum	Central
320405		T.aestivum	Central
320406		T.aestivum	Central
320407		T.aestivum	Central
320408		T.aestivum	Central
320409	√	T.aestivum	Central
320410		T.aestivum	Central
320411		T.aestivum	Central
320412	√	T.aestivum	Central
320413		T.aestivum	Central
320414		T.aestivum	Central
320415		T.aestivum	Central
320416		T.aestivum	Central
320417		T.aestivum	Central
320418		T.aestivum	Central
320419		T.aestivum	Central
320420		T.aestivum	Central
320421		T.aestivum	Central
320422		T.aestivum	Central
320423		T.aestivum	Central
320424		T.aestivum	Central
320425		T.aestivum	Central
320426		T.aestivum	Central
320427		T.aestivum	Central
320428		T.aestivum	Central
320429		T.aestivum	Central
320430		T.aestivum	Central
320431		T.aestivum	Central
320432		T.aestivum	Central
320433		T.aestivum	Central
320434	√	T.aestivum	Central
320435		T.aestivum	Central
320436		T.aestivum	Central
320437		T.aestivum	Central
320438	√	T.aestivum	Central
320439		T.aestivum	Central

320440		T.aestivum	Central
320441		T.aestivum	Central
320442		T.aestivum	Central
320443		T.aestivum	Central
320444		T.aestivum	Central
320445		T.aestivum	Central
320446		T.aestivum	Central
320447		T.aestivum	Central
320448		T.aestivum	Central
320449		T.aestivum	Central
320450		T.aestivum	Central
320451		T.aestivum	Central
320452		T.aestivum	Central
320453	√	T.aestivum	Central
320454		T.aestivum	Central
320455		T.aestivum	Central
320456		T.aestivum	Central
320457		T.aestivum	Central
320458		T.aestivum	Central
320459		T.aestivum	Central
320460		T.aestivum	Central
320461		T.aestivum	Central
320462		T.aestivum	Central
320463		T.aestivum	Central
320464		T.aestivum	Central
320465	√	T.aestivum	Central
320466		T.aestivum	Central
320467		T.aestivum	Central
320468		T.aestivum	Central
320469		T.aestivum	Central
320470	√	T.aestivum	Central
320471		T.aestivum	Central
320472		T.aestivum	Central
320473		T.aestivum	Central
320475	√	T.aestivum	Central
320476		T.aestivum	Central
320477		T.aestivum	Central
320478		T.aestivum	Central
320479	√	T.aestivum	Central
320480	√	T.aestivum	Central
320481		T.aestivum	Central

320482	√	T.aestivum	Central
320483		T.aestivum	Central
320484		T.aestivum	Central
320485		T.aestivum	Central
320486	√	T.aestivum	Central
320487	√	T.aestivum	Central
320488		T.aestivum	Central
320489		T.aestivum	Central
320490		T.aestivum	Central
320491		T.aestivum	Central
320492		T.aestivum	Central
320493		T.aestivum	Central
320494		T.aestivum	Central
320495		T.aestivum	Central
320496		T.aestivum	Central
320497		T.aestivum	Central
320498		T.aestivum	Central
320499		T.aestivum	Central
320500		T.aestivum	Central
320501		T.aestivum	Central
320502		T.aestivum	Central
320503		T.aestivum	Central
320504		T.aestivum	Central
320506		T.aestivum	Central
320507		T.aestivum	Central
320508		T.aestivum	Central
320510		T.aestivum	Central
320511		T.aestivum	Central
320512		T.aestivum	Central
320513	√	T.aestivum	Central
320514		T.aestivum	Central
320515		T.aestivum	Central
320516		T.aestivum	Central
320517		T.aestivum	Central
320518		T.aestivum	Central
320519		T.aestivum	Central
320520		T.aestivum	Central
320521		T.aestivum	Central
320522		T.aestivum	Central
320523		T.aestivum	Central
320524		T.aestivum	Central

320525		T.aestivum	Central
320526		T.aestivum	Central
320527		T.aestivum	Central
320528		T.aestivum	Central
320529		T.aestivum	Central
320530	√	T.aestivum	Central
320531		T.aestivum	Central
320532		T.aestivum	Central
320533		T.aestivum	Central
320534		T.aestivum	Central
320535		T.aestivum	Central
320536	√	T.aestivum	Central
320537	√	T.aestivum	Central
320538		T.aestivum	Central
320539		T.aestivum	Central
320540		T.aestivum	Central
320541		T.aestivum	Central
320542		T.aestivum	Central
320543		T.aestivum	Central
320544		T.aestivum	Central
320546		T.aestivum	Central
320547		T.aestivum	Central
320548		T.aestivum	Central
320549		T.aestivum	Central
320551		T.aestivum	Central
320552		T.aestivum	Central
320553		T.aestivum	Central
320554		T.aestivum	Central
320555	√	T.aestivum	Central
320556		T.aestivum	Central
320557		T.aestivum	Central
320558		T.aestivum	Central
320559		T.aestivum	Central
320560		T.aestivum	Central
320561		T.aestivum	Central
320562		T.aestivum	Central
320563		T.aestivum	Central
320565		T.aestivum	Central
320566	√	T.aestivum	Central
320567		T.aestivum	Central
320568		T.aestivum	Central

320569		T.aestivum	Central
320570	√	T.aestivum	Central
320571		T.aestivum	Central
320572	√	T.aestivum	Central
320573		T.aestivum	Central
320575		T.aestivum	Central
320576		T.aestivum	Central
320577		T.aestivum	Central
320578		T.aestivum	Central
320580		T.aestivum	Central
320581		T.aestivum	Central
320582		T.aestivum	Central
320583	√	T.aestivum	Central
320584		T.aestivum	Central
320585		T.aestivum	Central
320586		T.aestivum	Central
320587		T.aestivum	Central
320588		T.aestivum	Central
320589		T.aestivum	Central
320590		T.aestivum	Central
320591		T.aestivum	Central
320592	√	T.aestivum	Central
320593		T.aestivum	Central
320594		T.aestivum	Central
320595		T.aestivum	Central
320596		T.aestivum	Central
320597		T.aestivum	Central
2248423		T.aestivum	North
2248424		T.aestivum	North
2248425		T.aestivum	North
2248426		T.aestivum	North
2248427		T.aestivum	North
2248428		T.aestivum	North
2248429		T.aestivum	North
2248430		T.aestivum	North
2248431		T.aestivum	North
2248432		T.aestivum	North
2248433		T.aestivum	North
2248434		T.aestivum	North
2248435	√	T.aestivum	North
2248436		T.aestivum	North

2248437		T.aestivum	North
2248438		T.aestivum	North
2248439		T.aestivum	North
2248440		T.aestivum	North
2248441	√	T.aestivum	North
2248446	√	T.aestivum	North
2248447		T.aestivum	North
2248448	√	T.aestivum	North
2248449		T.aestivum	North
2248450		T.aestivum	North
2248451	√	T.aestivum	North
2248452		T.aestivum	North
2248453	√	T.aestivum	North
2248454		T.aestivum	North
2248455		T.aestivum	North
2248456		T.aestivum	North
2248457		T.aestivum	North
2248458		T.aestivum	North
2248459		T.aestivum	North
2248460		T.aestivum	North
2248461		T.aestivum	North
2248462		T.aestivum	North
2248465		T.aestivum	North
2248466		T.aestivum	North
2248467		T.aestivum	North
2248468	√	T.aestivum	North
2248469		T.aestivum	North
2248470	√	T.aestivum	North
2248471		T.aestivum	North
2248472		T.aestivum	North
2248473	√	T.aestivum	North
2248474		T.aestivum	North
2248475		T.aestivum	North
2248476	√	T.aestivum	North
2248477	√	T.aestivum	North
2248478		T.aestivum	North
2248479		T.aestivum	North
2248480		T.aestivum	North
2248481		T.aestivum	North
2248482		T.aestivum	North
2248483		T.aestivum	North

2248484		T.aestivum	North
2248485		T.aestivum	North
2248486		T.aestivum	North
2248487		T.aestivum	North
2248488		T.aestivum	North
2248489		T.aestivum	North
2248490		T.aestivum	North
2248491		T.aestivum	North
2248492		T.aestivum	North
2248493		T.aestivum	North
2248494	√	T.aestivum	North
2248495		T.aestivum	North
2248496		T.aestivum	North
2248497		T.aestivum	North
2248498	√	T.aestivum	North
2248500		T.aestivum	North
2248501		T.aestivum	North
2248502	√	T.aestivum	North
2248503		T.aestivum	North
2248504		T.aestivum	North
2248505		T.aestivum	North
2248506	√	T.aestivum	North
2248508		T.aestivum	North
2248509		T.aestivum	North
2248510		T.aestivum	North
2248511		T.aestivum	North
2248512		T.aestivum	North
2248513	√	T.aestivum	North
2248514		T.aestivum	North
2248515	√	T.aestivum	North
2248516		T.aestivum	North
2248517		T.aestivum	North
2248518		T.aestivum	North
2248519		T.aestivum	North
2248520		T.aestivum	North
2248521		T.aestivum	North
2248522		T.aestivum	North
2248523		T.aestivum	North
2248524		T.aestivum	North
2248525		T.aestivum	North
2248526	√	T.aestivum	North

2248527	√	T.aestivum	North
2248528		T.aestivum	North
2248529		T.aestivum	North
2248530	√	T.aestivum	North
2248531		T.aestivum	North
2248532		T.aestivum	North
2248533		T.aestivum	North
2248534		T.aestivum	North
2248535		T.aestivum	North
2248536		T.aestivum	North
2248537		T.aestivum	North
2248538	√	T.aestivum	North
2248539		T.aestivum	North
2248540		T.aestivum	North
2248541		T.aestivum	North
2248542		T.aestivum	North
2248543		T.aestivum	North
2248544		T.aestivum	North
2248545		T.aestivum	North
2248546		T.aestivum	North
2248547		T.aestivum	North
2248548		T.aestivum	North
2248549		T.aestivum	North
2248550		T.aestivum	North
2248551		T.aestivum	North
2248552		T.aestivum	North
2248553	√	T.aestivum	North
2248554		T.aestivum	North
2248555		T.aestivum	North
2248556		T.aestivum	North
2248557		T.aestivum	North
2248558		T.aestivum	North
2248559		T.aestivum	North
2248560		T.aestivum	North
2248561		T.aestivum	North
2248563		T.aestivum	North
2248564		T.aestivum	North
2248565		T.aestivum	North
2248566		T.aestivum	North
2248567	√	T.aestivum	North
2248568		T.aestivum	North

2248569		T.aestivum	North
2248570		T.aestivum	North
2248571		T.aestivum	North
2248572		T.aestivum	North
2248573		T.aestivum	North
2248574		T.aestivum	North
2248575		T.aestivum	North
2248576		T.aestivum	North
2248577		T.aestivum	North
2248578		T.aestivum	North
2248579		T.aestivum	North
2248580		T.aestivum	North
2248581	√	T.aestivum	North
2248582		T.aestivum	North
2248583		T.aestivum	North
2248584		T.aestivum	North
2248585		T.aestivum	North
2248586		T.aestivum	North
2248587		T.aestivum	North
2248588		T.aestivum	North
2248589		T.aestivum	North
2248590		T.aestivum	North
2248591		T.aestivum	North
2248592		T.aestivum	North
2248593		T.aestivum	North
2248594		T.aestivum	North
2248596	√	T.aestivum	North
2248597		T.aestivum	North
2248598		T.aestivum	North
2248599		T.aestivum	North
2248600	√	T.aestivum	North
2248601	√	T.aestivum	North
2248602		T.aestivum	North
2248603		T.aestivum	North
2248605		T.aestivum	North
2248606		T.aestivum	North
2248607		T.aestivum	North
2248608		T.aestivum	North
2248609	√	T.aestivum	North
2248610		T.aestivum	North
2248611		T.aestivum	North

2248612		T.aestivum	North
2248613		T.aestivum	North
2248614	√	T.aestivum	North
2248615		T.aestivum	North
2248616		T.aestivum	North
2248617	√	T.aestivum	North
2248618		T.aestivum	North
2248619		T.aestivum	North
2248620		T.aestivum	North
2248621		T.aestivum	North
2248622	√	T.aestivum	North
2248623		T.aestivum	North
2248624	√	T.aestivum	North
2248625		T.aestivum	North
2248627	√	T.aestivum	North
2248628		T.aestivum	North
2248629		T.aestivum	North
2248630		T.aestivum	North
2248631		T.aestivum	North
2248632		T.aestivum	North
2248633		T.aestivum	North
2248634		T.aestivum	North
2248635		T.aestivum	North
2248636		T.aestivum	North
2248637		T.aestivum	North
2248638		T.aestivum	North
2248639		T.aestivum	North
2248640		T.aestivum	North
2248641		T.aestivum	North
2248642		T.aestivum	North
2248643		T.aestivum	North
2248644		T.aestivum	North
2248645		T.aestivum	North
2248646		T.aestivum	North
2248647		T.aestivum	North
2248648		T.aestivum	North
2248649		T.aestivum	North
2248650		T.aestivum	North
2248651		T.aestivum	North
2248652		T.aestivum	North
2248653		T.aestivum	North

2248654		T.aestivum	North
2248655		T.aestivum	North
2248656		T.aestivum	North
2248657		T.aestivum	North
2248658		T.aestivum	North
2248659		T.aestivum	North
2248660		T.aestivum	North
2248661		T.aestivum	North
2248662	√	T.aestivum	North
2248663		T.aestivum	North
2248664		T.aestivum	North
2248665		T.aestivum	North
2248666		T.aestivum	North
2248667		T.aestivum	North
2248668	√	T.aestivum	North
2248669		T.aestivum	North
2248670		T.aestivum	North
2248671		T.aestivum	North
2248672	√	T.aestivum	North
2248673		T.aestivum	North
2248674		T.aestivum	North
2248675	√	T.aestivum	North
2248676		T.aestivum	North
2248677		T.aestivum	North
2248678		T.aestivum	North
2248679		T.aestivum	North
2248680		T.aestivum	North
2248681		T.aestivum	North
2248682		T.aestivum	North
2248683		T.aestivum	North
2248684		T.aestivum	North
2248685		T.aestivum	North
2248686		T.aestivum	North
2248687		T.aestivum	North
2248688		T.aestivum	North
2248689		T.aestivum	North
2248690		T.aestivum	North
2248691		T.aestivum	North
2248692		T.aestivum	North
2248693	√	T.aestivum	North
2248694		T.aestivum	North

2248695		T.aestivum	North
2248696		T.aestivum	North
2248697		T.aestivum	North
2248698		T.aestivum	North
2248699		T.aestivum	North
2248700		T.aestivum	North
2248701		T.aestivum	North
2248702		T.aestivum	North
2248703		T.aestivum	North
2248704		T.aestivum	North
2249305	√	T.aestivum	North
2249306	√	T.aestivum	North
2249307		T.aestivum	North
2249308	√	T.aestivum	North
2249309	√	T.aestivum	North
2249310		T.aestivum	North
2249311		T.aestivum	North
2249312		T.aestivum	North
2249313		T.aestivum	North
2249314		T.aestivum	North
2249315		T.aestivum	North
2249316		T.aestivum	North
2249317		T.aestivum	North
2249318		T.aestivum	North
2249319		T.aestivum	North
2249320		T.aestivum	North
2249324		T.aestivum	North
2249325		T.aestivum	North
2249326	√	T.aestivum	North
2249327	√	T.aestivum	North
2249328		T.aestivum	North
2249329		T.aestivum	North
2249330		T.aestivum	North
2249331	√	T.aestivum	North
2249332		T.aestivum	North
2249333	√	T.aestivum	North
2249334	√	T.aestivum	North
2249335		T.aestivum	North
2249336		T.aestivum	North
2249337		T.aestivum	North
2249338		T.aestivum	North

2249339		T.aestivum	North
2249340		T.aestivum	North
2249341		T.aestivum	North
2249342		T.aestivum	North
2249343		T.aestivum	North
2249344	√	T.aestivum	North
2249345	√	T.aestivum	North
2249348	√	T.aestivum	North
2249350		T.aestivum	North
2249351		T.aestivum	North
2249352		T.aestivum	North
2249353		T.aestivum	North
2249354		T.aestivum	North
2249355		T.aestivum	North
2249356		T.aestivum	North
2249357		T.aestivum	North
2249358	√	T.aestivum	North
2249361		T.aestivum	North
2249362		T.aestivum	North
2249363	√	T.aestivum	North
2249364		T.aestivum	North
2249365	√	T.aestivum	North
2249366		T.aestivum	North
2249367		T.aestivum	North
2249368		T.aestivum	North
2249369		T.aestivum	North
2249370		T.aestivum	North
2249371		T.aestivum	North
2249372	√	T.aestivum	North
2249373		T.aestivum	North
2249374		T.aestivum	North
2249375		T.aestivum	North
2249377		T.aestivum	North
2249378		T.aestivum	North
2249379		T.aestivum	North
2249380		T.aestivum	North
2249381		T.aestivum	North
2249382		T.aestivum	North
2249383		T.aestivum	North
2249384		T.aestivum	North
2249385		T.aestivum	North

2249386	√	T.aestivum	North
2249387		T.aestivum	North
2249388		T.aestivum	North
2249389		T.aestivum	North
2249390		T.aestivum	North
2249391		T.aestivum	North
2249392		T.aestivum	North
2249393		T.aestivum	North
2249394		T.aestivum	North
2249395		T.aestivum	North
2249397		T.aestivum	North
2249398		T.aestivum	North
2249399	√	T.aestivum	North
2249400		T.aestivum	North
2249401		T.aestivum	North
2249402		T.aestivum	North
2249403		T.aestivum	North
2249404		T.aestivum	North
2249405		T.aestivum	North
2249406	√	T.aestivum	North
2249407		T.aestivum	North
2249408		T.aestivum	North
2249409		T.aestivum	North
2249410		T.aestivum	North
2249411	√	T.aestivum	North
2249412		T.aestivum	North
2249413	√	T.aestivum	North
2249414		T.aestivum	North
2249415		T.aestivum	North
2249416		T.aestivum	North
2249417	√	T.aestivum	North
2249418		T.aestivum	North
2249419		T.aestivum	North
2249420		T.aestivum	North
2249421		T.aestivum	North
2249422		T.aestivum	North
2249423		T.aestivum	North
2249424		T.aestivum	North
2249425		T.aestivum	North
2249426		T.aestivum	North
2249428		T.aestivum	North

2249429		T.aestivum	North
2249430		T.aestivum	North
2249431		T.aestivum	North
2249432	√	T.aestivum	North
2249433		T.aestivum	North
2249434	√	T.aestivum	North
2249435		T.aestivum	North
2249436		T.aestivum	North
2249437	√	T.aestivum	North
2249438		T.aestivum	North
2249439		T.aestivum	North
2249440		T.aestivum	North
2249441	√	T.aestivum	North
2249442		T.aestivum	North
2249443	√	T.aestivum	North
2249444		T.aestivum	North
2249445		T.aestivum	North
2249446		T.aestivum	North
2249447		T.aestivum	North
2249448		T.aestivum	North
2249451		T.aestivum	North
2249452		T.aestivum	North
2249454		T.aestivum	North
2249455		T.aestivum	North
2249456		T.aestivum	North
2249457		T.aestivum	North
2249458		T.aestivum	North
2249459		T.aestivum	North
2249460		T.aestivum	North
2249461		T.aestivum	North
2249462	√	T.aestivum	North
2249463		T.aestivum	North
2249464		T.aestivum	North
2249465		T.aestivum	North
2249466		T.aestivum	North
2249467		T.aestivum	North
2249468		T.aestivum	North
2249469		T.aestivum	North
2249470		T.aestivum	North
2249471		T.aestivum	North
2249472		T.aestivum	North

2249473		T.aestivum	North
2249474		T.aestivum	North
2249475		T.aestivum	North
2249476		T.aestivum	North
2249477	√	T.aestivum	North
2249478		T.aestivum	North
2249479		T.aestivum	North
2249481	√	T.aestivum	North
2249482		T.aestivum	North
2249483		T.aestivum	North
2249484		T.aestivum	North
2249485		T.aestivum	North
2249486		T.aestivum	North
2249487		T.aestivum	North
2249488		T.aestivum	North
2249489		T.aestivum	North
2249490	√	T.aestivum	North
2249491		T.aestivum	North
2249492		T.aestivum	North
2249494		T.aestivum	North
2249495		T.aestivum	North
2249496		T.aestivum	North
2249498	√	T.aestivum	North
2249499		T.aestivum	North
2249500	√	T.aestivum	North
2249501		T.aestivum	North
2249502		T.aestivum	North
2249503		T.aestivum	North
2249504	√	T.aestivum	North
2249505		T.aestivum	North
2249506		T.aestivum	North
2249507		T.aestivum	North
2249508		T.aestivum	North
2249509		T.aestivum	North
2249510		T.aestivum	North
2249511		T.aestivum	North
2249512	√	T.aestivum	North
2249513		T.aestivum	North
2249514		T.aestivum	North
2249515		T.aestivum	North
2249516		T.aestivum	North

2249517		T.aestivum	North
2249518		T.aestivum	North
2249519		T.aestivum	North
2249520		T.aestivum	North
2249521		T.aestivum	North
2249522		T.aestivum	North
2249523		T.aestivum	North
2249524		T.aestivum	North
2249525		T.aestivum	North
2249526		T.aestivum	North
2249527		T.aestivum	North
2249528	√	T.aestivum	North
2249529		T.aestivum	North
2249530	√	T.aestivum	North
2249531		T.aestivum	North
2249532		T.aestivum	North
2249533	√	T.aestivum	North
2249534		T.aestivum	North
2249535		T.aestivum	North
2249536		T.aestivum	North
2249537		T.aestivum	North
2249538		T.aestivum	North
2249539	√	T.aestivum	North
2249540		T.aestivum	North
2249541		T.aestivum	North
2249542		T.aestivum	North
2249544		T.aestivum	North
2249545		T.aestivum	North
2249546		T.aestivum	North
2249547	√	T.aestivum	North
2249548		T.aestivum	North
2249549		T.aestivum	North
2249550		T.aestivum	North
2249551		T.aestivum	North
2249552		T.aestivum	North
2249553	√	T.aestivum	North
2249554		T.aestivum	North
2249555		T.aestivum	North
2249556		T.aestivum	North
2249558		T.aestivum	North
2249559		T.aestivum	North

2249560		T.aestivum	North
2249561		T.aestivum	North
2249562		T.aestivum	North
2249563		T.aestivum	North
2249565		T.aestivum	North
2249567		T.aestivum	North
2249568		T.aestivum	North
2249569		T.aestivum	North
2249571		T.aestivum	North
2249572		T.aestivum	North
2249573		T.aestivum	North
2249574	√	T.aestivum	North
2249576		T.aestivum	North
2249577		T.aestivum	North
2249578		T.aestivum	North
2249579		T.aestivum	North
2249580		T.aestivum	North
2249581		T.aestivum	North
2249582		T.aestivum	North
2249583		T.aestivum	North
2249584		T.aestivum	North
2249585		T.aestivum	North
2249586	√	T.aestivum	North
2249587		T.aestivum	North
2249588	√	T.aestivum	North
2249590		T.aestivum	North
2249591		T.aestivum	North
2249592	√	T.aestivum	North
2249593		T.aestivum	North
2249594	√	T.aestivum	North
2249595		T.aestivum	North
2249596		T.aestivum	North
2249597		T.aestivum	North
2249598		T.aestivum	North
2249599		T.aestivum	North
2249600		T.aestivum	North
2249601		T.aestivum	North
2249602		T.aestivum	North
2249603		T.aestivum	North
2249604		T.aestivum	North
2249605		T.aestivum	South

2249606		T.aestivum	South
2249607		T.aestivum	South
2249608		T.aestivum	South
2249609		T.aestivum	South
2249610		T.aestivum	South
2249611		T.aestivum	South
2249612		T.aestivum	South
2249613		T.aestivum	South
2249614	√	T.aestivum	South
2249615		T.aestivum	South
2249616		T.aestivum	South
2249617		T.aestivum	South
2249619	√	T.aestivum	South
2249620		T.aestivum	South
2249621		T.aestivum	South
2249622		T.aestivum	South
2249623		T.aestivum	South
2249624		T.aestivum	South
2249625		T.aestivum	South
2249626		T.aestivum	South
2249627		T.aestivum	South
2249628		T.aestivum	South
2249629	√	T.aestivum	South
2249630		T.aestivum	South
2249631		T.aestivum	South
2249632		T.aestivum	South
2249633		T.aestivum	South
2249634	√	T.aestivum	South
2249635		T.aestivum	South
2249636		T.aestivum	South
2249637		T.aestivum	South
2249638		T.aestivum	South
2249639		T.aestivum	South
2249640		T.aestivum	South
2249641		T.aestivum	South
2249642		T.aestivum	South
2249643		T.aestivum	South
2249644		T.aestivum	South
2249645		T.aestivum	South
2249646		T.aestivum	South
2249647		T.aestivum	South

2249648		T.aestivum	South
2249649		T.aestivum	South
2249650		T.aestivum	South
2249651		T.aestivum	South
2249652	√	T.aestivum	South
2249653	√	T.aestivum	South
2249654		T.aestivum	South
2249655		T.aestivum	South
2249656		T.aestivum	South
2249657		T.aestivum	South
2249658		T.aestivum	South
2249659		T.aestivum	South
2249660		T.aestivum	South
2249661		T.aestivum	South
2249662		T.aestivum	South
2249663		T.aestivum	South
2249664		T.aestivum	South
2249665		T.aestivum	South
2249666		T.aestivum	South
2249667		T.aestivum	South
2249668		T.aestivum	South
2249669		T.aestivum	South
2249670		T.aestivum	South
2249671		T.aestivum	South
2249672		T.aestivum	South
2249673		T.aestivum	South
2249674		T.aestivum	South
2249675		T.aestivum	South
2249676		T.aestivum	South
2249677		T.aestivum	South
2249678		T.aestivum	South
2249679	√	T.aestivum	South
2249680		T.aestivum	South
2249681		T.aestivum	South
2249682		T.aestivum	South
2249683		T.aestivum	South
2249684		T.aestivum	South
2249685		T.aestivum	South
2249686		T.aestivum	South
2249687		T.aestivum	South
2249688		T.aestivum	South

2249689	√	T.aestivum	South
2249690		T.aestivum	South
2249691		T.aestivum	South
2249692		T.aestivum	South
2249693		T.aestivum	South
2249694		T.aestivum	South
2249695		T.aestivum	South
2249696		T.aestivum	South
2249697		T.aestivum	South
2249698		T.aestivum	South
2249699		T.aestivum	South
2249700		T.aestivum	South
2249701		T.aestivum	South
2249702		T.aestivum	South
2249703	√	T.aestivum	South
2249704		T.aestivum	South
2249705		T.aestivum	South
2249706		T.aestivum	South
2249707		T.aestivum	South
2249708		T.aestivum	South
2249709		T.aestivum	South
2249710		T.aestivum	South
2249711	√	T.aestivum	South
2249712	√	T.aestivum	South
2249713		T.aestivum	South
2249714		T.aestivum	South
2249716		T.aestivum	South
2249717		T.aestivum	South
2249718		T.aestivum	South
2249719		T.aestivum	South
2249720		T.aestivum	South
2249721		T.aestivum	South
2249723		T.aestivum	South
2249724		T.aestivum	South
2249725		T.aestivum	South
2249726		T.aestivum	South
2249727		T.aestivum	South
2249728		T.aestivum	South
2249729		T.aestivum	South
2249730		T.aestivum	South
2249731		T.aestivum	South

2249732		T.aestivum	South
2249733		T.aestivum	South
2249734		T.aestivum	South
2249736		T.aestivum	South
2249737		T.aestivum	South
2249738	√	T.aestivum	South
2249739	√	T.aestivum	South
2249741		T.aestivum	South
2249742		T.aestivum	South
2249743		T.aestivum	South
2249744		T.aestivum	South
2249745		T.aestivum	South
2249746		T.aestivum	South
2249747		T.aestivum	South
2249748		T.aestivum	South
2249749	√	T.aestivum	South
2249750		T.aestivum	South
2249751		T.aestivum	South
2249752		T.aestivum	South
2249753		T.aestivum	South
2249754		T.aestivum	South
2249755		T.aestivum	South
2249756	√	T.aestivum	South
2249757		T.aestivum	South
2249758		T.aestivum	South
2249759		T.aestivum	South
2249761	√	T.aestivum	South
2249762		T.aestivum	South
2249763		T.aestivum	South
2249764		T.aestivum	South
2249765		T.aestivum	South
2249766		T.aestivum	South
2249767	√	T.aestivum	South
2249768	√	T.aestivum	South
2249769		T.aestivum	South
2249771		T.aestivum	South
2249772	√	T.aestivum	South
2249773		T.aestivum	South
2249774		T.aestivum	South
2249775		T.aestivum	South
2249778		T.aestivum	South

2249779		T.aestivum	South
2249780		T.aestivum	South
2249781		T.aestivum	South
2249782		T.aestivum	South
2249783		T.aestivum	South
2249785		T.aestivum	South
2249786		T.aestivum	South
2249787		T.aestivum	South
2249788		T.aestivum	South
2249790		T.aestivum	South
2249791		T.aestivum	South
2249792		T.aestivum	South
2249793		T.aestivum	South
2249794		T.aestivum	South
2249795		T.aestivum	South
2249796		T.aestivum	South
2249797	√	T.aestivum	South
2249798		T.aestivum	South
2249799		T.aestivum	South
2249800	√	T.aestivum	South
2249801		T.aestivum	South
2249802		T.aestivum	South
2249804		T.aestivum	South
2249805		T.aestivum	South
2249806		T.aestivum	South
2249807		T.aestivum	South
2249808		T.aestivum	South
2249809		T.aestivum	South
2249810		T.aestivum	South
2249811		T.aestivum	South
2249812		T.aestivum	South
2249813		T.aestivum	South
2249814		T.aestivum	South
2249815		T.aestivum	South
2249816		T.aestivum	South
2249817		T.aestivum	South
2249818		T.aestivum	South
2249819	√	T.aestivum	South
2249820		T.aestivum	South
2249821		T.aestivum	South
2249822		T.aestivum	South

2249823		T.aestivum	South
2249824		T.aestivum	South
2249825		T.aestivum	South
2249826		T.aestivum	South
2249827		T.aestivum	South
2249828		T.aestivum	South
2249829		T.aestivum	South
2249830		T.aestivum	South
2249831		T.aestivum	South
2249832		T.aestivum	South
2249833		T.aestivum	South
2249834		T.aestivum	South
2249835		T.aestivum	South
2249836		T.aestivum	South
2249837		T.aestivum	South
2249838		T.aestivum	South
2249839		T.aestivum	South
2249840	√	T.aestivum	South
2249841		T.aestivum	South
2249842		T.aestivum	South
2249843		T.aestivum	South
2249844		T.aestivum	South
2249845		T.aestivum	South
2249846		T.aestivum	South
2249847		T.aestivum	South
2249848		T.aestivum	South
2249849		T.aestivum	South
2249851	√	T.aestivum	South
2249852		T.aestivum	South
2249853		T.aestivum	South
2249854		T.aestivum	South
2249855		T.aestivum	South
2249856		T.aestivum	South
2249857	√	T.aestivum	South
2249858		T.aestivum	South
2249859		T.aestivum	South
2249860		T.aestivum	South
2249861	√	T.aestivum	South
2249862		T.aestivum	South
2249863	√	T.aestivum	South
2249864		T.aestivum	South

2249865		T.aestivum	South
2249866		T.aestivum	South
2249867		T.aestivum	South
2249868		T.aestivum	South
2249869		T.aestivum	South
2249870		T.aestivum	South
2249871		T.aestivum	South
2249872		T.aestivum	South
2249873		T.aestivum	South
2249875		T.aestivum	South
2249876		T.aestivum	South
2249877		T.aestivum	South
2249878		T.aestivum	South
2249879		T.aestivum	South
2249880	√	T.aestivum	South
2249881		T.aestivum	South
2249882	√	T.aestivum	South
2249883	√	T.aestivum	South
2249884		T.aestivum	South
2249885		T.aestivum	South
2249886		T.aestivum	South
2249887		T.aestivum	South
2249888		T.aestivum	South
2249889		T.aestivum	South
2249890		T.aestivum	South
2249891		T.aestivum	South
2249893	√	T.aestivum	South
2249894		T.aestivum	South
2249895		T.aestivum	South
2249896		T.aestivum	South
2249897		T.aestivum	South
2249898		T.aestivum	South
2249899		T.aestivum	South
2249900		T.aestivum	South
2249901		T.aestivum	South
2249904		T.aestivum	South

Supplementary Table 3: Comparison of the mean and variance of the complete set and the core set for phenotypic traits. Comparisons are the value of the complete set/value of core set.

Variable	Environment	Mean (population/core)	Variance (population/core)
Plant height	heat	1.02	0.81
Days to heading	heat	1	1
Days to maturity	heat	1	1
Days to heading	drought	1	1.02
Days to maturity	drought	1	1.01
Yield-gm^2	drought	0.99	0.95
Days to heading	irrigated	1	0.96
Plant height	irrigated	1.01	0.9
1000 kernel weight	quality	0.98	0.98
Test W-q	quality	1	1
Kernel length	quality	0.99	1
Kernel width	quality	1	0.99
Yellow berry	quality	0.92	1.04
Grain hardness	quality	1	0.99
Grain protein	quality	1.01	1.01

Supplementary Table 4: Mexican landraces identified as yellow rust resistant

GID	Accession	YR (%) in Mexico	YR (%) in India	Genetic groups
225133	CHIH95.5.18	20	10	6
225140	CHIH95.5.23	10	10	6
266720	DGO95.3.8	20	10	5
192328	MEX94.29.105	10	20	1
221777	MEX95.2.16	20	10	10
221779	MEX95.2.18	20	20	10
157704	OAX93.1.1.1	20	5	7
	Susceptible check	100	100	

Supplementary table 5: Details of markers with fixed alleles in landrace accessions belonging to groups based on longitude and latitude.

This table is presented in a separate excel file.

Supplementary Table 6: Table presenting clustering based on longitude, latitude and altitude with respect to genetic groups. Cluster 4 corresponds to genetic groups (6 and 14) having accessions from Chihuahua. Cluster 2 corresponds to genetic groups harboring accessions from Central Valley. Lat = Mean Latitude, Long = Mean Longitude and Alt = Mean Altitude. Out of 7986 accessions 656 were omitted due to missing data and 7330 genotypes were analyzed.

Cluster	Lat	Long	Alt	Genetic groups														
				1	4	10	2	13	7	9	12	6	14	3	8	11	5	15
3	24.9	-103	1701	0	128	144	0	0	0	0	0	298	136	0	0	640	83	39
4	28.1	-108.5	911	0	0	0	0	0	0	0	0	156	24	0	0	0	0	0
2	19.5	-99	2854	308	66	0	0	0	25	100	31	0	0	0	0	0	0	0
5	19.5	-99	2567	344	1067	132	412	13	346	938	293	0	0	0	195	0	0	0
1	19.1	-99.2	2244	574	184	14	83	274	0	0	0	0	0	283	0	0	0	0

Supplementary table 7: Detailed information of 7986 hexaploid wheat landrace accessions used for analysis in present study.

This table is presented in a separate excel file.

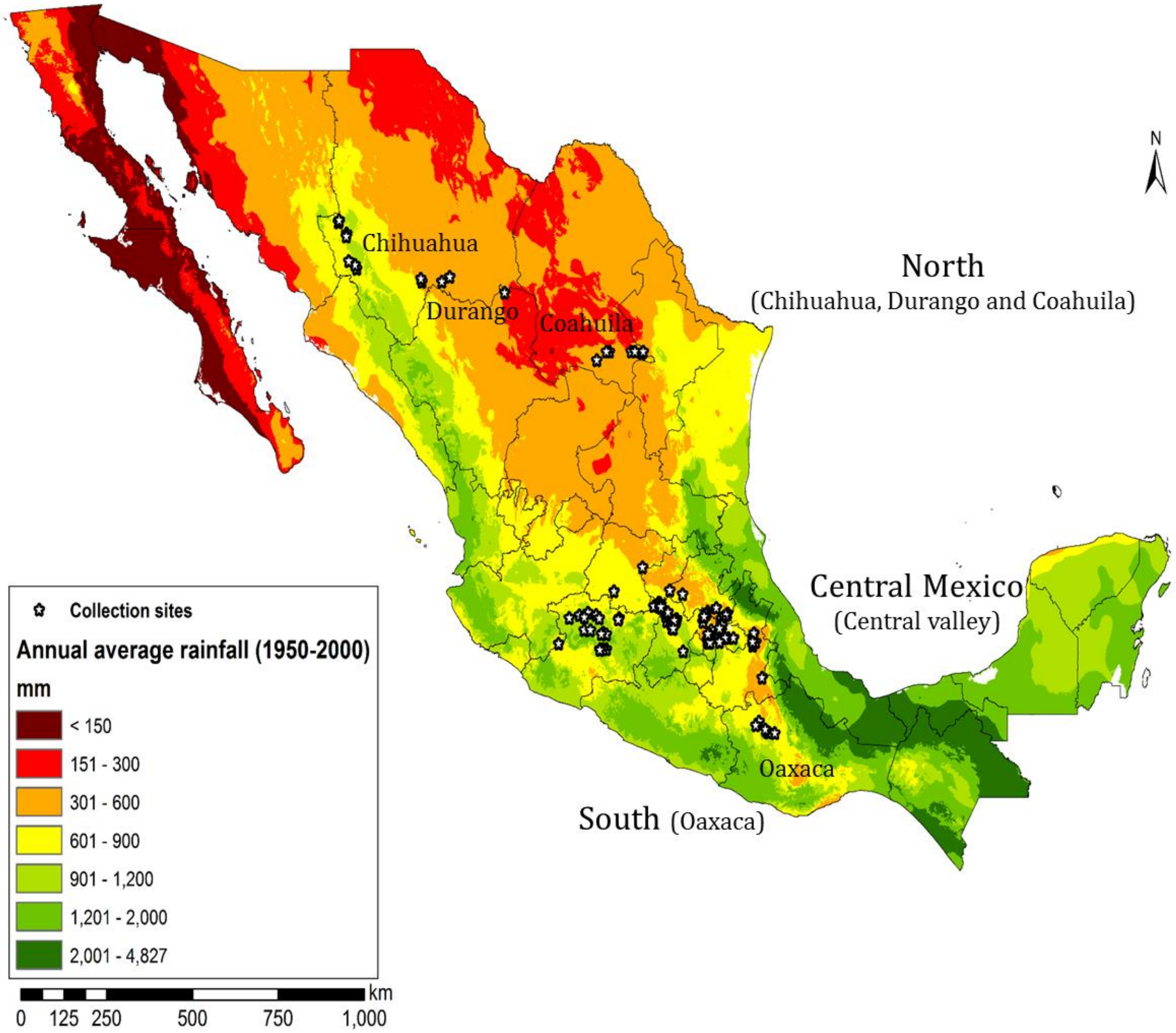

Supplementary Figure 1: Annual average rainfall in different regions of Mexico. Annual rainfall data of 50 years (1951 – 2000) was used to construct the graph. Map was made using ESRI's ArcGIS Desktop ArcMap 10.2.2 software (36; URL: http://resources.arcgis.com/en/help/). The dataset used to make the map was downloaded from publically available source, Worldclim 1.4 (35, 37, Available at www.worldclim.org)

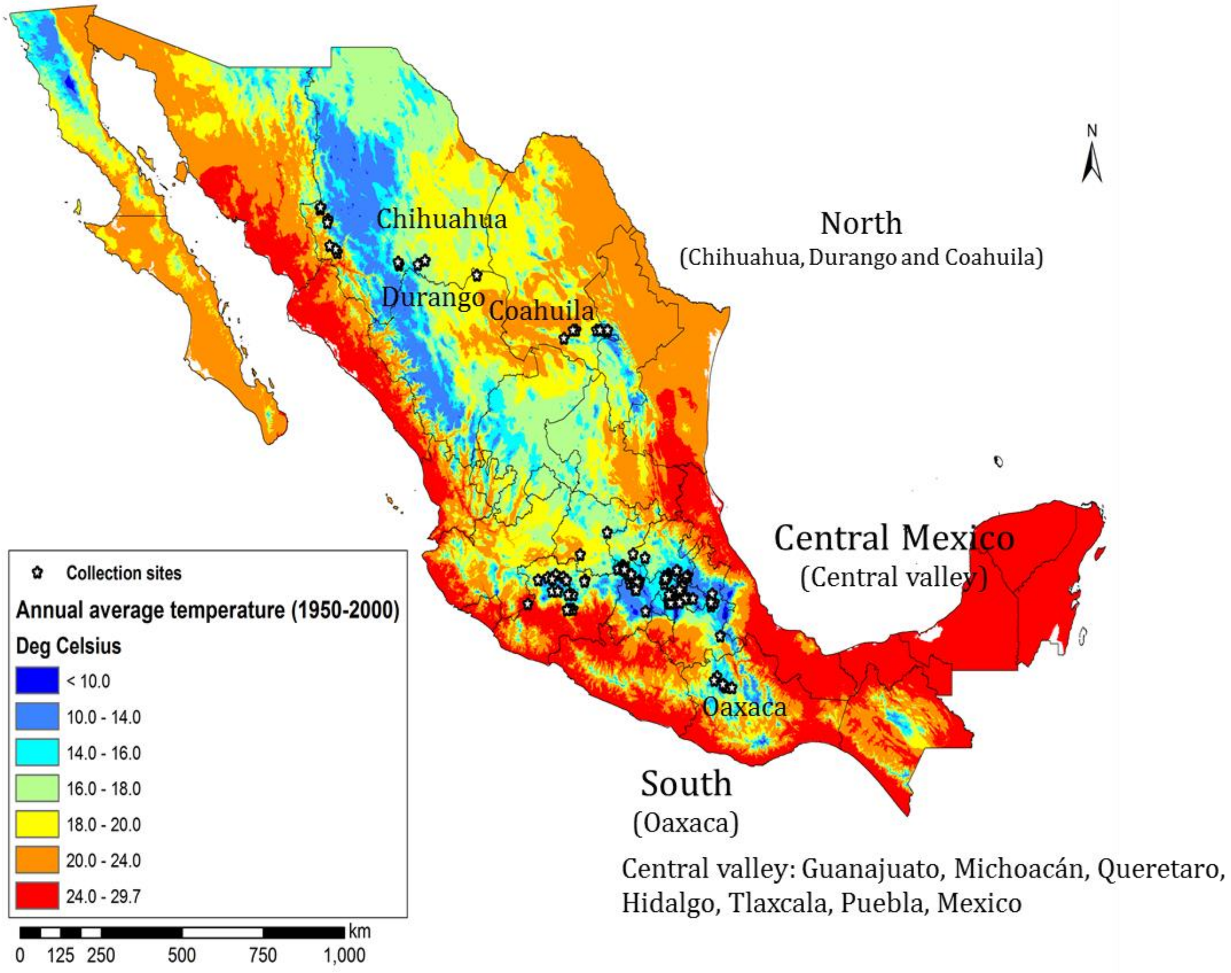

Supplementary Figure 2: Annual average temperature in different regions of Mexico. Daily mean temperature data of 50 years (1951 – 2000) was used to construct the graph. Map was made using ESRI's ArcGIS Desktop ArcMap 10.2.2 software (36; URL: http://resources.arcgis.com/en/help/). The dataset used to make the map was downloaded from publically available source, Worldclim 1.4 (35, 37, Available at www.worldclim.org)

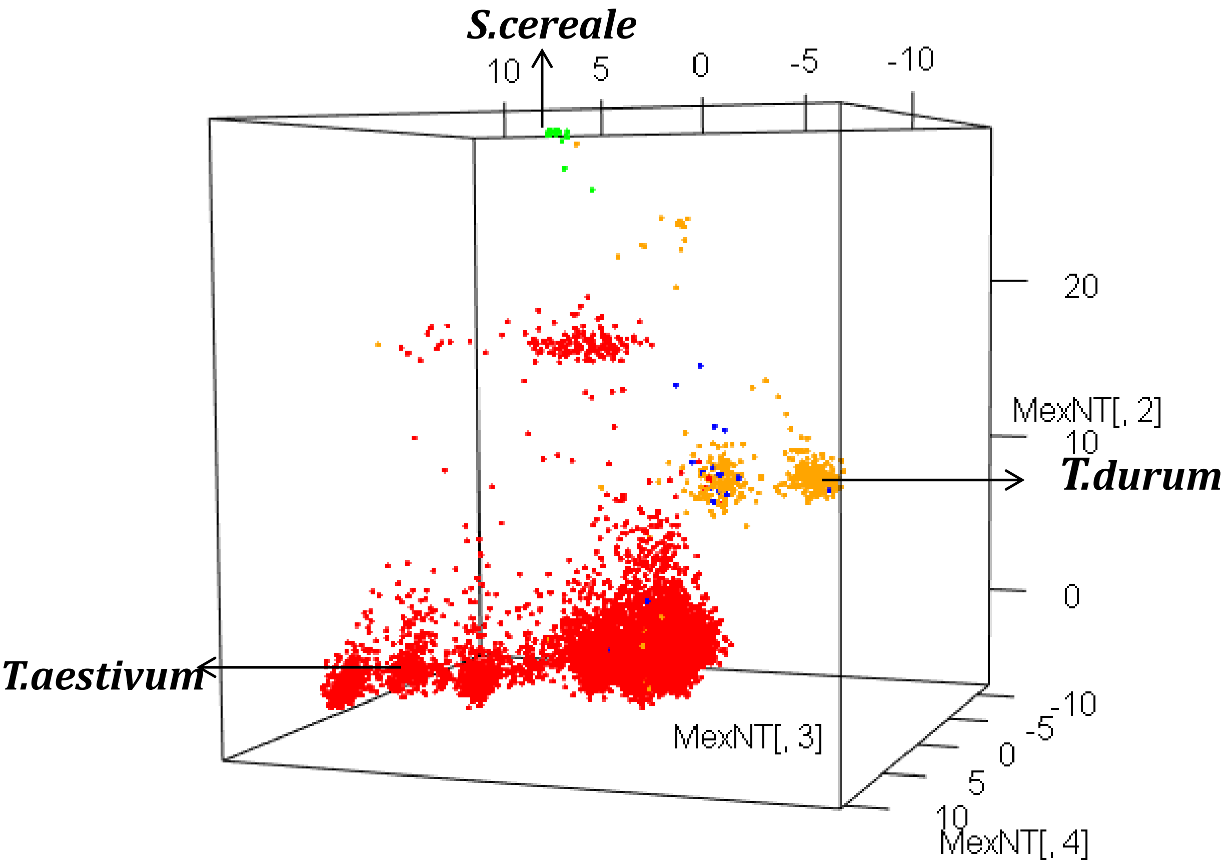

Supplementary Figure 3: Three-dimensional PCA graph showing the distribution of Mexican landrace accessions. Red = *T. aestivum*, Orange = *T. durum*, Green = *S. cereale*, Blue = *X. Triticosecale*

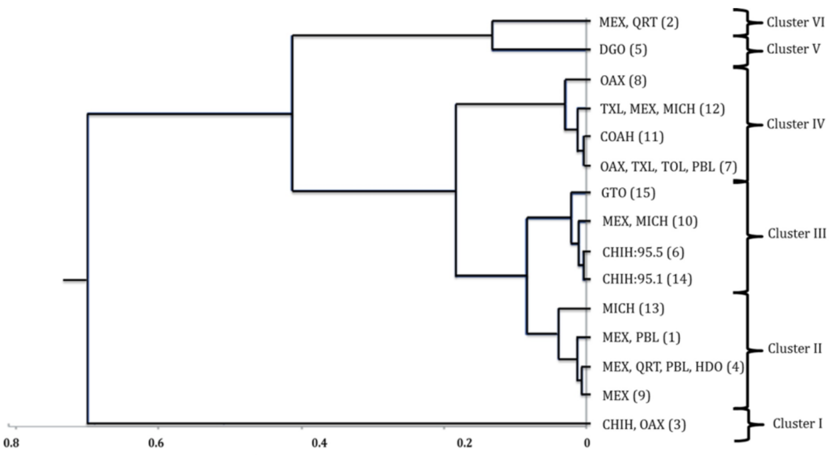

Supplementary Figure 4: Dendrogram showing relationships between 15 Mexican hexaploid landrace groups based on genetic classification (using Euclidean distances). The 15 groups were divided in to 6 clusters. There were total 15 groups which correspond to different states of Mexico. 1= (MEXICO, PUEBLA), 2 = (MEXICO, QUERETARO), 3 = (CHIHUAHUA, OAXACA), 4 = (MEXICO, PUEBLA, QUERETARO, HIDALGO), 5 = (DURANGO), 6 = (CHIHUAHUA 95.5), 7 = (OAXACA, TLAXCALA, TOLUCA, PUEBLA), 8 = (OAXACA), 9 = (MEXICO), 10 = MEXICO, MICHOACAN), 11 = (COAHUILA), 12 = (TLAXCALA, MEXICO, MICHOACAN), 13 = (MICHOACAN), 14 = (CHIHUAHUA 95.5), 15 = (GUANAJUATO).

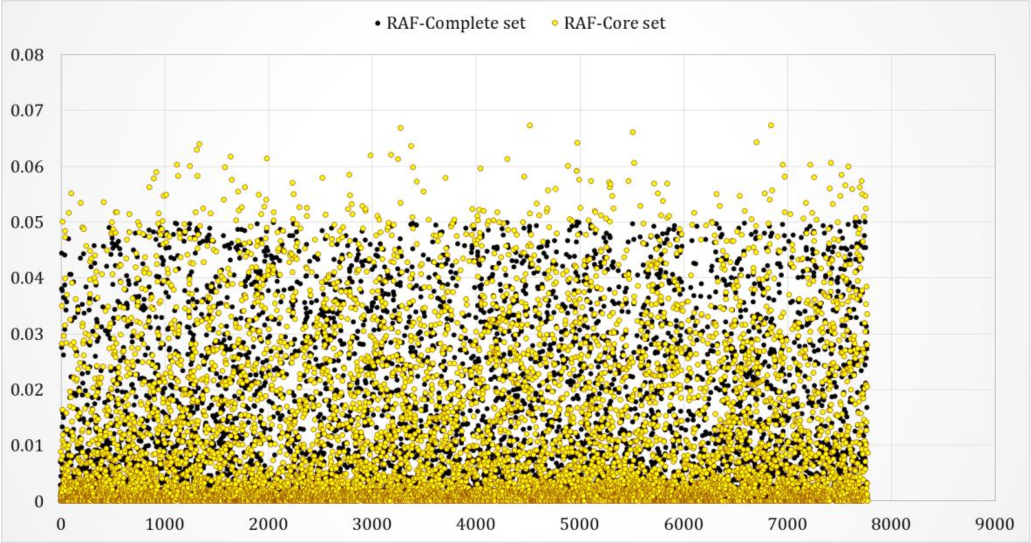

Supplementary Figure 5: Frequency of rare alleles (of complete set) in complete set and core reference set. Rare allele frequency and individual markers (7775 SNPs) are plotted on the Y and X axes, respectively. The figure also shows that the frequency of some alleles (which were <0.05 in the complete set) increased in core reference sets.

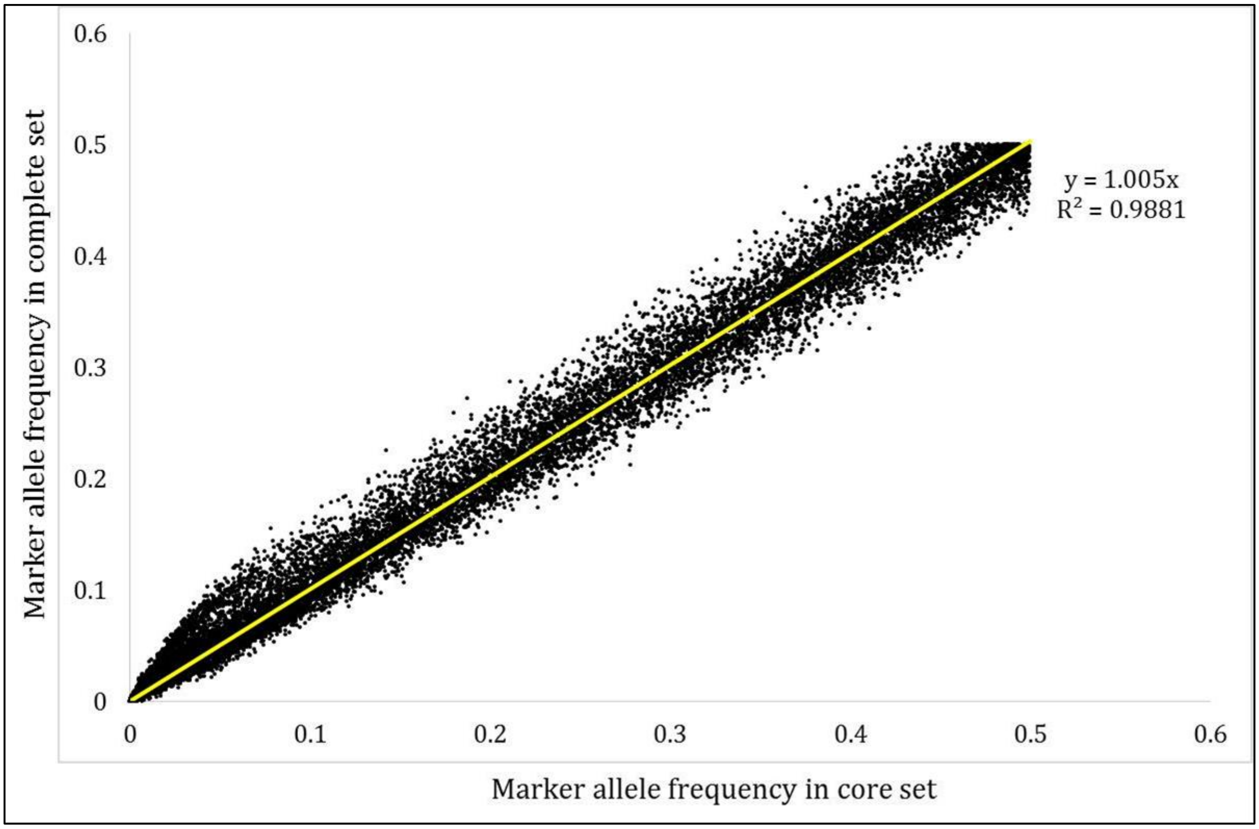

Supplementary Figure 6: The relationships between marker allele frequencies of complete and core reference set accessions of Mexican hexaploid landraces. Marker allele frequencies in complete and core reference sets are plotted on the Y and X axes, respectively. Minor allele of each marker with a frequency ≤ 0.5 was taken into account in the analysis.

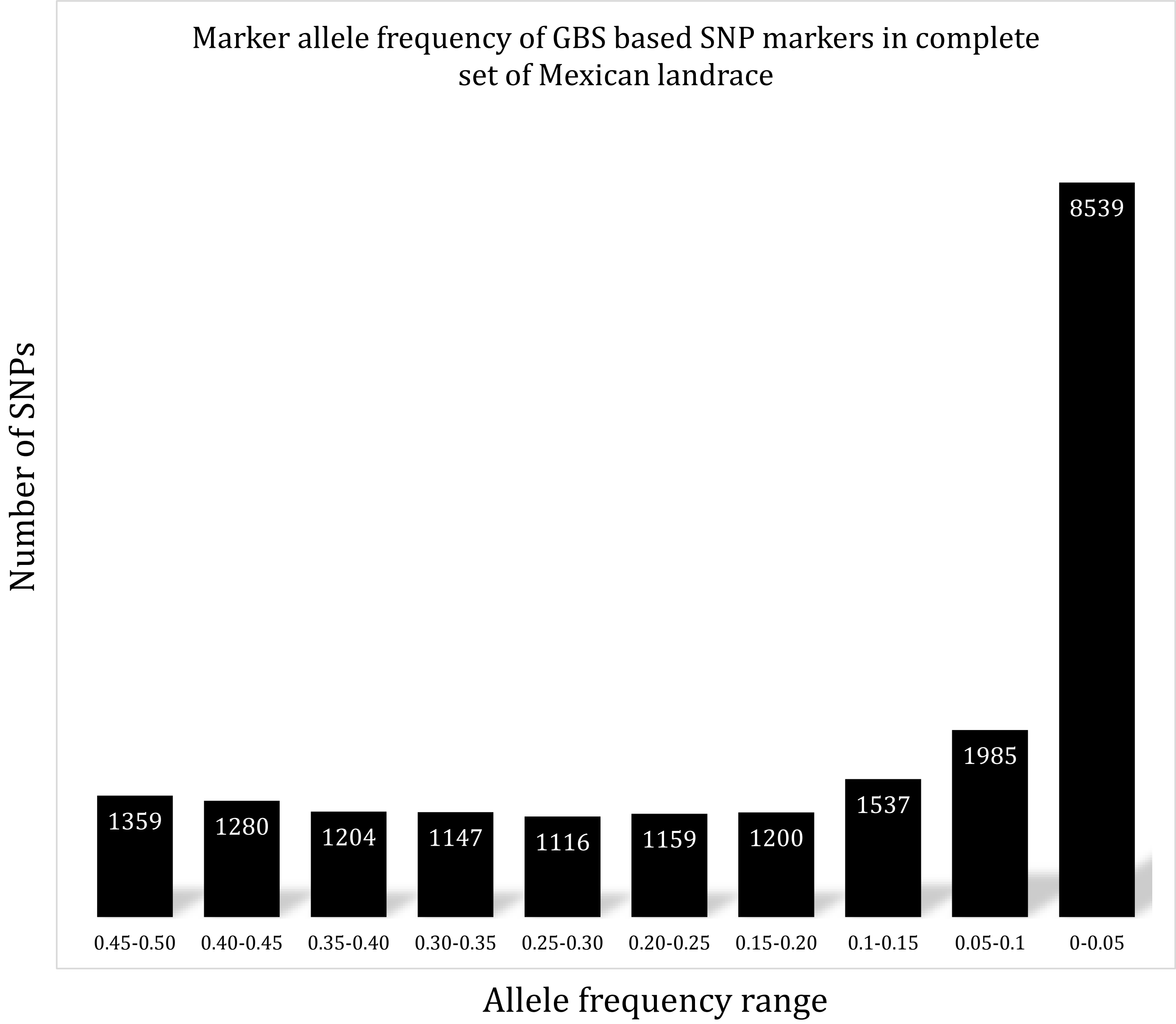

Supplementary Figure 7: The number of SNP markers with different allele frequencies. The number of markers and marker allele frequency ranges are plotted on the Y and X axes, respectively. Minor allele of each marker with a frequency ≤ 0.5 was taken into account in the analysis.

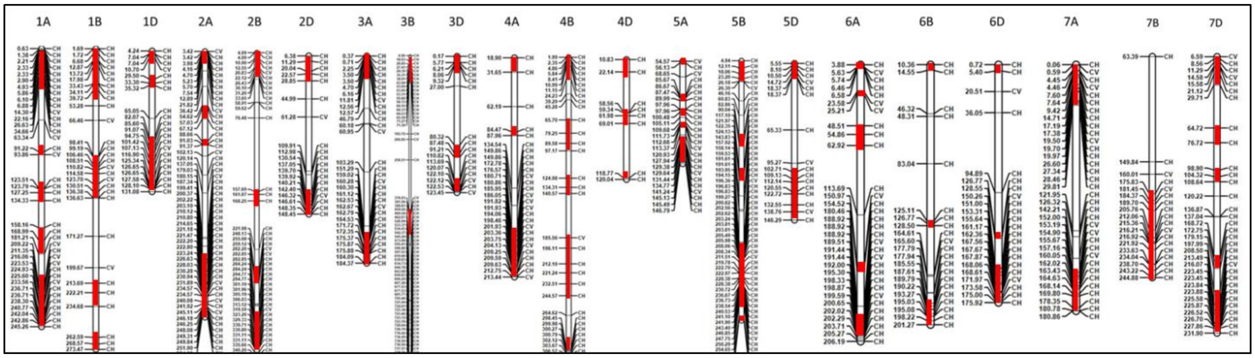

Supplementary Figure 8: Genomic regions on 21 wheat chromosomes having fixed alleles in Mexican landrace accessions of Chihuahua and Central Valley region.

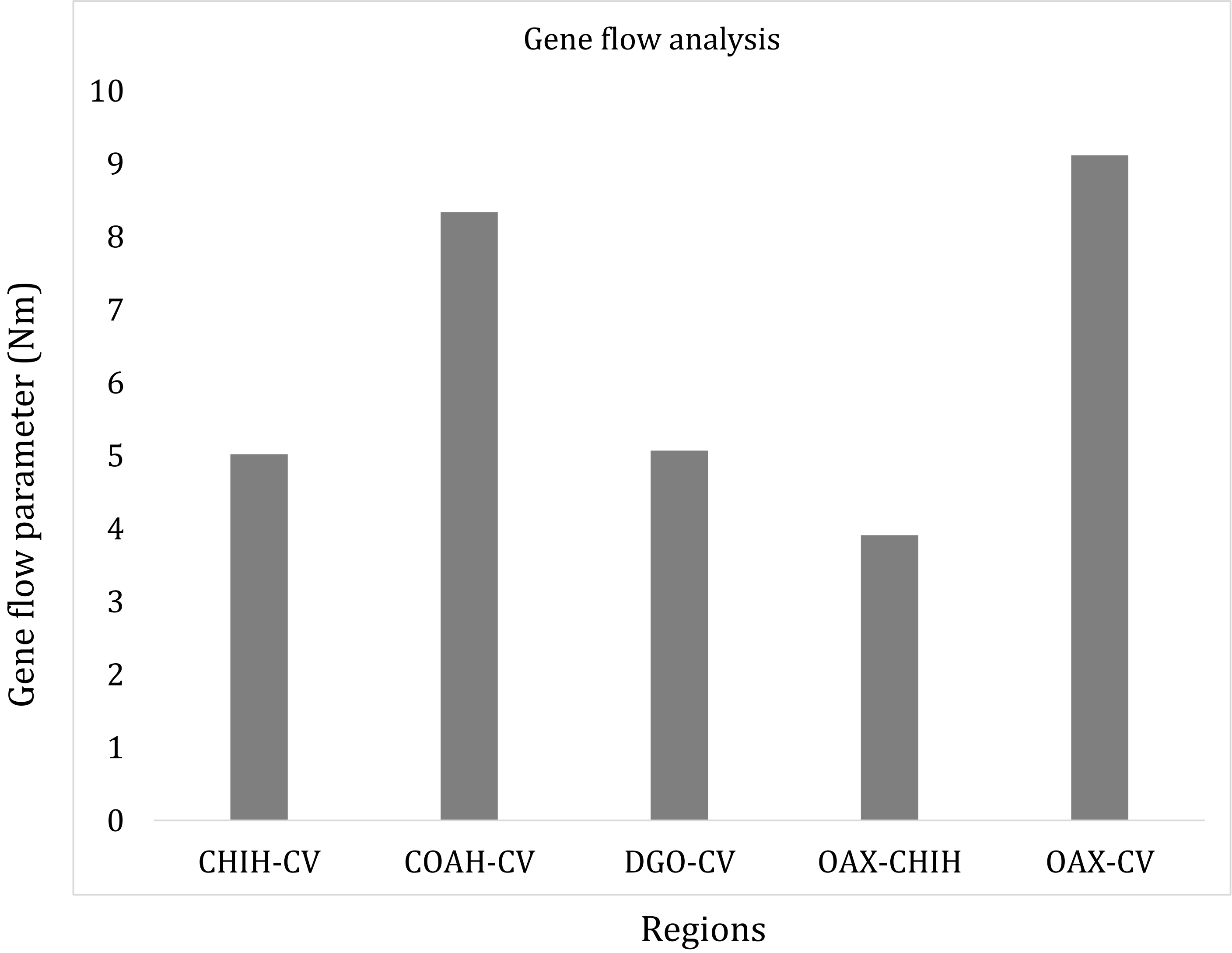

Supplementary Figure 9: Gene flow between hexaploid landrace accessions belonging to different regions of Mexico. Maximum gene flow was observed between accessions of Oaxaca and the Central Valley, while minimum gene flow was observed between accessions of Chihuahua and Oaxaca. Central valley (CV) = MEXICO, PUEBLA, QUERETARO, HIDALGO, TLAXCALA, TOLUCA, MICHOACAN; CHIH = CHIHUAHUA; DGO = DURANGO; COAH = COAHUILA; OAX = OAXACA. Nm = Gene flow parameter (product of population size and fraction of migrants).

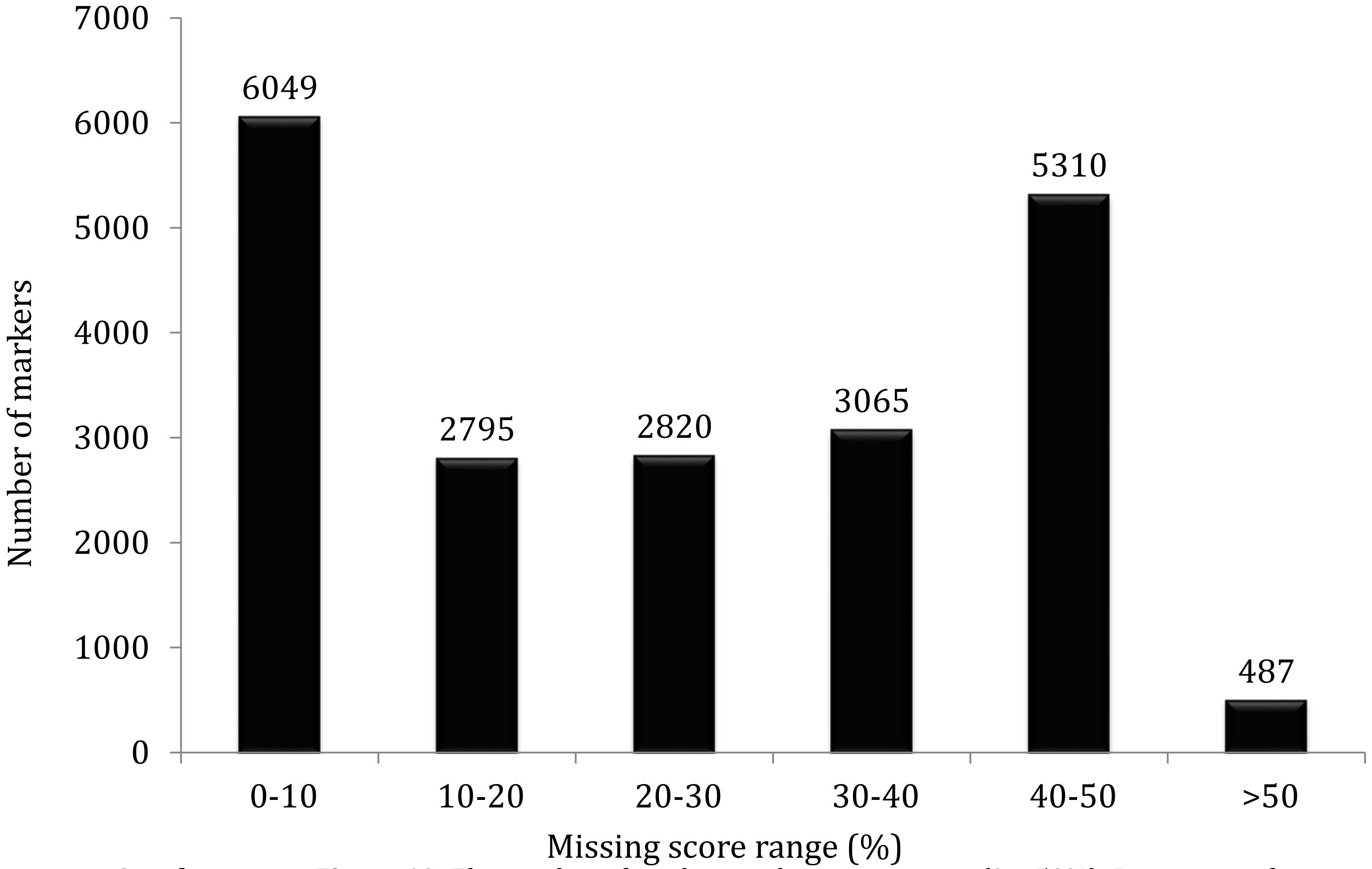

Supplementary Figure 10: The number of markers with missing scores (0 – 50%). Percentage of missing scores and number of markers are shown on the X and Y axes, respectively. The 487 markers with >50% missing scores were omitted from the analysis.

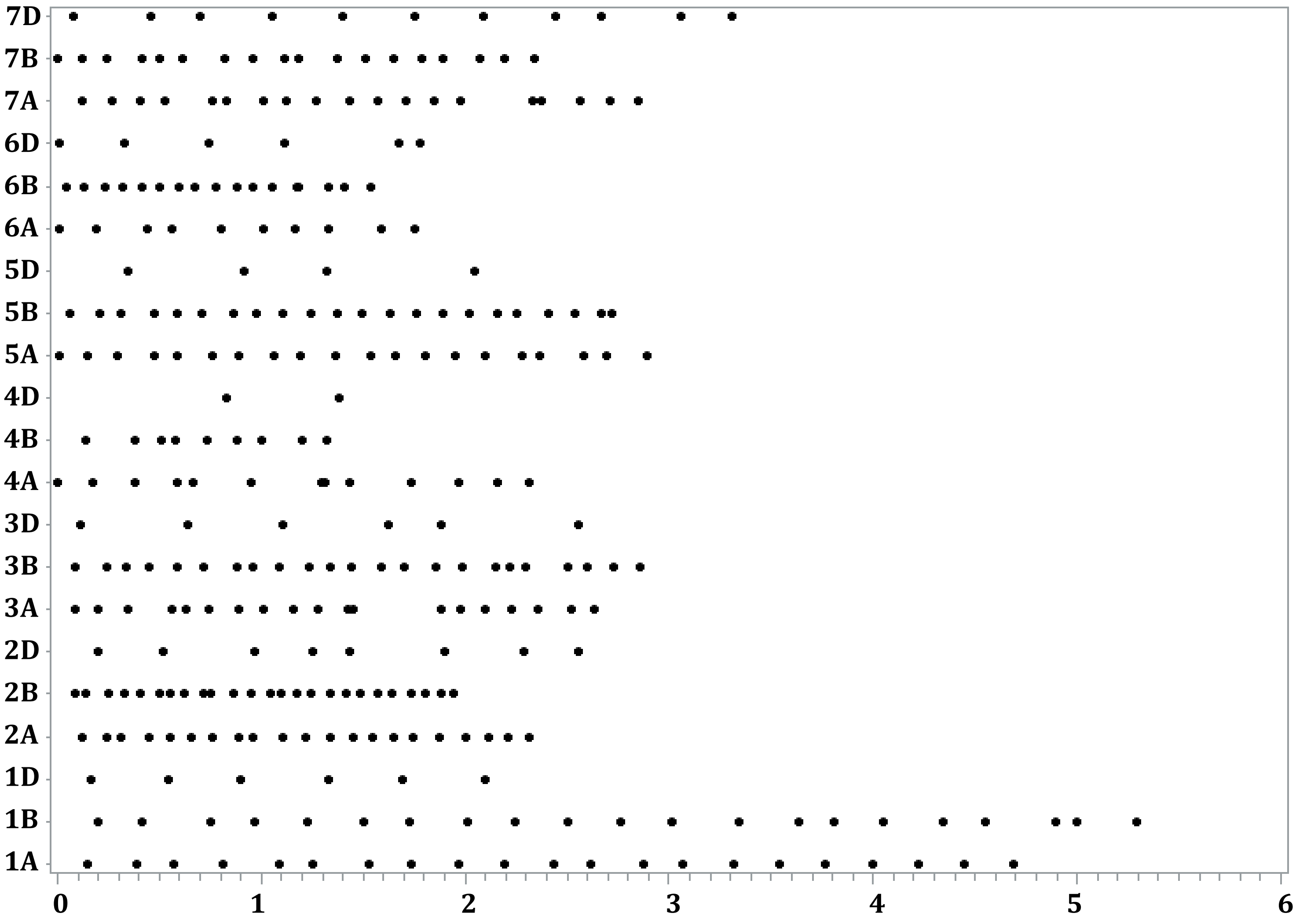

Supplementary Figure 11: The distribution of selected markers (at nearly equal distances) on each chromosome. Chromosome number and genetic distances (Morgan) are plotted on the Y and X axes, respectively. The figure shows that selected markers were evenly distributed throughout the genome.

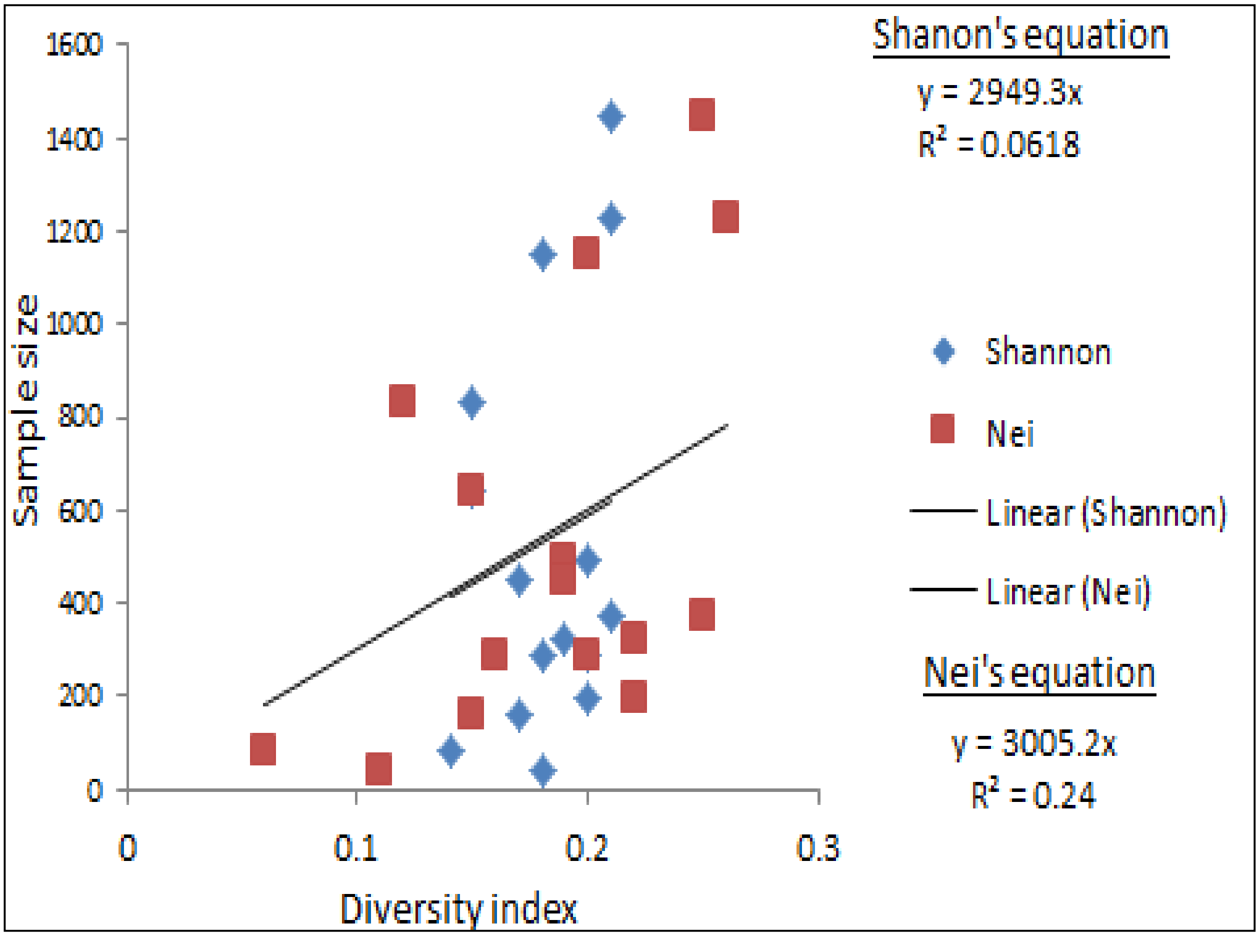

Supplementary Figure 12: Correlation analysis of diversity indices and sample size. The sample sizes of 15 genetic groups and their corresponding diversity indices (Nei's and Shanon's) are plotted on the Y and X axes. This graph suggests that genetic groups of Mexican landraces do not affect diversity.

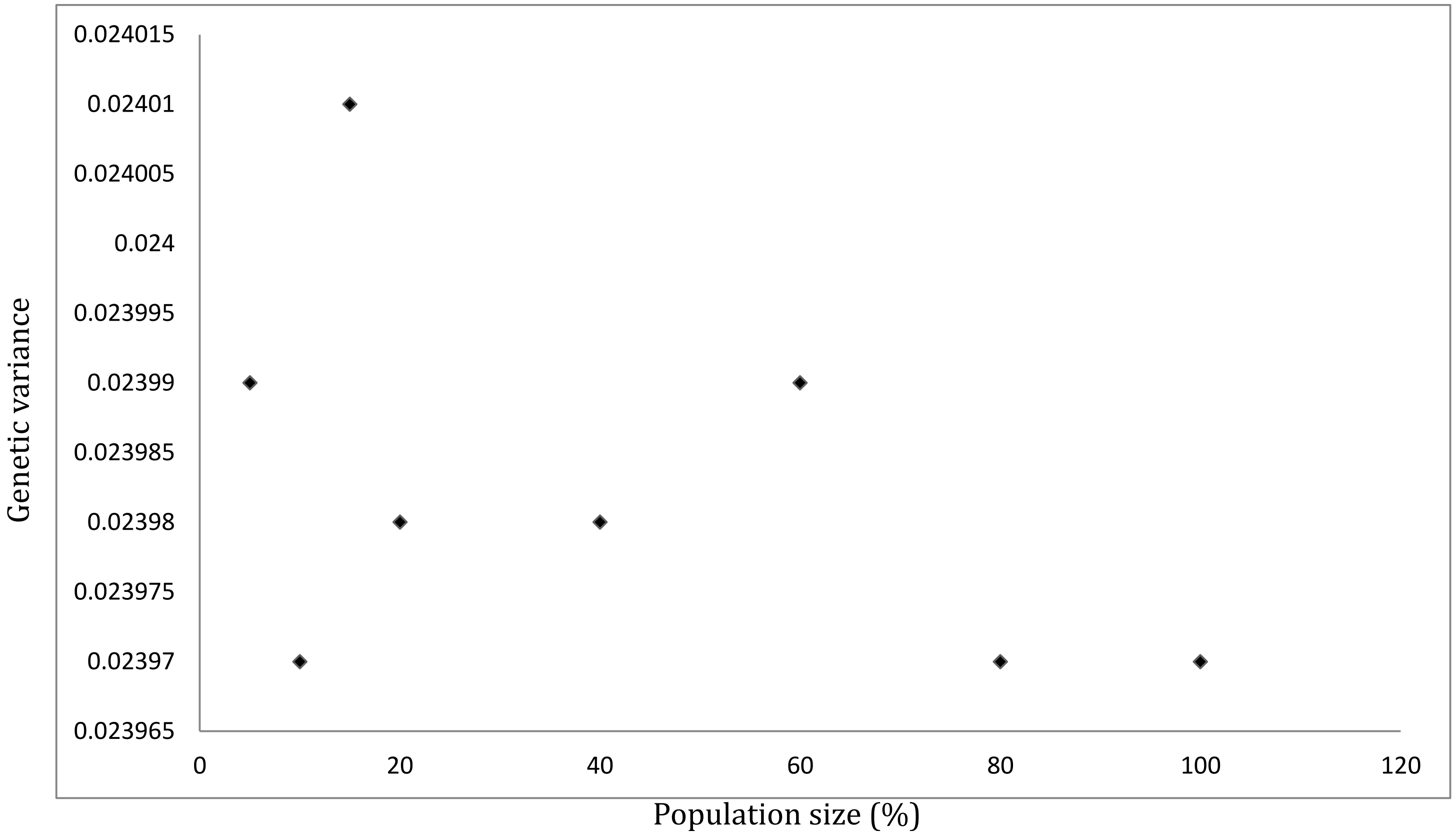

Supplementary Figure 13: Simulation results for estimating the optimum population size of the core reference set. Genetic variances and population sizes (%) are plotted on the Y and X axes. The graph suggests that a core reference set of approximately 15% is best.
